# Early and intensive Motor Training for people with spinal cord injuries (the SCI-MT Trial): description of the intervention

**DOI:** 10.1038/s41393-023-00911-4

**Published:** 2023-07-19

**Authors:** M. Ben, J. V. Glinsky, J. Chu, A. I. Spooren, S. Roberts, L. W. Chen, S. Denis, M. Lorusso, V. Jorgensen, E. J. Gollan, J. Agostinello, C. C. M. Van Laake-Geelen, C. Lincoln, J. M. Stolwijk, C. Bell, S. Paddison, D. Rainey, K. Tranter, J. Ilha, K. Oostra, C. Sherrington, L. A. Harvey

**Affiliations:** 1 Kolling Institute, Faculty of Medicine and Health, The University of Sydney, Sydney, NSW Australia; 2 John Walsh Centre for Rehabilitation Research, Northern Sydney Local Health District, St Leonards, Sydney, NSW Australia; 3 REVAL, Hasselt University, Hasselt, Belgium; 4 Fiona Stanley Hospital, Murdoch, WA Australia; 5 Royal North Shore Hospital, St Leonards, NSW Australia; 6 The Prince of Wales Hospital, Wales, NSW Australia; 7 I.R.C.C.S. Foundation Santa Lucia, Rome, Italy; 8 Sunnaas Rehabilitation Hospital, Nesodden, Norway; 9 The Princess Alexandra Hospital, Harlow, QLD Australia; 10 The Royal Talbot Rehabilitation Centre, Kew Vic, VIC Australia; 11 Adelante Centre of Expertise in Rehabilitation and Audiology, Hoensbroek, The Netherlands; 12 Department of Rehabilitation Medicine, Research School CAPHRI, Maastricht University, Maastricht, The Netherlands; 13 Queen Elizabeth National Spinal Injures Unit, Glasgow, Scotland; 14 Center of Excellence for Rehabilitation Medicine, University Medical Center Utrecht Brain Center, University Medical Center Utrecht and De Hoogstraat Rehabilitation, Utrecht, The Netherlands; 15 Spinal Cord Injury Rehabilitation, Repat Health Precinct, Daw Park, SA Australia; 16 London Spinal Cord Injury Centre, Royal National Orthopaedic Hospital Trust, Middlesex, UK; 17 Royal Rehab, Ryde, NSW Australia; 18 Universidade do Estado de Santa Catarina – UDESC, College of Health and Sport Science, Florianopolis, SC Brazil; 19 Ghent University Hospital, Ghent, Belgium; 20 Sydney School of Public Health, Faculty of Medicine and Health, University of Sydney, Sydney, NSW Australia

**Keywords:** Neurology, Medical research

## Abstract

**Study design:**

Descriptive.

**Objectives:**

The primary objective is to describe the intervention that will be provided in a large multi-centre randomised controlled trial titled: *Early and Intensive Motor Training for people with Spinal Cord Injuries* (the SCI-MT Trial). The secondary objective is to describe the strategies that will be used to operationalise and standardise the Motor Training provided to participants while keeping the intervention person-centred.

**Methods:**

The paper focuses on the rationale and principles of Motor Training for people with spinal cord injuries (SCI). The description of the intervention is based on the Template for Intervention Description and Replication (TIDieR) checklist. Specifically, it addresses the following 6 criteria of the TIDieR checklist: why the effectiveness of Motor Training is being examined; what, how, where and when the Motor Training will be administered; and how much Motor Training will be provided.

**Results:**

A detailed intervention manual has been developed to help standardise the delivery of the intervention.

**Conclusions:**

This paper describes the details of a complex intervention administered as part of a large randomised controlled trial. It will facilitate the subsequent interpretation of the trial results and enable the intervention to be reproduced in clinical practice and future trials.

## Introduction

A key aim of physiotherapy for people with recent spinal cord injuries (SCI) is to maximise neurological recovery and function [1]. The most promising treatment for maximising recovery is Motor Training [2]. We are using *Motor Training* as an umbrella term to describe a specific type of exercise program that consists of task-specific training supplemented with strength training. Motor Training is a complex intervention that needs to be individualised for each person according to their impairments, abilities, and goals. That is, there is no one specific set of Motor Training exercises that can cater for all people with SCI.

Intensive Motor Training is advocated for its potential to promote recovery and improve function, particularly in those with some neurological preservation below the level of the injury and if provided soon after injury. The evidence supporting intensive Motor Training in people with recent SCI comes from animal studies and from the stroke literature [3–5]. However, these findings are yet to be replicated within a high-quality clinical trial. A trial called *Early and Intensive Motor Training for people with Spinal Cord Injuries* (The SCI-MT Trial) is currently underway. It is a multi-centre international pragmatic randomised controlled trial (RCT) involving 15 sites across 7 countries in Europe and Australia. Two hundred and twenty participants will be randomised to either usual care or usual care plus 10 weeks of intensive Motor Training. Patients will only be eligible if they are within 10 weeks of injury and have preservation of motor function below the level of injury. That is, people with motor function more than three levels below the motor level (for those with AIS A lesions) or with motor incomplete lesions (AIS C and AIS D) will be included in the trial. The primary outcome of the trial is neurological recovery as reflected in overall strength (measured with the Total Motor Score on the International Standards for Neurological Classification of SCI). The secondary outcomes include other measures of neurological recovery and function. To date, 120 participants have been randomised. The trial protocol is described elsewhere [6]. The protocol for the intervention of the SCI-MT Trial (the focus of this paper) was developed by 5 members of the research team (MB, LAH, JVG, JC, KT) who had both clinical and academic experience in SCI and physiotherapy. All members of the wider research team were also consulted.

Most physiotherapists and occupational therapists are highly trained in delivering the two components of Motor Training as provided in the SCI-MT Trial, namely task-specific and strength training. However, the challenge for the SCI-MT Trial is ensuring that the trial therapists administer Motor Training in a consistent way following some key principles whilst individualising it to the needs of each person, and that the therapists deliver the intervention to the highest possible standard. The other challenge is communicating the contents of this complex intervention to the scientific community, and particularly to those without a background in physiotherapy (or occupational therapy) in accordance with the Template for Intervention Description and Replication (TIDieR) guidelines [7]. Reporting of complex interventions is important so that the subsequent trial results can be easily interpreted and so that the intervention can be reproduced in clinical practice or future research. The TIDieR checklist provides a guide to the reporting of complex interventions [7] and underpins our description of the SCI-MT Trial intervention (see Table 1). The aim of this paper is to describe the details of the Motor Training intervention provided during the SCI-MT Trial. Specifically, the intervention will be described according to the following 6 criteria of the TIDieR checklist: why the effectiveness of Motor Training is being examined; what, how, where, and when the Motor Training will be administered; and how much Motor Training will be provided. The paper will address each of these criteria in two sections, namely (i) the rationale, evidence, and key principles supporting Motor Training and, (ii) operationalising and standardising the Motor Training provided as part of the SCI-MT Trial.

**Table 1:** Intervention description of the SCI-MT trial using the Template for Intervention Description and Replication (TIDieR) checklist.

Brief Name	• Early and Intensive Motor Training for People with Spinal Cord Injuries (the SCI-MT Trial)
Why	• The most promising treatment for maximising recovery of function in people with recent spinal cord injuries (SCI) is Motor Training. Motor Training is a term we are using to describe task-specific training supplemented with strength training. A high intensity of Motor Training directed at and below the level of injury is advocated soon after SCI, however, the effectiveness of such interventions has not been investigated with a clinical trial to date. The SCI-MT Trial has been undertaken to investigate the effectiveness of early and intensive Motor Training in people with SCI.
What materials	• A training package has been developed for the SCI-MT intervention therapists. The 2 h package (delivered via Zoom) conveys the key principles and guidelines of the intervention. • An Intervention Manual has been developed as a supplement to the training package (see Supplementary file 1). It details the key training principles of the SCI-MT Trial, information about the use of Practice Sheets, the type of equipment that can be used, and the staff who can deliver the 12 h of Motor Training per week. The manual provides examples of exercises that can be used to deliver Motor Training, as well as completed Practice Sheets using four hypothetical case studies. • Practice Sheets will be used by the SCI-MT intervention therapists and participants together to set exercise targets, and to document exercise intensity, set-up, and duration. Dosage parameters documented in the Practice Sheets will be reviewed during each session, and these will be used to encourage the participants to work as hard as they can, and to progress the exercises from day to day, week to week.
What procedures	• Intervention therapists will review and upgrade weekly goals with the participants. The weekly goals will be related to the four baseline goals identified at the initial assessment. • Motor Training will be individualised to the participants’ impairments, goals, and needs. The intervention therapists will prescribe exercises based on their clinical decision making whilst adhering to the key principles of the SCI-MT Trial protocol. • Practice Sheets will be used to capture exercise set-up, dosage, and intensity for every session. • SCI-MT trial staff will review Practice Sheets at every training site and provide ongoing feedback and training to the intervention therapists to ensure adherence to key principles.
Who provided	• The SCI-MT intervention will be delivered by a healthcare professional (e.g., Physiotherapist, Occupational Therapist, Exercise Physiologist) appropriately qualified to deliver all or some aspects of Motor Training once they have completed the compulsory training.
How and Where	• The SCI-MT intervention will be delivered in one-to-one sessions in a therapy gym. The therapists at each site can use any equipment they deem appropriate to deliver Motor Training. Equipment such as robotics, bodyweight support treadmill training, and electrical stimulation can be used during SCI-MT sessions, as long as the key principles of Motor Training are adhered to.
When and How much	• The intervention participants will receive 12 h of Motor Training in addition to usual care, for 10 weeks. • The number and duration of sessions per week is not stipulated but typically the 12 h per week will be provided over 5 to 10 sessions of 1 to 2 h duration each.
Tailoring	• The intervention will be individualised to each participant according to their impairments, abilities, and goals. Regular reassessments of participants, weekly goal setting, and meticulous documentation of the intervention details in the Practice Sheets during the 10-week period will ensure ongoing progression and tailoring of the Motor Training program for each participant.
How well	• The fidelity of the intervention will be examined in detail with a process evaluation that will run alongside the trial.

## Methods/Results

### Part 1: rationale, evidence and key principles supporting Motor Training in the SCI-MT Trial

The Motor Training provided during the SCI-MT Trial is directed at increasing motor function at and below the level of injury. This will be measured using the Total Motor Score of the International Standards for the Neurological Classification of SCI. For example, in a person with partial paralysis of the lower limbs, the Motor Training (i.e., task-specific training and strength training) will be used to improve the ability to stand or walk as well as to increase lower limb muscle strength. Of course, Motor Training as typically provided in rehabilitation can also be targeted above the level of injury. For example, it can be used to improve the ability of a person with paraplegia to transfer through their neurologically intact upper limbs. However, this is not the focus of the SCI-MT Trial. Rather, the focus is on improving motor function at and below the level of injury with the primary aim of increasing total motor scores.

The Motor Training provided during the SCI-MT Trial is also directed at helping people regain the ability to move in a way that is as close as possible to that of a non-disabled person. For example, gait training will be directed at walking without aids and orthoses if possible. Similarly, upper limb training will focus on regaining the ability to reach and grasp with the kinematics and kinetics of a non-disabled person where possible. Needless to say, even small amounts of residual weakness may ultimately require some type of adaptation or compensation that alters the kinematics and kinetics of movement from that of a non-disabled person. The extent of adaptation or compensation will depend on the extent of neurological loss and may range from a slight variation to the need for extensive orthoses, aids, manual guidance, or assistance.

The SCI-MT Trial intervention will be provided in a high dosage. Dosage is determined by a combination of exercise intensity, duration, and frequency. Participants in the SCI-MT Trial will be required to exercise as hard as possible (i.e., at high intensities and with a high number of movement repetitions) for 12 h per week over 10 weeks (i.e., for a long duration and at a high frequency). There are no stipulations about the number of sessions per week but typically the 12 h are provided over 5 to 10 sessions of 1 to 2 h duration each.

The two components of Motor Training are described below

### Task-specific training

Task-specific training has its origins in the motor relearning approach which was widely advocated in rehabilitation by Carr and Shepherd in the 1980s [8, 9]. Behavioural and neurosciences have moulded its development over the years. Animal and human studies indicate that task-specific training is a strong stimulus for neuroplasticity both at the site of injury and throughout the neural pathways responsible for purposeful movement [10–12]. Task-specific training involves practice of movements that are specific to the desired functional outcome. The key principles of task-specific training are as follows.

#### Repetitions

Task-specific training requires repetitious practice of purposeful movements. The evidence for repetitious practice is compelling, particularly in the stroke literature [13–15]. Hundreds or even thousands of daily steps and upper limb movements are required to improve walking ability and arm function respectively after a stroke or SCI [13, 16, 17]. Nonetheless, patients undergoing rehabilitation in SCI units get very little repetitious practice [18]. This is primarily because patients do not spend the amount of time in therapy that is required for repetitious practice in addition to the many other types of interventions that they need during rehabilitation. The SCI-MT Trial aims to increase the amount of repetitious practice by adding an extra 12 h of therapy per week for 10 weeks.

#### Active (not passive)

Task-specific training involves active voluntary muscle contractions, specific to a functional task, with the aim of learning and improving the ability to perform that task. Movement practiced in this way involves cognitive processing which is believed to drive neuroplasticity. This is supported by animal [19] and human [20, 21] studies. Passive movements that are solely driven by the help of therapists, robotics, electrical stimulation, or other devices do not typically involve active muscle contractions and are unlikely to lead to such changes [22, 23]. However, assistive technologies are often used in conjunction with the effort of the patient to provide a form of task-specific training. For example, locomotor training with or without body weight support (BWS) on a treadmill can be used to augment patients’ attempts at walking [24, 25]. Similarly, upper or lower limb functional training using robotics has some features in common with task-specific training [23, 26, 27]. Activity Based Therapy (ABT) is another example. Although a clear definition of ABT is currently lacking [28], it typically focuses on high volumes of walking practice using various combinations of robotics and electrical stimulation [29–31]. A key underlying principle of the SCI-MT intervention is that only active exercises will be practiced during task-specific training although attempts at movement can be assisted by robotics, electrical stimulation, or any equipment available at the training sites and as deemed appropriate by the trial therapists.

#### Progression

Task-specific training needs to be progressed [32, 33]. This includes progressing the difficulty of part-task and whole-task practice. Walking on a treadmill is an example of whole-task practice. As the ability to walk improves, the difficulty of the training needs to be increased, with the practice expanded to different environments with increasing task complexity resulting in increased cognitive or physical demands [33]. Part-task practice focuses on repetition of components of the desired task, with a biomechanical analysis underpinning the choice of the specific components to be practiced [34]. Part-task practice of walking may include exercises such as stepping forwards, weight shifting in standing, or even a much simpler practice of knee and hip extension on a sliding tilt table (see www.physiotherapyexercises.com for over 1,500 exercises which include whole-task and part-task practice). Like whole-task practice, part-task practice also needs to be progressed and relies on reassembling the components back to the whole-task once the person is ready to do so [14]. The ongoing progression of task-specific practice is required to the point where the ability to perform the task becomes an automatic skill [35].

**Fig. 1:**
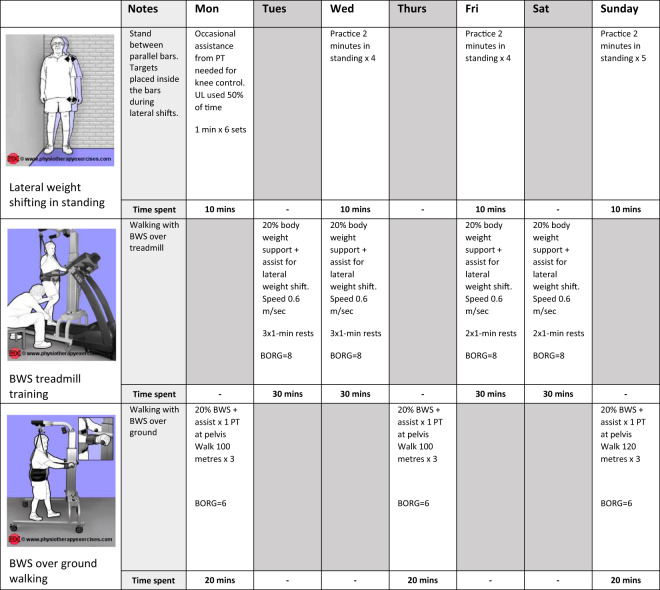
Example of a Practice Sheet used to document and progress the Motor Training. Images may be used as prompts for the exercises being practiced, with specific instructions or deviations documented in the “notes” column (Images copied with permission from www.physiotherapyexercises.com). PT physiotherapist, BWS body weight support, UL upper limbs, BORG Borg Rating Scale of Perceived Exertion.

The progression of task-specific training is critical to the SCI-MT Trial. Therapists increase the difficulty of the training as soon as an exercise can be successfully performed. The progression can be done in many ways. Examples of progression include increasing the number of repetitions, changing the environmental set-up to increase the challenge, or manipulating the difficulty of the task. Physiotherapists typically select new or additional exercises every few days or weeks and constantly modify existing exercises to ensure the training is challenging and progressed. Exercise selection is highly reliant on the therapists’ ongoing analysis of patients’ attempts at movement as well as therapists’ decision-making and clinical expertise. Appropriate progression is also reliant on careful and detailed recording of what a person does today in order to ensure they do more tomorrow. For this reason, Practice Sheets will be used for all training sessions in the SCI-MT Trial (see Fig. 1 for an example), where features of the exercise reflecting the intensity, set-up, and amount of practice will be recorded. For example, if a person repeatedly practices sit-to-stand, the following details may be recorded to direct the future progression: the height of the chair, the number of repetitions of sit-to-stand, the position of the feet in relation to the base of the chair, the amount of weight put through each foot, the time taken to complete a set number of repetitions, or some subjective measure of exertion such as the Borg Scale of perceived exertion.

**Fig. 2:**
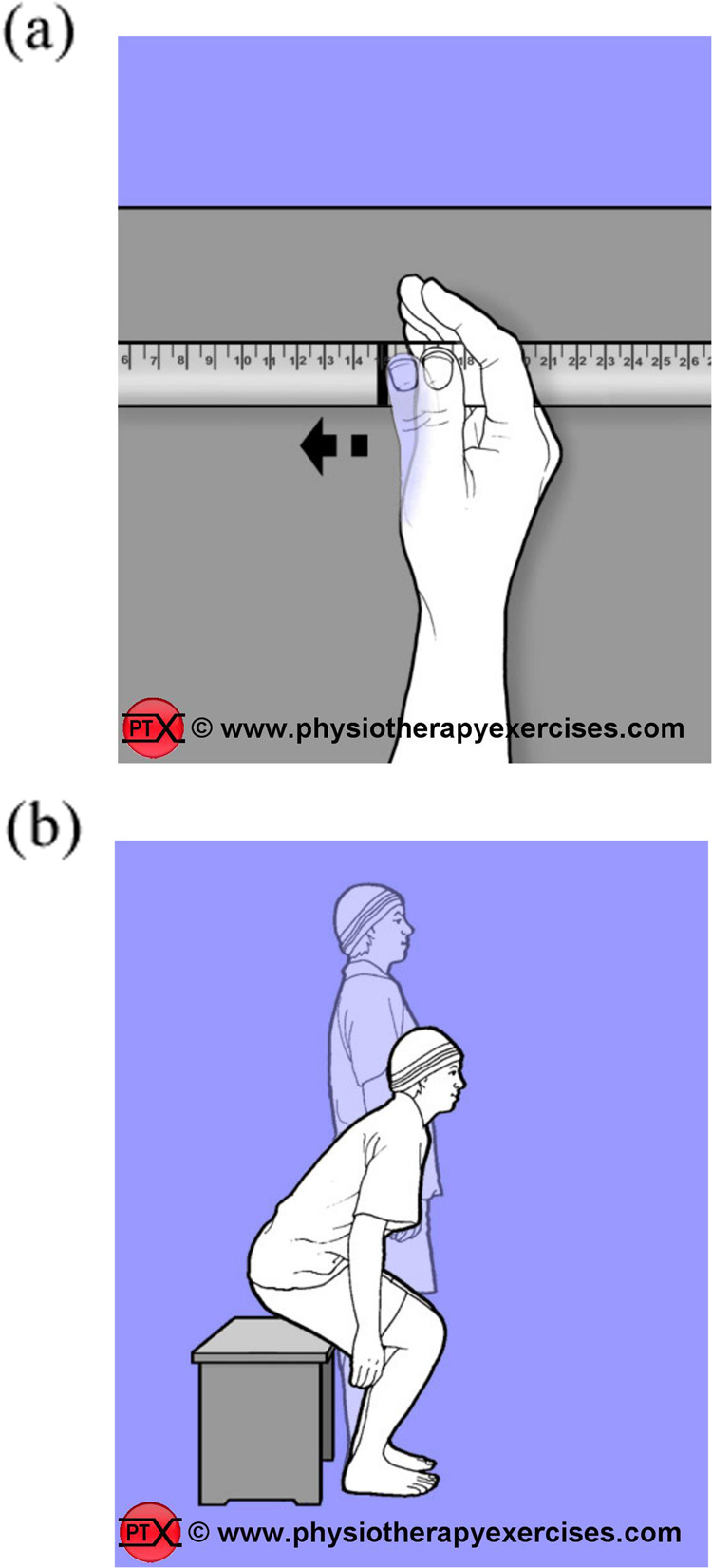
Examples of exercise set-up with target orientated instructions. **a** The distance that the thumb moves along the ruler is measured and marked by the therapist. The participant is encouraged to move the thumb to the marker every time. Number of repetitions is recorded. The participant is encouraged to move the thumb further along the ruler next time. Set-up and number of repetitions is captured in the Practice Sheet (Images copied with permission from www.physiotherapyexercises.com). **b** The chair height for a sit-to-stand exercise and the number of repetitions completed in the session are documented in the Practice Sheet. The participant is encouraged to complete more repetitions next time. The difficulty of the exercise can be increased by lowering the height of the chair, changing the foot position, or increasing the number of repetitions. These types of details are captured in the Practice Sheet.

Another important aspect of the SCI-MT Trial intervention is target orientated instructions. This helps ensure training is progressed. So, prior to commencement of any exercise the therapist and participant will together reflect on what was done in the previous session and set a new exercise target for the current session. For example, if a person could do 40 repetitions of sit-to-stand from a set height yesterday then perhaps today’s target will be to complete 44 repetitions from the same height. Alternatively, if a person can slide his or her thumb 40 mm along a ruler whilst working towards the goal of getting the hand around a cup, perhaps next time the aim will be to slide the thumb to 42mms (see Fig. 2). The setting of targets for every exercise makes it very clear to participants what they are trying to achieve. It provides motivation and it enables the therapist and participant to see improvement even if very small. It also ensures that training is progressed. However, it requires meticulous measuring and recording on Practice Sheets: an important aspect of the SCI-MT Trial.

#### Feedback

Appropriate and timely feedback is integral to skill acquisition [36]. Feedback provides the person with important information about their attempts at movement. It is critical for helping the person change future attempts at movement and is central to optimal motor learning. Feedback provided by therapists is known as augmented feedback [37]. Augmented feedback is particularly important for people with SCI because of their sensory-motor impairments. Augmented feedback can be provided in the form of knowledge of results (KR) and knowledge of performance (KP) [32, 33]. Knowledge of results refers to information about the outcomes of attempts at movement. Examples of KR feedback include information about the accuracy of stepping onto a target, the speed of walking or the number of sit-to-stand exercises completed. In contrast, KP refers to information about the quality of movement, or the kinetics and kinematics. Examples of KP include feedback about how well a person straightens his or her knee when standing or what a person does not do when trying to shift weight from one leg to another. Evidence suggests that both KR and KP are important for optimising motor learning during task-specific training [38–40]. Both types of feedback need to be provided at an appropriate time and in an appropriate amount of detail that matches the person’s stage of learning [41]. So too much feedback can be just as detrimental to learning as too little feedback. Similarly, feedback provided at the wrong time can hinder attempts at movement. Therefore, an important aspect of the Motor Training provided as part of the SCI-MT Trial will be well-timed and appropriate KR and KP feedback. The use of Practice Sheets in which the therapists and participants set and review exercise targets and goals will encourage the provision of KR feedback.

#### Goal driven

Task-specific training must be meaningful to the person. That is, it must be based on his or her own goals [42]. Setting meaningful goals can increase a person’s satisfaction with the intervention and improve motivation to engage in therapy. It can also ensure that the training is specific [43]. Motor Training based on goals that are specific and difficult is likely to produce better outcomes than training without a goal or with a non-specific easily attained goal [33]. The SMART acronym is often used to facilitate the goal-setting process, prompting therapists to set goals with patients that are Specific, Measurable, Attainable, Realistic and Timebound (there are several variations of the words associated with each letter) [44, 45].

Task-specific training as part of the SCI-MT Trial will be based on goals set by participants in collaboration with the therapists. The participants may find it difficult to set realisitic goals at these early stages of their SCI (within 10 weeks after injury), and for this reason guidance and collaboration from the therapists will be particularly important. Two ten-week and two six-month goals will be set during the baseline assessments that form part of the formal assessment schedule of the trial. These will be set using the SMART principles of goal setting. The goals will be related to participants’ activity limitations and participation restrictions and be amenable to SCI-MT intervention. Then, each week during the SCI-MT training, the therapist and participant will together identify up to four smaller goals just for the week. These weekly goals will be reviewed and progressed and will underpin the choice of task-specific exercises included in the Motor Training program.

### Strength training

Strength training is the second component of Motor Training. Whilst it is an important aspect of the SCI-MT Trial, strength training is considered supplementary, with the priority being given to task-specific training.

There is strong evidence about the effectiveness of strength training in muscles with grade 3 or more strength [46, 47]. This evidence points to the importance of progressive resistance training involving high loads and low repetitions. This typically involves 2–4 sets of 6–12 repetition maximum muscle contractions, performed 2-3 times per week, over eight weeks with progression as muscles increase in strength [46, 47]. The most effective way to strengthen very weak muscles (grade 1 and grade 2) is less clear although progressive resistance training is not a viable option because it is very difficult to operationalise (high load and low repetitions). By necessity, therefore, therapists tend to administer strength training for very weak muscles in the form of low loads and high repetitions. This type of training usually involves repeated contractions of the very weak muscle through the available joint range of motion to the point of muscle fatigue. Progress is achieved by changing the position of the limb and manipulating the effects of gravity and surface friction until the person can train against gravity, and then against resistance. The evidence about the effectiveness of this form of strength training for the very weak is inconclusive [48] but included as part of the SCI-MT intervention because it is widely administered and may be effective when combined with task-specific training.

Of course, the distinction between strength training and task-specific training for the very weak can become blurred once the focus of an exercise involves high numbers of repetitious contractions. For example, knee and hip extension exercises completed on a sliding tilt table can be considered a form of strength training but can also be a form of part-task training aimed towards improving the ability to perform sit-to-stand. For the purpose of this trial, the distinction between the two is based on whether the repetitious practice involves complex movements in the context of purposeful activity or simpler exercises involving one joint.

The key features of the strength training provided as part of the Motor Training of the SCI-MT Trial are:

#### Progression

The strength training for muscles of grade 3 and grade 4 strength will follow the principles of progressive resistance training (high load and low repetitions), whilst the strength training for muscles of grade 1 and grade 2 strength will follow the principles of low load and high repetitions training. Both types of strength training will be progressed, and the participants will be encouraged to work to fatigue in every session with the difficulty of exercises increased as required. For very weak muscles, strength training will be transitioned to higher loads with lower repetitions, as well as to task-specific training, as soon as possible.

#### Goal driven with feedback

Like task-specific training, strength training needs to be meaningful to the person and based on goals that are set at the beginning of each week. In addition, setting clear targets for each exercise, so that the participants know exactly what they are trying to achieve is an important aspect of strength training. The participants will be provided with KR feedback (e.g., information about the numbers of repetitions and the amount of load completed with each exercise) to optimise the effectiveness of the training.

## Part 2: operationalising and standardising the Motor Training provided as part of the Sci-Mt Trial

Whilst Motor Training will be individualised to the needs of each participant, it is important that the intervention is standardised within the SCI-MT Trial as far as possible, with trial therapists adhering to the key training principles. This is being achieved by articulating the key principles and guidelines of the SCI-MT in a 2 h online training package (delivered via Zoom) to all trial therapists. The training is compulsory and is provided to every SCI-MT site. The key principles of the SCI-MT Trial are summarised in Table 2.

**Table 2:** A summary of the key principles of SCI-MT intervention.

General SCI-MT principles	Task-specific training	Strength training	
		For muscles with Grade 1 and 2 strength	For muscle with Grade 3 and 4 strength
• Twelve hours of weekly therapy will be delivered in one-to-one sessions in the therapy gym for 10 weeks • The number and duration of sessions per week is not stipulated but typically the 12 h per week will be provided over 5 to 10 sessions of 1 to 2 h duration each • Therapy will target the Total Motor Score, secondary outcomes, and the participants’ goals • Practice Sheets will be used for every session • Exercise targets will be set for each exercise during each therapy session • Therapy will be progressed as much as possible • Participants will be challenged to work as hard as possible in every session • Each session will be spent with the participant actively engaged in exercise • The participants will be re-assessed regularly throughout the 10 weeks	• Focus on high numbers of repetitions • Include part-task or whole-task practice • Provide manual assistance only if essential • Feedback will be used to provide knowledge of performance and knowledge of results • Clear instructions and demonstrations will be provided • Treadmills or robotics will be used to complement training when and if appropriate	• The focus will be on high repetitions and low load • Effects of gravity and surface friction will be manipulated to enable active muscle contraction • Resistance/load will be increased as soon as possible • The strength training will be done within the context of a functional activity if possible • Manual assistance will only be provided if essential	• Training will follow the principles of progressive resistance training (ie., 3 ×10–12RM) • The strength training will be done within the context of a functional activity if possible

In addition, an Intervention Manual is provided to all trial therapists alongside the training package (see Supplementary file 1 for a copy of the intervention manual). The Intervention Manual details the training principles of the SCI-MT Trial as well as information about the use of Practice Sheets, the type of equipment that can be used, and the staff who can deliver the 12 h of Motor Training per week. Examples of Motor Training exercises are presented in the Intervention Manual and are categorised into common activities that can be targeted by Motor Training. The manual also includes completed Practice Sheets using four different hypothetical case studies. These demonstrate ways in which exercise set-up, intensity of training, and time spent on each exercise across a week can be documented.

The SCI-MT intervention will be delivered by a healthcare professional (e.g., Physiotherapist, Occupational Therapist, Exercise Physiologist) appropriately qualified to deliver all or some aspects of Motor Training once they have completed the compulsory training. The 12 h per week of Motor Training will be provided to the intervention participants on a one-to-one basis by the therapists in a therapy gym. The time allocated to Motor Training will be dedicated to as much active exercise as possible, although it will also include rest, set-up, and chat. Management of fatigue will include rests as required, with encouragement to resume exercise as soon as possible. Mental practice [49] can be used as an adjunct to training if a participant gets excessively fatigued.

The use of Practice Sheets will be at the core of delivering the SCI-MT intervention. Therapists and participants will refer to the Practice Sheets together at the beginning of each week to review previously set goals and negotiate 2–4 new goals. Therapists will also use Practice Sheets to set exercise targets, and to document exercise intensity, set-up, and duration within each session. Dosage parameters documented in the Practice Sheets will be reviewed during each session, and this will be used to encourage the participants to work as hard as they can, and to progress the exercises from day to day, and week to week.

The exercise dosage delivered during the 12 h of weekly SCI-MT Trial intervention will be documented as the *amount of time* dedicated to active practice during each therapy session. Time alone will not capture all aspects of intensity or dosage, but it will be used in the SCI-MT Trial as a crude measure of intensity that is practical to capture. This is consistent with other studies in stroke and SCI rehabilitation [2, 50, 51]. Amount of time spent on motor training during every SCI-MT session along with the type of therapy will be documented in Case Report Forms (CRF). The CRFs are based on the International Spinal Cord Injury Physical Therapy-Occupational Therapy Basic Data Set (version 1.2) [52]. The use of this Basic Data Set will enable us to capture the interventions that are delivered by the training therapists in a standardised way. Furthermore, time spent on each individual exercise (in addition to other training parameters reflecting the intensity of training) will be recorded in the Practice Sheets, which together with the CRFs, will reflect the intensity of the SCI-MT training program.

Importantly, there will be ongoing auditing of the intervention by three trial clinical experts to ensure that the intervention is being delivered as intended. The audits will involve regular reviews of the Practice Sheets at each site, with provision of feedback and ongoing training of the trial therapists as required. In addition, the fidelity of the intervention will be examined in detail with a process evaluation that will run alongside the trial. The process evaluation will also involve exploring the barriers and facilitators to the future rollout of the intervention, as well as collecting data to explain the trial results and to summarise adherence to the key principles of the intervention.

## Discussion

The SCI-MT Trial when completed will be one of the largest physiotherapy intervention trials yet to be conducted in the area of SCI. The trial addresses an important question. That is, does intensive Motor Training provided soon after injury promote neurological recovery and improve function? The results of this trial will provide the much-needed evidence for this promising intervention.

The description of the intervention is provided in as much detail as possible but one short paper can never substitute for undergraduate, postgraduate, and clinical training in the physiotherapy (and occupational therapy) management of people with SCI. There are many assumed skills that underpin the intervention. Of course, some would argue that this is a weakness of the trial. We however argue that the trial is pragmatic and needs to test the effectiveness of an intervention as it would typically be administered in the clinical setting [53]. It, therefore, needs to allow for a clinician’s clinical reasoning skills. It also needs to accommodate the many different ways people are affected by SCI and their individual goals and problems. Nonetheless, great effort is being directed at ensuring that those administering the intervention are appropriately skilled and trained.

There is a risk that people with recent SCI will not tolerate 12 h of additional therapy each week so soon after injury, or they will not receive this amount of therapy for other unforeseen reasons. These concerns were not explored in a feasibility study per se as the intervention was based on current practice and includes components that have been examined in similar previous studies [46–48]. Deviations from study protocol will all be closely recorded and monitored. All participants will be encouraged, but not obliged, to attend therapy and some participants will no doubt opt out. Issues like this will not nullify the results or be fatal flaws because the trial is pragmatic, not explanatory [53]. It is asking about the outcomes if we set in place staffing, policies and structures so that the spinal units can provide the maximal amount of therapy that is conceivably possible. The participants’ abilities and willingness to receive this additional therapy are all part of the construct that is being tested. It is part of the real-world scenario.

In all, this paper provides the details of the Motor Training provided as part of the SCI-MT Trial. The details will increase transparency and help interpret the future results of the trial. This paper may also help those not trained in physiotherapy to appreciate some of the nuances of providing task-specific training to people with SCI. And while repetitious practice of purposeful movements is central to task-specific training, there are many other important principles that must also be followed.

### Supplementary information

SCI-MT Trial Intervention Manual

Reproducibility checklist
