# Supplementary material for: Early and intensive Motor Training for people with spinal cord injuries (the SCI-MT Trial): description of the intervention
Source: Spinal Cord. 2023 Jul 19;61(11):600–7. doi: 10.1038/s41393-023-00911-4 (PMC10645584; doi:10.1038/s41393-023-00911-4)
Supplement: Supplementary file 1 — SCI-MT Trial Intervention Manual [file 41393_2023_911_MOESM1_ESM.pdf]

Early and intensive motor training (versus usual care) to enhance neurological recovery and function in people with spinal cord injury (SCI)

The Early & Intensive SCI-MT Trial

The Intervention Manual 1.1, 5th March 2021

Clinical Trial Sponsor: The University of Sydney

Clinical Trial funder: NSW Ministry of Health

Clinical Trial Registration no: 380277

Universal Trial no: U1111-1264-1689

STATEMENT OF COMPLIANCE

This clinical research study (clinical trial) will be conducted according to the protocol as approved by the responsible human research ethics committee (HREC), and in compliance with all approval conditions of the approving HREC, institutional governance authorities, the NHMRC National Statement on Ethical Conduct in Human Research 2007 (updated 2018),[1] and the Guideline for Good Clinical (Research) Practice (GCP) ICH E6 (R2).[2]

TABLE OF CONTENT

1 PURPOSE OF THIS MANUAL 4

Note on all images used in this manual 4

2 THE ADDITIONAL MOTOR TRAINING TO BE PROVIDED TO INTERVENTION PARTICIPANTS 4

What, When, How and Why 4

2.1 What type of therapy can be provided as part of the Additional Motor Training for Intervention Participants? 4
2.2 What is the aim of the Additional Motor Training? 4
2.3 When and how much Additional Motor Training is to be provided? 4
2.4 Do all participants in the Intervention Group receive the same package of Additional Motor Training? 5
2.5 What is the mode of delivery of the Additional Motor Training for the Intervention Participants? 5

Key principles and considerations of Additional Motor Training 5

2.6 What are some of the key principles for all sessions provided as part of the Additional Motor Training? 5
2.7 What are some of the considerations for all sessions provided as part of the Additional Motor Training? 5
2.8 What types of therapies and equipment can be used? 5
2.9 Do all participants need to participate in gait training with a treadmill? 6
2.10 Do all participants need to receive electrical stimulation? 6

Considerations for implementing the Additional Motor Training 6

2.11 When does the Additional Motor Training commence? 6
2.12 Does 12 hours include or exclude time for rest, setup and chat? 6
2.13 Who can provide the Additional Motor Training? 6
2.14 Can the same therapist provide the Additional Motor Training and Usual Care for an Intervention Participant? 6
2.15 Can different staff provide different sessions of the Additional Motor Training to the same Intervention Participant? 7
2.16 Can students or aides provide the Additional Motor Training? 7
2.17 What if the staff member responsible for providing the Additional Motor Training is sick? 7

Recording Sheets and CRF Forms 8

2.26 What is a Practice Sheet? 8
2.27 What is the difference between a Practice Sheet and the "Case Report Forms (CRF) for Usual Care and Additional Motor Training"? 8
2.28 When should the "Case Report Forms (CRF) for Usual Care and Additional Motor Training" be completed? 8
2.29 How are the therapies to be coded as part of the "CRF for Usual Care and Additional Motor Training"? 8

Goal setting 9

2.30 What are the baseline goals? 9
2.31 Does the therapist providing the Additional Motor Training need to record whether and when the baseline goals are achieved? 9
2.32 Does the therapist providing the Additional Motor Training need to set weekly therapy goals (in additional to those set as part of the baseline assessment)? 9
2.33 Does the therapist providing the Additional Motor Training need to record whether the weekly therapy goals are met? 9
2.34 Do new therapy goals need to be set every week? 9

Key principles of motor training 10

Key principles for strength training ... *11*

3 MOTOR TRAINING EXERCISES ... 12

3.1 Motor training exercises to improve arm and hand function ... 13
3.2 Motor training exercises to improve the ability to move about on a bed ... 18
3.3 Motor training exercises to improve the ability to sit ... 19
3.4 Motor training exercises to improve the ability to stand ... 21
3.5 Motor training exercises to improve the ability to step and walk ... 24
3.6 Motor training exercises to improve the ability to move from sit to stand ... 28
3.7 Motor training exercises to improve the ability to ascend/descend steps ... 30
3.8 Motor training exercises to improve advanced gait related activities ... 31

4 STRENGTH TRAINING EXERCISES ... 33

4.1 Strength training exercises for upper limb muscles (grade 1 and 2 strength) ... 34
Shoulder muscles (grade 1 and 2) ... 34
Elbow muscles (grade 1 and 2) ... 35
Wrist and hand muscles (grade 1 and 2) ... 36
4.2 Strength training exercises for upper limb muscles (grade 3 and 4 strength) ... 37
Shoulder muscles (grade 3 and 4) ... 37
Elbow muscles (grade 3 and 4) ... 43
Wrist and hand muscles (grade 3 and 4) ... 44
4.3 Strength training exercises for lower limb muscles (grade 1 and 2 strength) ... 46
Hip muscles (grade 1 and 2) ... 46
Knee muscles (grade 1 and 2) ... 47
Ankle muscles (grade 1 and 2) ... 48
4.4 Strength training exercises for lower limb muscles (grade 3 and 4 strength) ... 49
Hip muscles (grade 3 and 4) ... 49
Knee muscles (grade 3 and 4) ... 53
Ankle muscles (grade 3 and 4) ... 55

5 EXAMPLES OF 4 CASE STUDIES TO ILLUSTRATE THE ADDITIONAL MOTOR TRAINING THAT COULD BE PROVIDED IN A TYPICAL WEEK ... 58

5.1 Case 1: Person with a C5 neurological level and AIS C lesion ... 60
5.2 Case 2: Person with a C5 neurological level and AIS D lesion ... 66
5.3 Case 3: Person with a T10 neurological level and an AIS C lesion ... 74
5.4 Case 4: Person with a T10 neurological level and an AIS D lesion ... 79

6 PRACTICE SHEETS FOR INTERVENTION PARTICIPANTS ONLY ... 84

6.1 Purpose: ... 84
6.2 Instructions for therapists: ... 84

7 APPENDICES ... 91

7.1 Lists of treatments and therapies that are considered appropriate and inappropriate ... 91

8 REFERENCES ... 92

1 PURPOSE OF THIS MANUAL

The purpose of this intervention manual is to guide the delivery of the Additional Motor Training provided to Intervention Participants as part of the SCI-MT Trial. The aim of this manual is to provide ideas and support to the training therapists delivering the intervention. It is recommended this manual be used as a guide together with the therapists' own clinical knowledge and reasoning.

Note on all images used in this manual

- All images are copied with permission from www.physiotherapyexercises.com; freely available exercise-prescribing software designed for, and by, physiotherapists for people with injuries and disabilities.
- The images reflect physical presentations of people with different types of Neurological Impairments, and are not always representative of people with SCI. They are only intended to convey the principles of an exercise, and to be used as cues and prompts. They may need to be modified to the needs of each person.
- The titles of the exercises are the same as used in www.physiotherapyexercises.com but do not always fully convey the intention of the exercise.

Further details about each exercise can be found on www.physiotherapyexercises.com including suggestions on how to make the exercise easier or harder. Use the title of each exercise as "key words" to search. Then, right click on the matching image to see the full details.

2 THE ADDITIONAL MOTOR TRAINING TO BE PROVIDED TO INTERVENTION PARTICIPANTS

What, When, How and Why

2.1 **What type of therapy can be provided as part of the Additional Motor Training for Intervention Participants**?

The type of therapy to be provided as part of the Additional Motor Training for Intervention Participants is any therapy that involves motor training of muscles at and below the level of the injury. The therapy needs to involve active contractions of neurologically weak muscles and can be broadly defined as:

- Motor skill training, and
- Strength training.

2.2 What is the aim of the Additional Motor Training?

The aim of the Additional Motor Training is to improve the Total Moto Score (primary outcome) with the overall aim of improving function and secondary outcomes as well as addressing participants' baseline goals. For this reason, any therapy that involves active contraction of muscles at and below the level of the injury that could conceivably increase the Total Motor Score, improve secondary outcomes and address participants' goals is a priority.

2.3 When and how much Additional Motor Training is to be provided?

Participants in the Intervention Group need to receive 12 hours of Additional Motor Training per week for 10 weeks as well as their Usual Care. The Additional Motor Training can be provided as 2 hours per day from Monday to Saturday, or in larger blocks of therapy on Saturdays and Sundays, or any combination of both.

2.4 Do all participants in the Intervention Group receive the same package of Additional Motor Training?

No, the Additional Motor Training needs to be individualised to the needs of each participant. It is important that it adheres to the principles of the SCI-MT Trial but does not need to be the same for all.

2.5 What is the mode of delivery of the Additional Motor Training for the Intervention Participants?

The Additional Motor Training is to be provided through face-to-face sessions with a therapist (or appropriately qualified/supervised person). This does not include any exercise or therapy conducted without direct supervision or as part of a group class.

Key principles and considerations of Additional Motor Training

2.6 What are some of the key principles for all sessions provided as part of the Additional Motor Training?

The key principles are:

- Ensure that the therapy targets the Total Motor Score, secondary outcomes and the participant's goals that were set as part of the baseline assessment.
- Keep Practice Sheets for participants to capture their progress (e.g. captures the number of repetitions of an exercise or the difficulty of an exercise). You can use http://www.physiotherapyexercises.com to quickly generate these or make your own.
- Set targets for each exercise during each therapy session (see page 48).
- Progress all therapy sessions as able.
- Ensure participants work hard in every session.
- Use manual assistance/guidance if needed but remove it as soon as possible.
- Spend as much time as possible of each session with the participant **actively** engaged in exercise.
- Vary treatments to increase participant adherence.
- Ensure the participant is appropriately assessed at the start and regularly re-assessed throughout the 10 weeks to help formulate an appropriate therapy program and to monitor progress (assessment time is part of Additional Motor Training time).

2.7 What are some of the considerations for all sessions provided as part of the Additional Motor Training?

The main considerations are:

- Prepare your treatment plan prior to each session.
- Prepare equipment needed prior to each session.
- Arrange extra staff to be available prior to the session (e.g., when 2 therapists/assistants are needed for gait training).
- Plan for sessions that requires participants in versus out of their wheelchairs to maximise Additional Motor Training time. Aim to be systematic in your approach and minimise the need for changing participants' position e.g.: perform all supine exercises then move to sidelying, sitting as intervention plan dictates.

2.8 What types of therapies and equipment can be used?

It is appropriate to use any of the following therapies or equipment provided they require the participant to actively contract neurologically weak muscles at and below the level of the injury or they supplement/reinforce the participant's efforts:

- Biofeedback

- Electrical stimulation including functional electrical stimulation cycling
- Mental imagery
- Somatosensory stimulation
- Robotics
- Computer game-based therapy
- FES lower limb or upper limb cycling
- Exoskeleton

See Section 7 (page 78) for a comprehensive list of treatments and therapies that are considered appropriate and inappropriate.

2.9 Do all participants need to participate in gait training with a treadmill?
No, this is not essential however try to incorporate gait training with a treadmill when and if appropriate.

2.10 Do all participants need to receive electrical stimulation?
No, this is not essential however try to incorporate electrical stimulation when and if appropriate.

Considerations for implementing the Additional Motor Training

2.11 When does the Additional Motor Training commence?
The Additional Motor Training needs to commence as soon as possible after randomisation and preferably the next day. It is therefore important to ensure that this is possible before randomising a participant. Importantly, the clock starts ticking from the day of randomisation. So it is not possible to delay the start of the Additional Motor Training after randomisation with the hope of counting 10 weeks from this time.

2.12 Does 12 hours include or exclude time for rest, setup and chat?
The 12 hours includes time within a session devoted to rest, setup and chat, however, as far as possible ensure that the participants spend as much time as possible actively exercising.

2.13 Who can provide the Additional Motor Training?
Any appropriately qualified healthcare professional (e.g. Physiotherapist, Occupational Therapist, Exercise Physiologist) can provide the Additional Motor Training. Alternatively, different combinations of healthcare professionals can provide different aspects of the participant’s Additional Motor Training. However, the therapy provided must be a comprehensive package of motor skill training and strength training to address all muscles at and below the level of the injury. It is not acceptable to just focus on one aspect. For example, it is not appropriate for a person with incomplete tetraplegia to only receive motor skill training to improve hand function from an Occupational Therapist if the person would also benefit from a strengthening program for the upper limbs. Similarly, it is not appropriate for a person with incomplete paraplegia to only receive strengthening exercises for the lower limbs from an Exercise Physiologist without motor skill training. For this reason, we anticipate that most therapy will be provided by a Physiotherapist because he/she can typically administer all aspects of the training.

2.14 Can the same therapist provide the Additional Motor Training and Usual Care for an Intervention Participant?
Yes, but we don’t recommend this because there is a high risk that therapists will modify their Usual Care in response to the trial. So we do not want Usual Care to solely consist of all the

therapies not provided as part of the Additional Motor Training sessions. That is, we want Usual Care to include the motor training that participants would have received if not in the trial and to mirror the Usual Care provided to Control participants.

2.15 Can different staff provide different sessions of the Additional Motor Training to the same Intervention Participant?

Yes, different staff can provide different sessions for the same participant provided there is appropriate handover and continuity. Practice Sheets will help ensure this and are recommended to assist with the progression of therapy. These need to be stored in the Participants' Trial Folder and each sheet needs to have the participant's unique trial code.

2.16 Can students or aides provide the Additional Motor Training?

Yes, but they must be closely supervised. It is not acceptable for the Additional Motor Training to be solely provided by students or aides.

2.17 What if the staff member responsible for providing the Additional Motor Training is sick?

Try as far as possible to have a contingency plan for when staff are away or sick. The odd day is unavoidable but many days will be very problematic and could compromise the trial.

2.18 Does the Additional Motor Training get provided every day or just on the weekend?

The Additional Motor Training can be provided in any way that suits the participant and the usual care team. So it can be provided as six 2-hour sessions over 6 days or on the weekend in longer blocks of time or in any combination of both. Anything is acceptable provide the total amount of therapy is 12 hours per week.

2.19 Can more than one session of Additional Motor Training be provided per day?

Yes, the Additional Motor Training can be provided in any number of sessions. So, it would be acceptable to provide a one-hour session in the morning and a one-hour session in the afternoon. This arrangement would be optimal for participants that are likely to suffer fatigue but may be difficult to organise.

2.20 What if a participant gets tired during a session?

Provide participants with rest as required but encourage them to resume as soon as is possible. Encourage mental practice as a substitute for physical exercise if a participant gets tired and as appropriate.

2.21 What if a participant can't finish a planned session because of fatigue?

If a participant in unable to complete a planned session then consider trying to provide multiple short sessions in the future.

2.22 Can missed Additional Motor Training sessions be made up?

Try as far as possible to make up for missed sessions during the following week or on weekends as tolerated. If a participant misses a couple of weeks of Additional Motor Training because of illness then try as far as possible to make up additional sessions once the participant is well again. However, do not try to meet the overall target if the participant can't tolerate it.

2.23 Does the Additional Motor Training need to be provided in the gym?

No, the Additional Motor Training does not always need to be provided in the gym. It could be provided in a participant's room and could be provided to a participant in bed. However, clearly it would be better if the Additional Motor Training is provided in the gym because the gym has

appropriate equipment and facilities. So try to minimise the amount of therapy provided outside the gym.

2.24 Can participants do independent therapy by themselves or with family members outside of the trial?

Yes, participants can do independent therapy by themselves or with family members outside of their usual therapy sessions as long as it is within the normal standards of usual care.

2.25 What if a participant is moved from one hospital to another?

If a participant is moved from one hospital to another (e.g. RNSH to RR) then ensure that there is an appropriate handover. Also ensure that the second hospital is provided with plenty of notice so they can organise the staff required to provide the Additional Motor Training. The Site Principal Investigator should take responsibility for ensuring this occurs.

Recording Sheets and CRF Forms

2.26 What is a Practice Sheet?

A Practice Sheet is where the participant's weekly therapy goals and the details about each exercise that they perform are recorded (see page 71). Practice Sheets are recommended as part of best practice and are useful for handover between different therapists and sites. The Practice Sheets need to be kept in the Participant's Trial Folder but they are not sent to George Clinical.

2.27 What is the difference between a Practice Sheet and the "Case Report Forms (CRF) for Usual Care and Additional Motor Training"?

Practice Sheets are to be used to record the Additional Motor Training provided and to ensure therapy adheres to the principles of best practice. In contrast, the Case Report Forms are an essential part of the trial. The data from the CRFs are critical and will be collated to summarise the Additional Motor Training and Usual Care.

2.28 When should the "Case Report Forms (CRF) for Usual Care and Additional Motor Training" be completed?

A participant's CRF needs to be completed during /at the end of each Usual Care and Additional Motor Training session (and once completed they need to be stored in the participant's trial folder). At the end of the week all CRFs need to be scanned and sent to George Clinical for data collection. This is the responsibility of the Site Principal Investigator. New CRFs should be commenced at the beginning of each week. There is no limit to how many CRFs can be used in one week but it is essential that the therapy provided as part of Usual Care is not captured on the same page used to capture the Additional Motor Training. That is, the Intervention Participants will have at least two sets of CRFs; one set to document Usual Care and a second set to document the Additional Motor Training.

2.29 How are the therapies to be coded as part of the "CRF for Usual Care and Additional Motor Training"?

Both Usual Care and Additional Motor Training are to be described using the coding system on the CRF. If an intervention could be classified in more than one way, just pick one of the most appropriate categories giving priority to one of the top 5 interventions on the list (i.e., activity directed interventions) or split the total time between the various appropriate categories.

Goal setting

2.30 What are the baseline goals?

Four goals will be set as part of the baseline assessment by the assessor and usual care team in conjunction with the participant; 2 short-term and 2 long-term goals to be achieved at 10 weeks and 6 months post randomisation, respectively. The baseline goals are to be used when devising participant's individualised Additional Motor Training program and weekly goals.

2.31 Does the therapist providing the Additional Motor Training need to record whether and when the baseline goals are achieved?

No, this will be done as part of the formal 10-week and 6-month assessment. However, the therapist providing the Additional Motor Training and the participant should know these goals and therapy should be directed at achieving them along with improving secondary outcomes.

2.32 Does the therapist providing the Additional Motor Training need to set weekly therapy goals (in additional to those set as part of the baseline assessment)?

Yes, at the beginning of each week the therapist providing the Additional Motor Training and participant should negotiate 2-4 goals directly related to the baseline goals for the week or to improving Total Motor Scores or any of the secondary outcomes. These goals should be documented at the top of the participant's Practice Sheet.

2.33 Does the therapist providing the Additional Motor Training need to record whether the weekly therapy goals are met?

No, achievement of weekly therapy goals do not need to be formally recorded. However, the therapist providing the Additional Motor Training should reflect (and informally assess) whether these are achieved at the end of each week with the participant. The therapist can put a large tick through a goal at the end of a week if it is achieved. Participants needs to finish each week with a very clear understanding of whether they did or did not achieve their weekly therapy goals.

2.34 Do new therapy goals need to be set every week?

Not always. So occasionally a goal from the previous week could be carried across to the next week. However, try to write goals that are achievable within a week even if the goal is very small and specific. It is fine to change a goal only slightly from week to week (for example, completing a task in 15 seconds one week and completing a task in 10 seconds the next week). If one of your goals takes 2 or more weeks to achieve then it is probably too difficult and needs to be modified.

Key principles of motor training

The key principles of motor training vary according to participants' initial strength (see Table 1 for summary). However, irrespective of strength, all motor training should:

- Focus on a high number of repetitions
- Do part or full practice of tasks
- Provide manual assistance only if essential
- Use all types of feedback to provide knowledge of performance and knowledge of results
- Provide clear instructions and demonstrations
- Use treadmill or similar technology for gait training when and if appropriate

Table 1: The key principles of motor training

Grade 1 - 2	Grade 3 - 4
• High repetitions of part practice of specified task.<br>• Task related training.<br>• Progress to whole task when able.	• Part and whole task practice.<br>• Increase physical demand of task when able.

Key principles for strength training

The key principles of strength training vary according to participants' initial strength (see Table 2 for summary).

Very weak muscles (Grade 1-2):

- Focus on a high number of repetitions
- Add resistance/load as soon as possible
- Train within the context of a functional activity if possible
- Provide manual assistance if essential

Stronger muscles (Grade 3-4):

- Follow the principles of progressive resistance training (ie., 3 x 10-12RM)
- Train within the context of a functional activity if possible

Table 2: The key principles of strength training

Grade 1 -2	Grade 3 - 4
• High repetitions in a gravity eliminated position.<br>• Provide assistance as required.<br>• Consider training in mid joint range, and training eccentrically or isometrically.<br>• Supplement voluntary effort with:<br>o Mental practice<br>o Electrical stimulation<br>o Biofeedback<br>• Add resistance when able.<br>• Progress to against gravity position in reduced range.	• Progressive resistance training as per strength training principles.<br>• Progress weight / resistance weekly aiming for 10-12 RM.<br>• Train to fatigue.

3 MOTOR TRAINING EXERCISES

These are intended as a guide only to illustrate some of the different types of motor training exercises that could be used to improve function and mobility as part of the SCI-MT Trial. The exercises can be varied according to a participant's strength and ability. Some of the exercises could be categorised/administered as strength training exercises provided they are administered using the principles of strength training. The exercises would only be appropriate if they targeted muscles at or below the level of the injury and could conceivably increase Total Motor Scores or any of the secondary outcomes.

Note on all images used in this manual

- All images are copied with permission from www.physiotherapyexercises.com; freely available exercise-prescribing software designed for, and by, physiotherapists for people with injuries and disabilities.
- The images reflect physical presentations of people with different types of Neurological Impairments, and are not always representative of people with SCI. They are only intended to convey the principles of an exercise, and to be used as cues and prompts. They may need to be modified to the needs of each person.
- The titles of the exercises are the same as used in www.physiotherapyexercises.com but do not always fully convey the intention of the exercise.

Further details about each exercise can be found on www.physiotherapyexercises.com including suggestions on how to make the exercise easier or harder. Use the title of each exercise as "key words" to search. Then, right click on the matching image to see the full details.

3.1 Motor training exercises to improve arm and hand function

Reaching diagonally in sitting	Taking a cup to the mouth	Reaching from a low surface to a high surface	Reaching to different targets
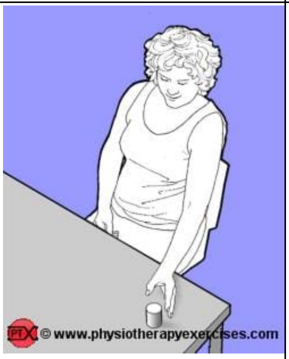	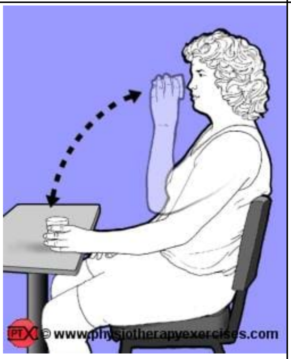	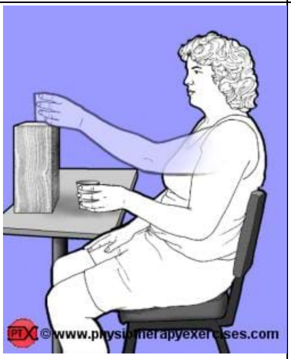	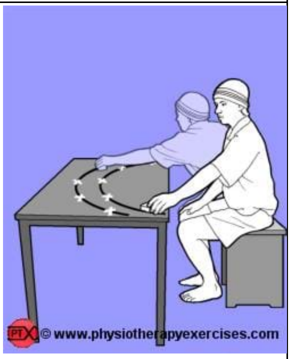

Pouring water between cups	Pouring beans from a cup	Lowering a cup of water	Lifting a cup of water
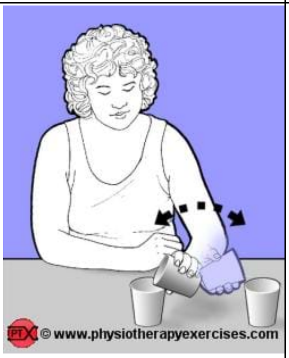	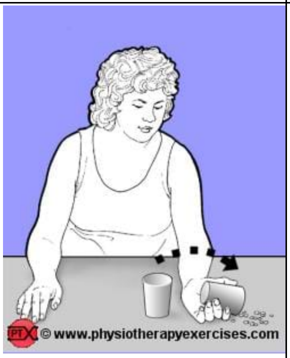	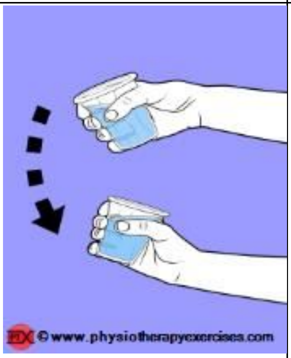	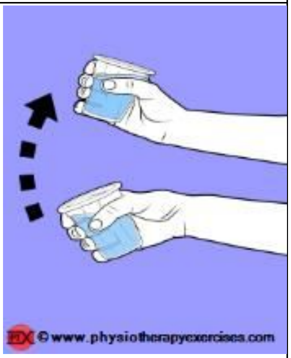

Reaching forward to an object	Metacarpophalangeal joint extension using extensor digitorum communis	Using an eggbeater	Thumb abduction using a tennis ball
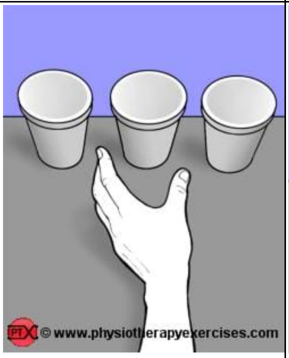	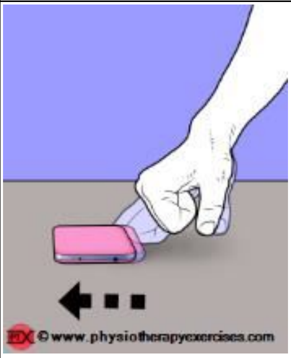	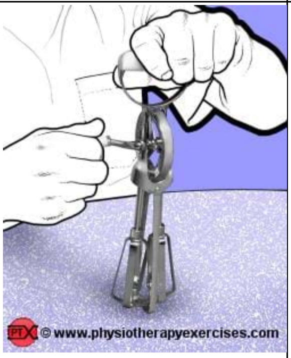	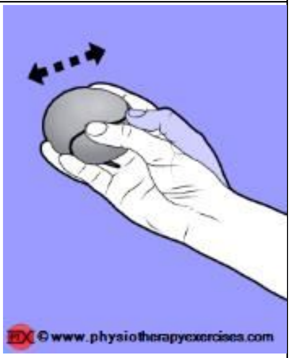

Two point pinch	Maintaining grasp using tweezers and a coin	Depressing a fork whilst holding onto tweezers and a coin	Depressing a fork using the fingertips
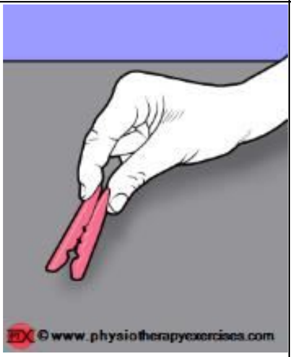	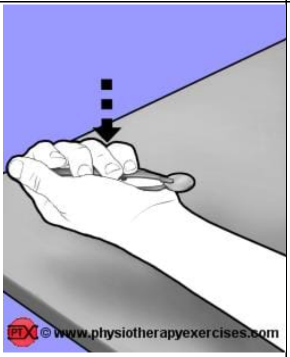	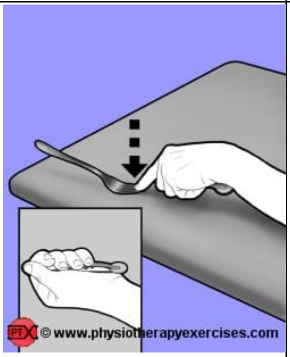	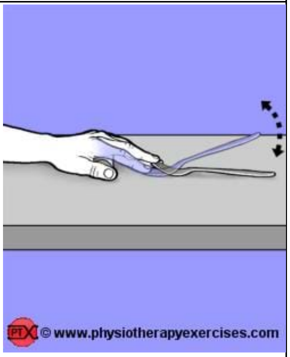

Tearing paper	Moving water between two containers	Putting on a glove	Using a pen
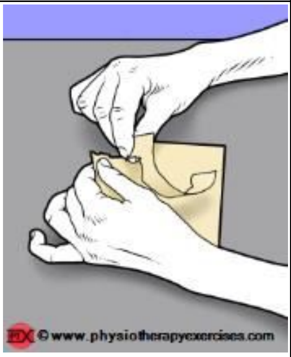	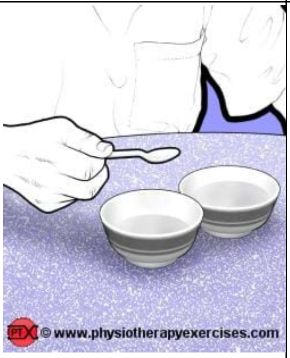	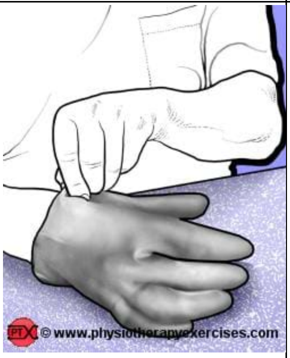	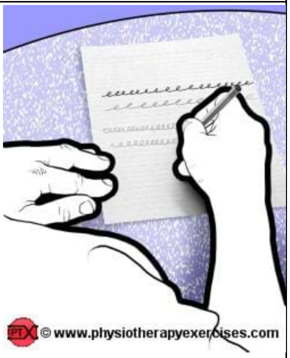

Using an adjustable spanner	Using scissors	Opening a combination lock	Opening a leash clip
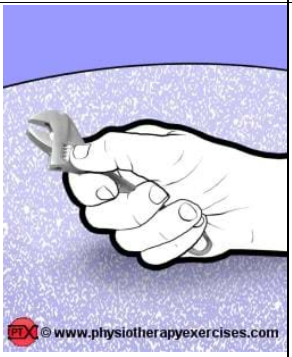	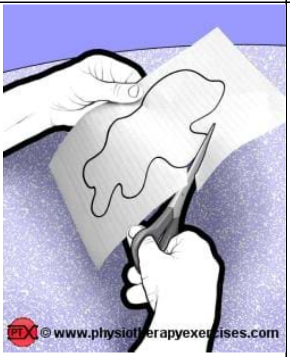	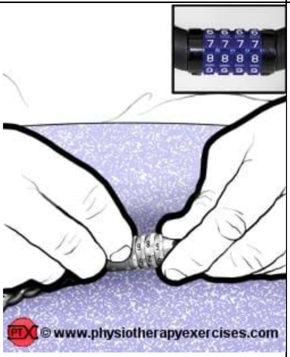	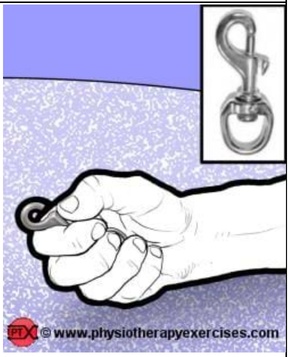

Picking up an object between the thumb and fingertips	Controlling finger flexor force by lowering peg from horizontal to vertical	Rhythmical finger tapping	Opening a container
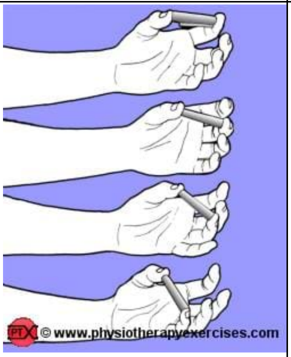	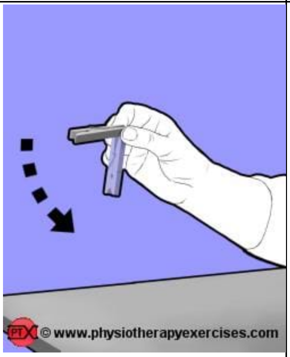	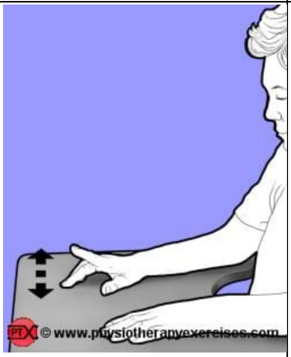	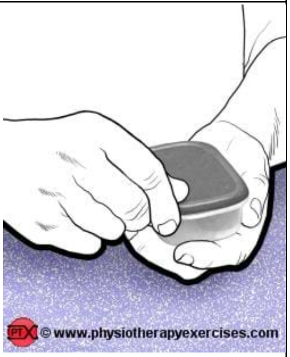

Pushing down with a fork	Using a knife and fork	Cutting with a knife	Maintaining grasp of a fork using tweezers and a coin
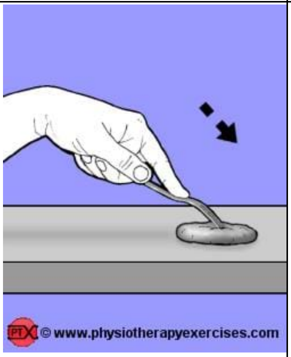	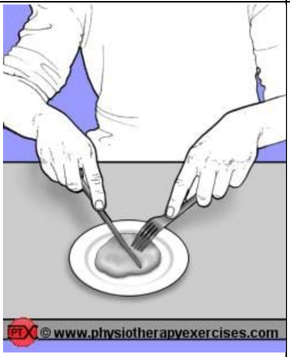	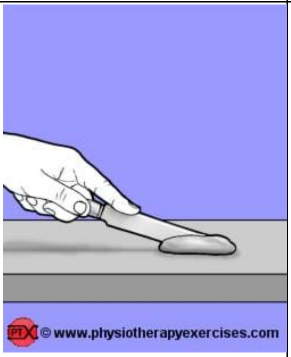	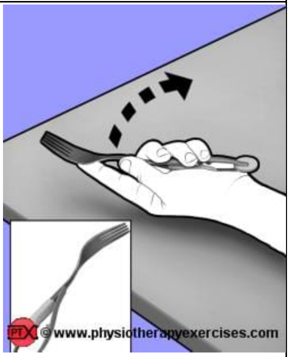

Shifting a pen up and down within the hand (Caterpillar)	Connecting a plug and socket	Rolling a pen within the hand	Using a safety pin
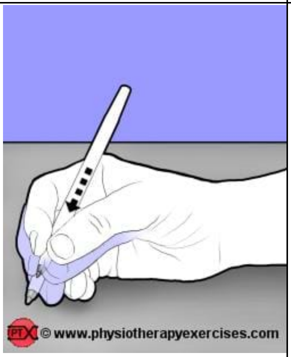	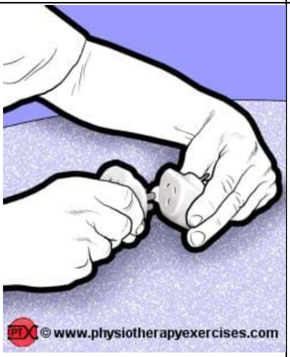	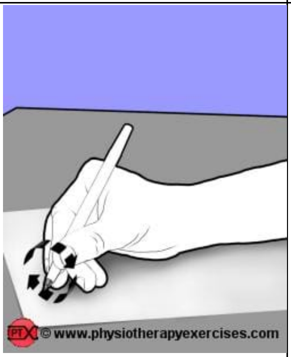	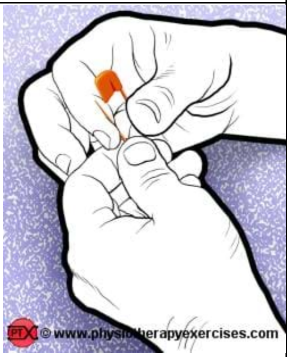

Moving objects between two containers	Turning nuts and bolts	Picking up a coin	Moving a fork sideways to targets using the fingertips
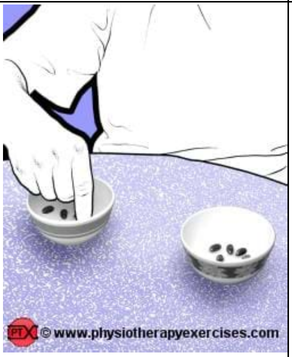	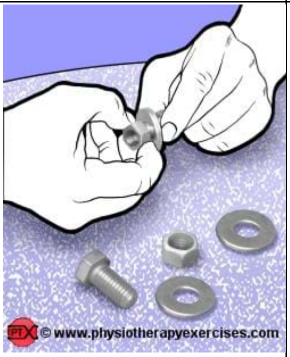	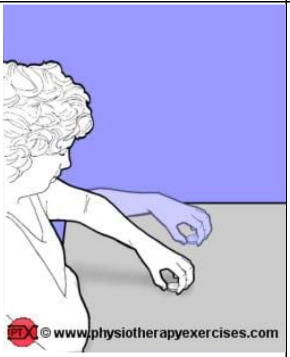	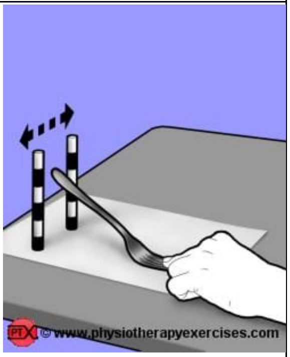

Placing pegs in a peg board	Picking up coins and placing them in a jar	Pouring water from one cup to another	Rolling a ball between the thumb and fingertips
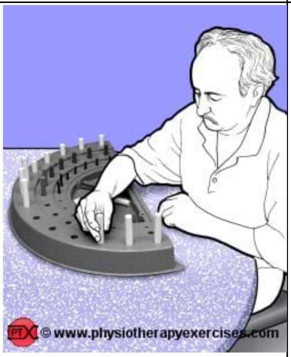	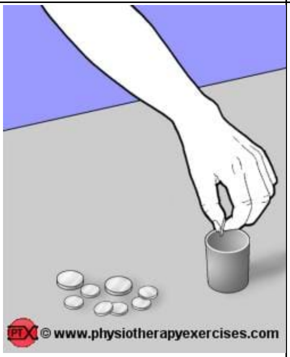	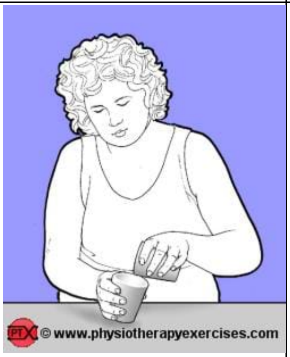	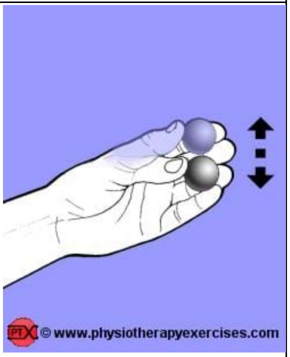

Rotating a powerball	Using tweezers	Using pegs	Pressing numbers on a phone
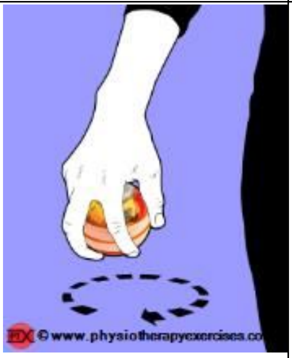	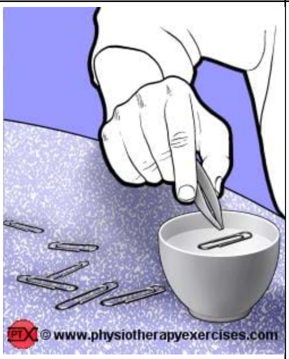	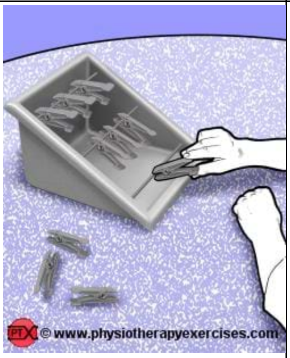	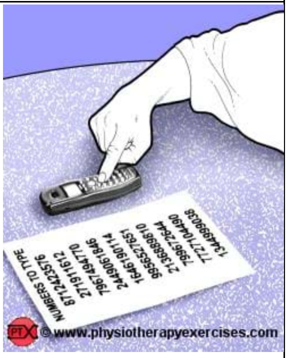

Pouring beans into a cup	Pouring water into a cupped palm	Rolling two balls around in the hand with the thumb	Using a tape measure
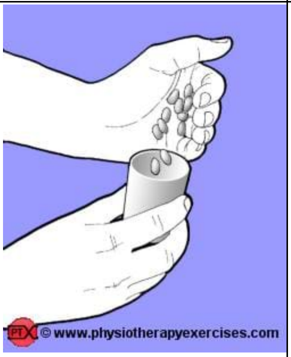	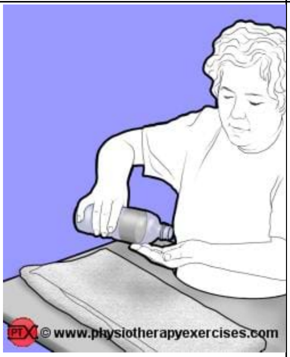	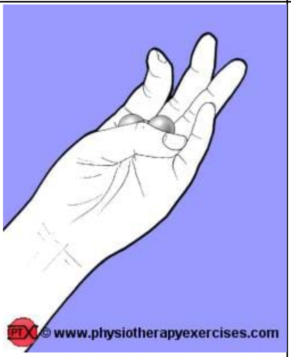	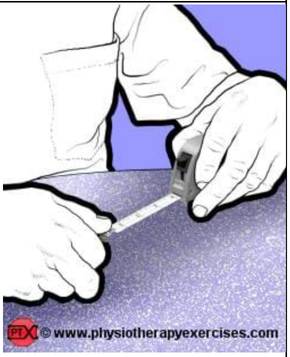

Pinch and pull putty	Wringing putty	Wringing a towel in supination	Wringing a towel in pronation
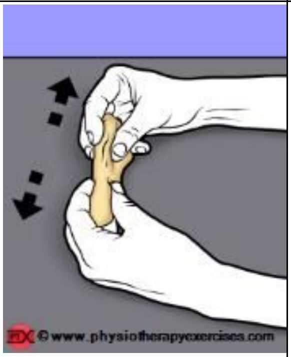	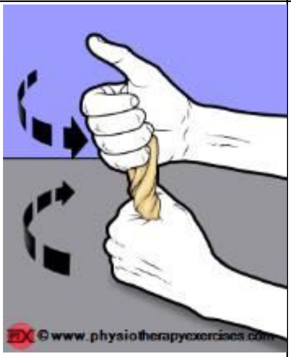	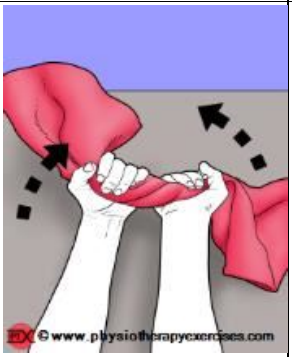	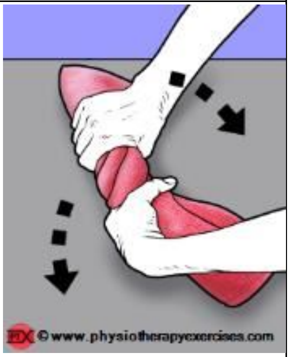

Picking up and holding onto coins	Popping bubble wrap between the fingers	Holding paper between fingers	Threading macaroni
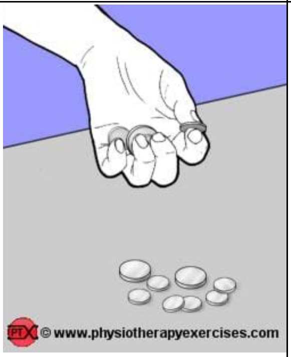	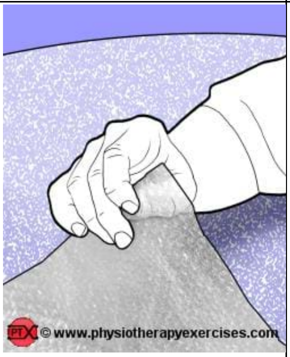	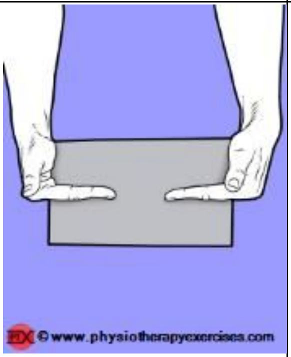	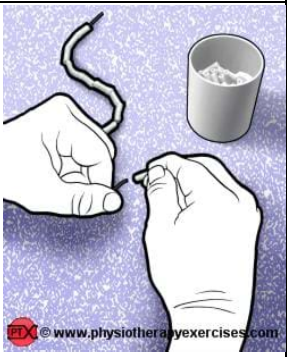

3.2 Motor training exercises to improve the ability to move about on a bed

Lifting the head sideways	Rolling over	Rolling over on the spot	Sitting up from sidelying
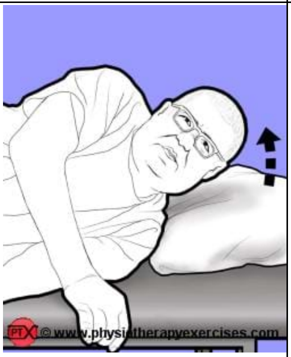	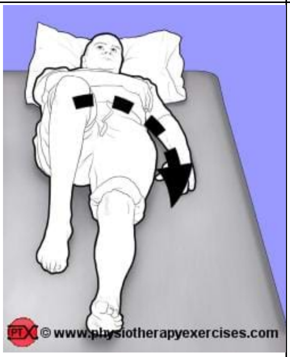	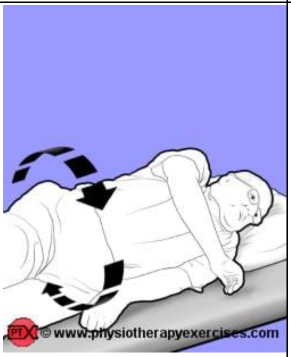	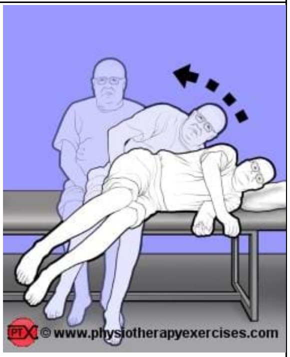

Sitting up from supine	Moving across the bed
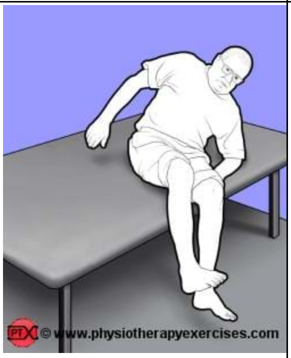	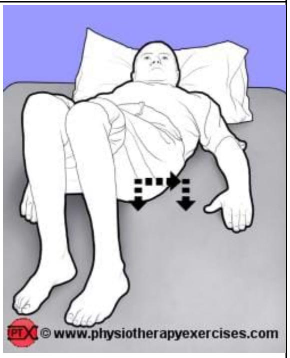

3.3 Motor training exercises to improve the ability to sit

Sitting up straight	Sitting and turning around	Turning the head and shoulders in sitting	Moving from side to side in sitting
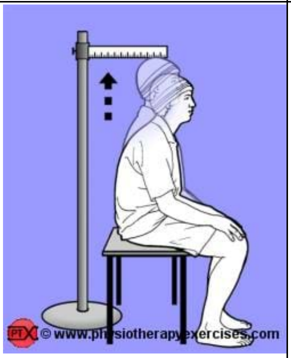	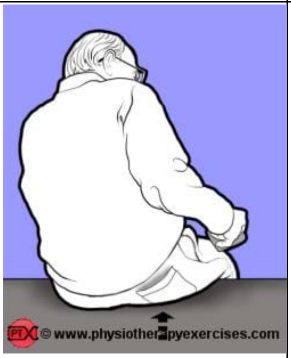	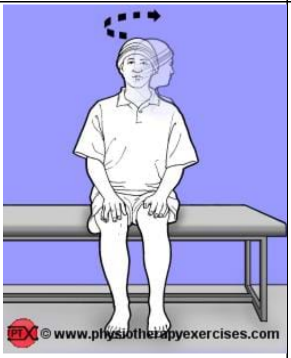	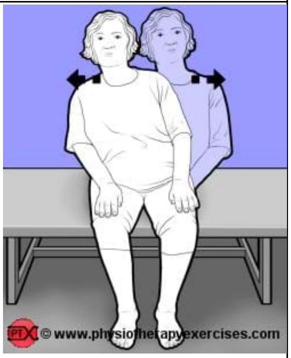

Looking up and down in sitting	Reaching while seated	Reaching from side to side in sitting	Sitting and reaching behind
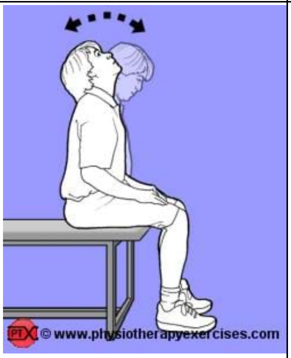	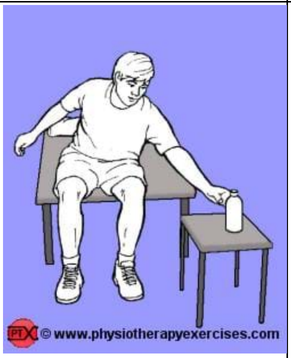	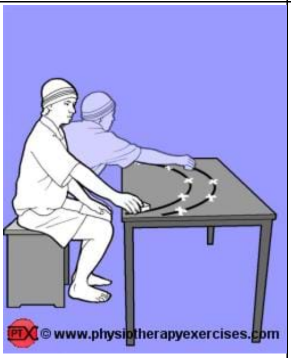	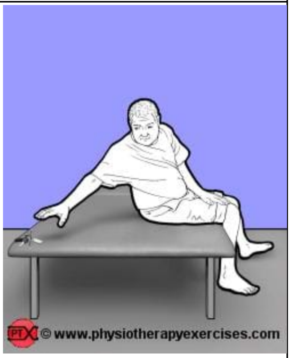

Moving the shoulders forwards in sitting to a target	Moving the shoulders forwards in sitting using a moving table	Picking an object off the floor	Lifting an object from the floor to the table
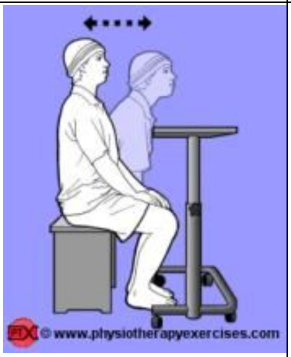	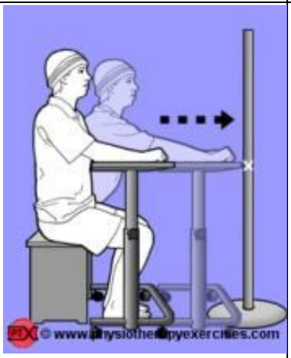	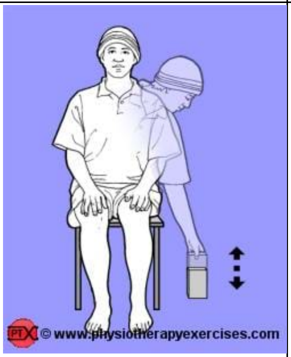	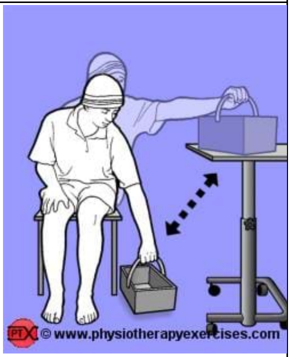

Flexing the hips in sitting and sliding arms forward on a table to a target	Reaching forward in sitting	Sitting on a high plinth and weight-bearing through the affected leg	Sitting and weight-bearing through the affected leg
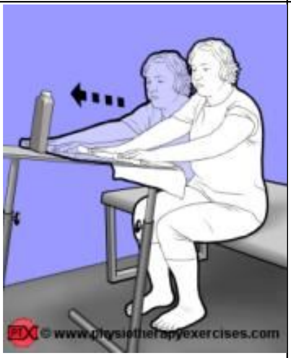	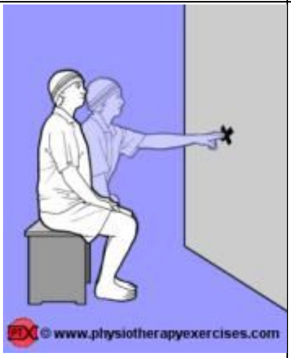	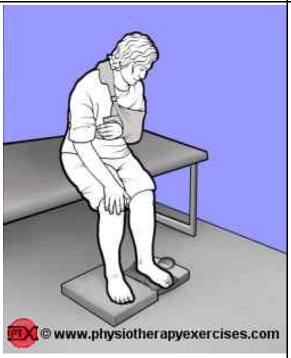	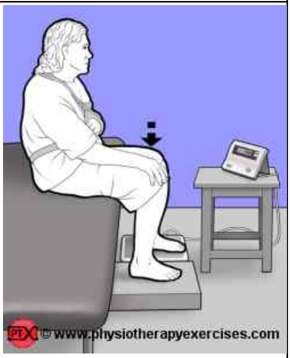

Propping in side-sitting	Boxing in sitting	Reaching to the side in wheelchair	Moving around the bed in short sitting
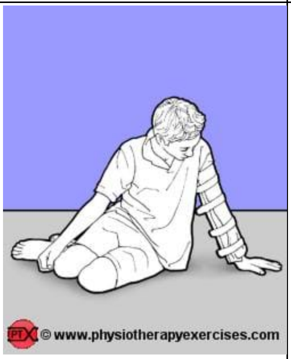	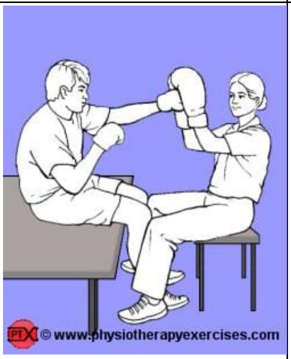	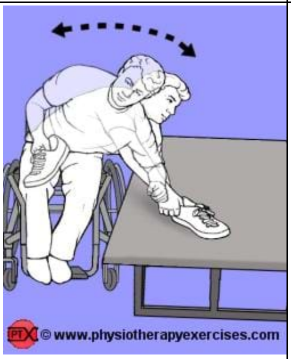	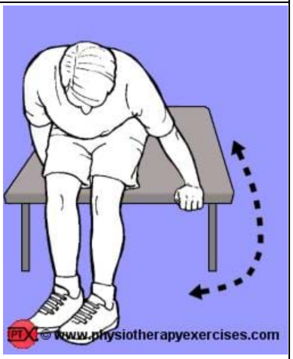

3.4 Motor training exercises to improve the ability to stand

Bilateral squat on tilt table	Single-leg squat on a tilt table	Bilateral squat in standing	Squatting against a wall
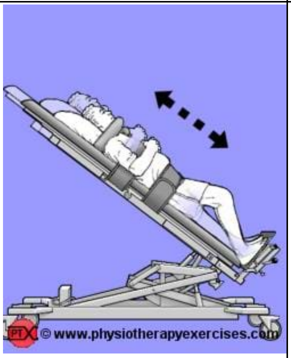	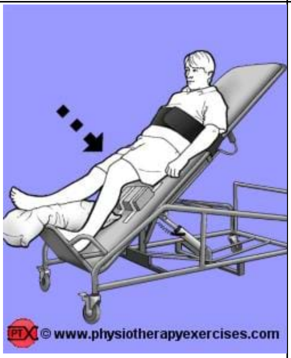	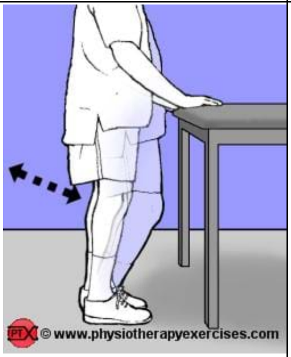	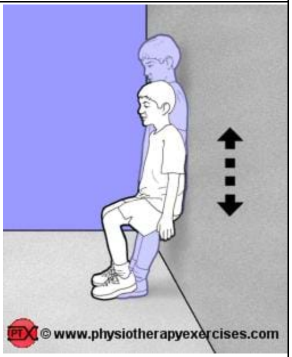

Single-leg squat against a wall	Single-leg squat	Bilateral squat to a target in standing	Knee extension in standing
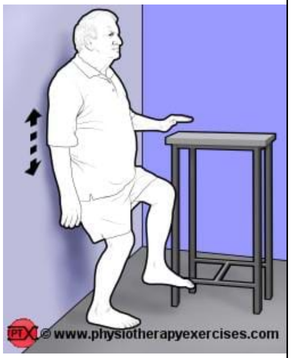	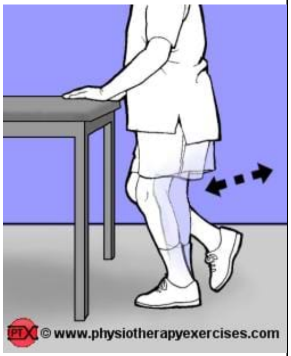	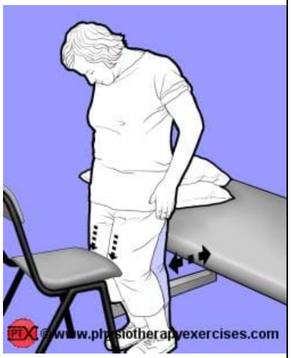	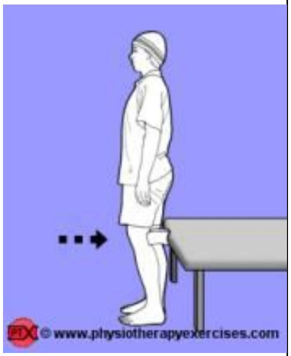

Standing and shifting weight using a harness	Transferring weight laterally in standing against two walls	Transferring weight laterally in standing beside a wall	Transferring weight laterally in standing with the back against a wall
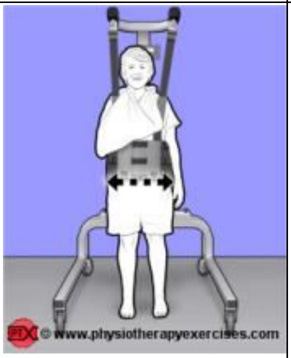	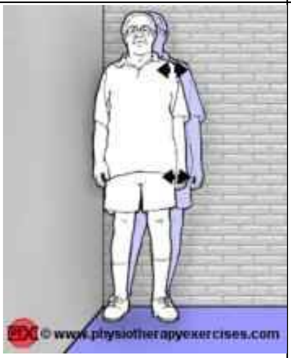	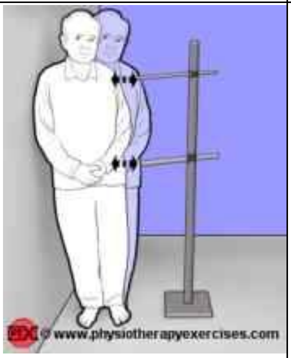	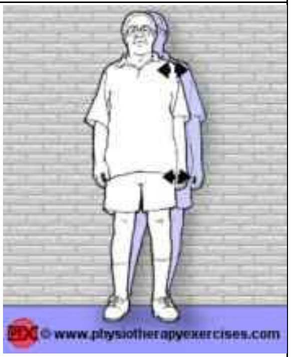

Standing and reaching to the side	Reaching from side to side in standing	Stand and shift weight forwards and backwards	Standing and looking up and down
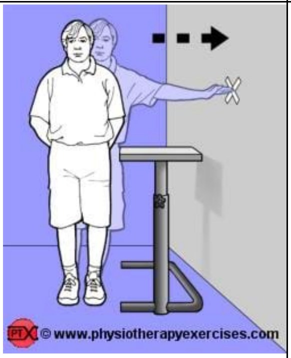	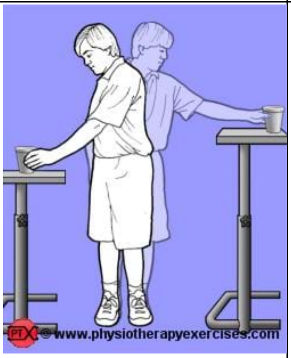	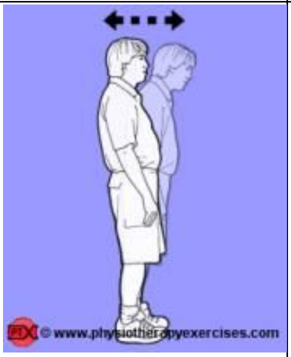	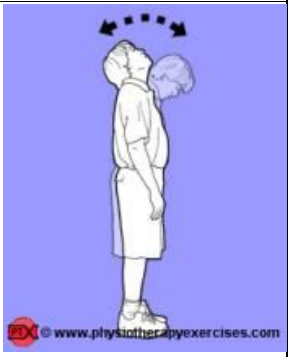

Stand and look behind	Stand with one leg forward and look behind	Stand with narrow base of support	Stand in semi-tandem stance
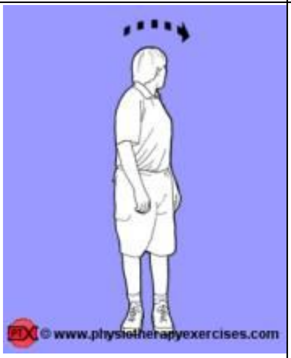	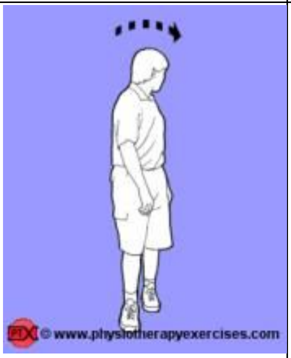	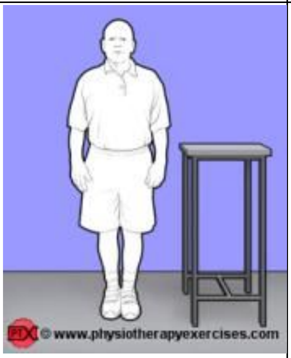	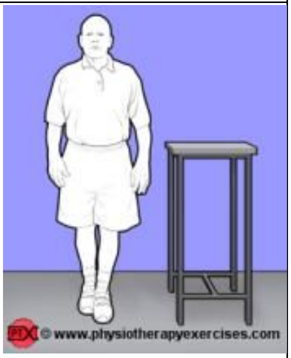

Stand in tandem stance	Standing and reaching	Stepping forwards to reach for an object	Standing and reaching from the floor to above the head
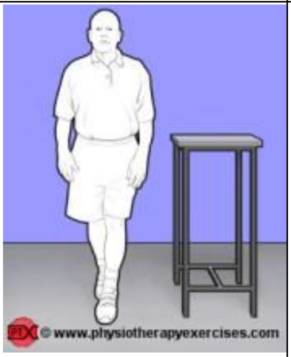	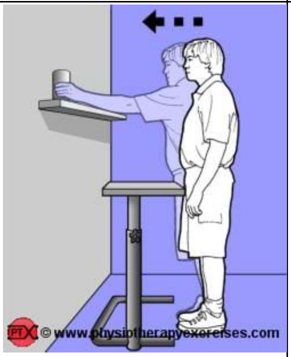	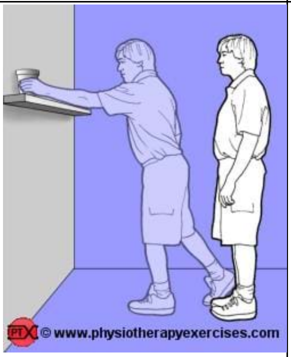	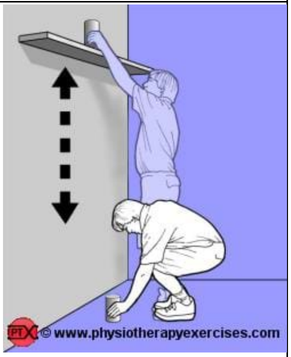

Stand, throw and catch with one hand	Stand, throw and catch with two hands	Standing and picking up an object off the floor with both hands	Standing and picking up an object off the floor
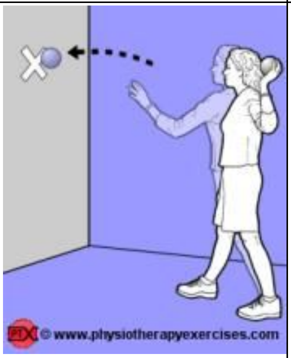	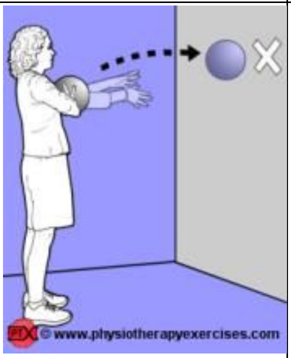	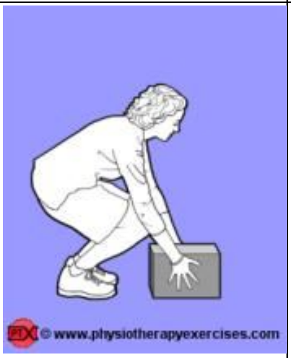	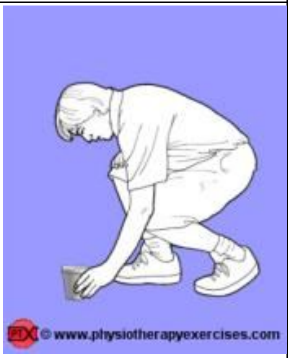

3.5 Motor training exercises to improve the ability to step and walk

Walking on a treadmill in a harness with assistance	Walking on a treadmill in a harness with pillowcase to assist swing	Walking on a treadmill in a harness with theraband to assist swing	Walking in a harness to targets
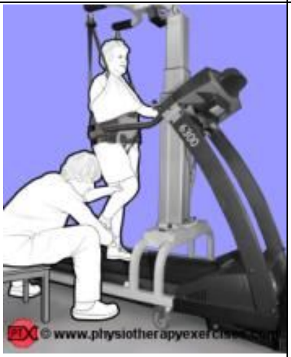	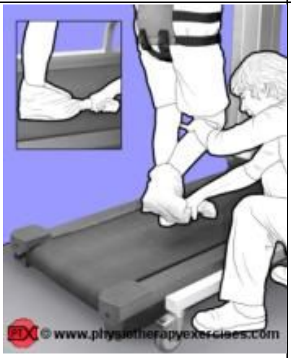	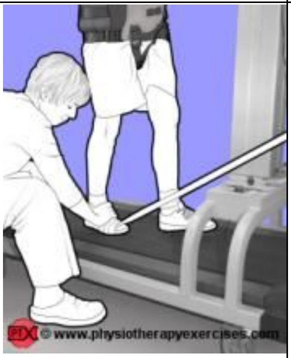	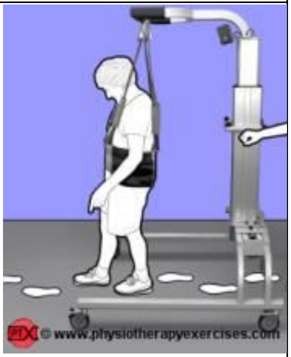

Walking in a harness	Walking on the spot on a treadmill in a harness to the beat of a metronome	Marching on the spot	Stepping forwards to a cue
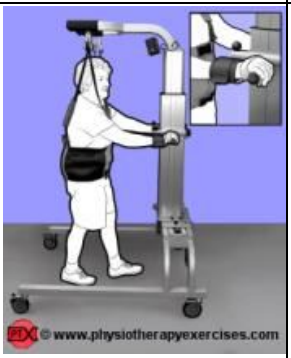	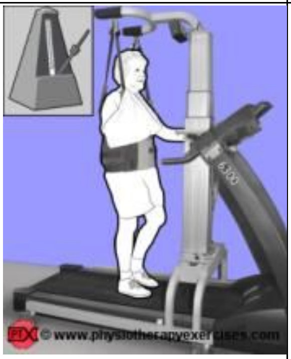	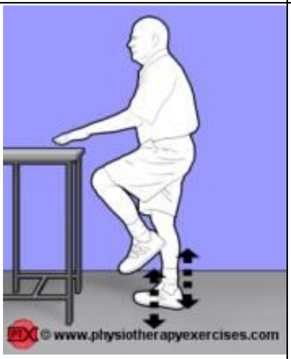	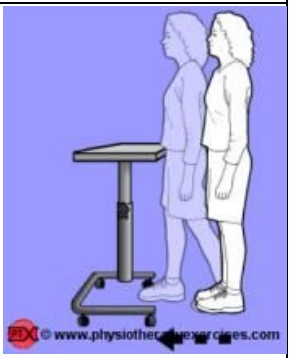

Stepping	Walking forwards over lines	Knee control in standing	Standing and bending and straightening the knees quickly
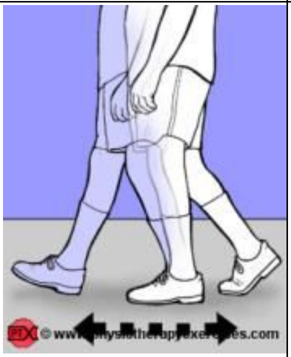	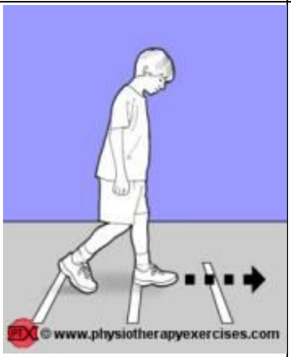	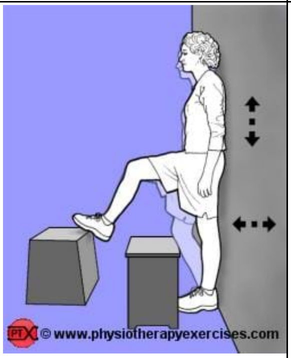	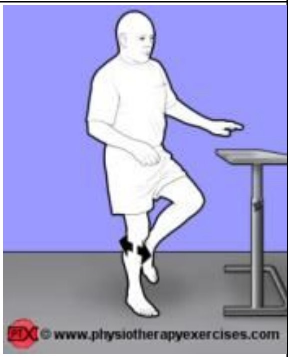

Standing on tip toes	Lunging forwards to a chair	Single-leg heel raises	Standing and elevating the pelvis
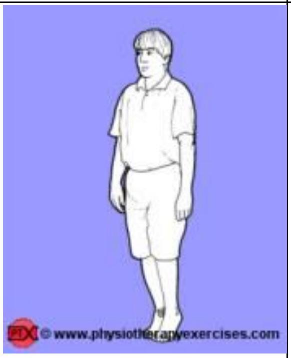	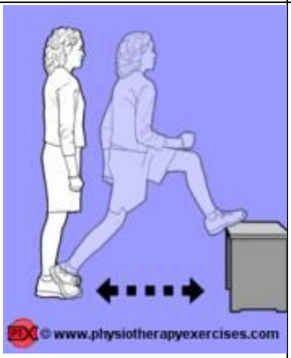	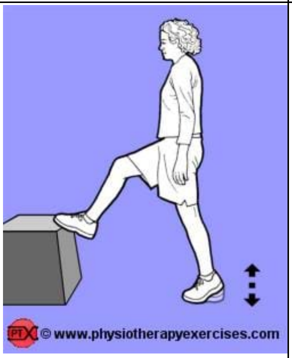	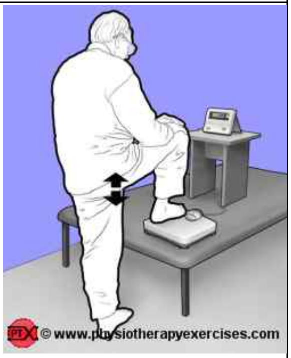

Lifting the leg sideways onto a block	Walking between lines	Stepping over a raised rope	Stand on one leg with support
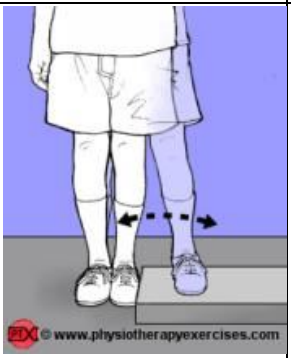	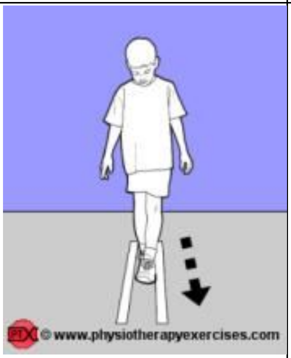	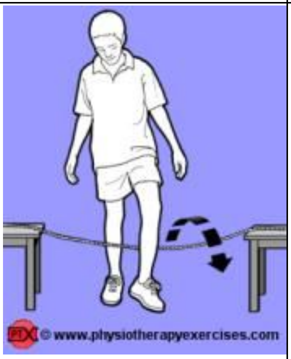	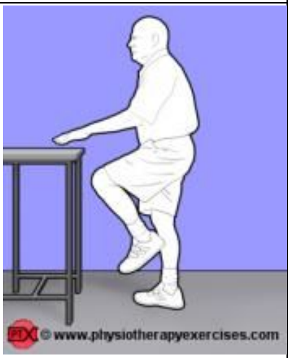

Stepping forwards over an obstacle with hand support nearby	Stepping sideways over an obstacle with hand support nearby	Standing on one leg with the other leg resting on a foam cup	Maintaining single-leg stance while moving the other foot to targets in a semi-circle
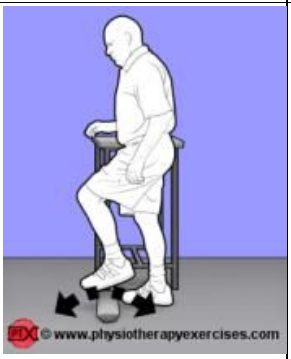	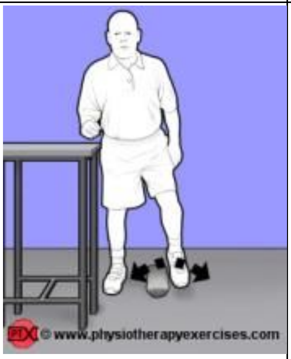	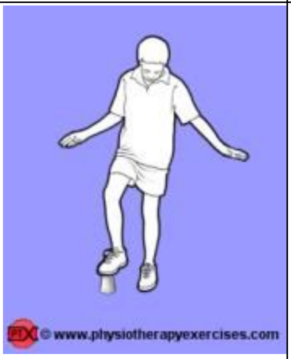	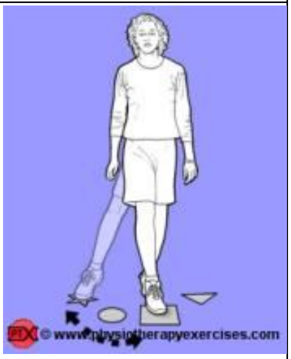

Maintaining single-leg stance while rolling the other foot on a ball	Maintaining single-leg stance while rolling a ball around an obstacle	Stand and pivot on one leg	Walking to the beat of a metronome
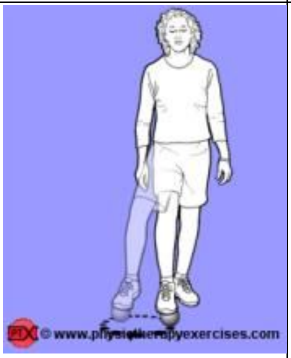	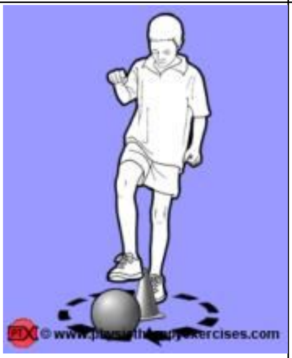	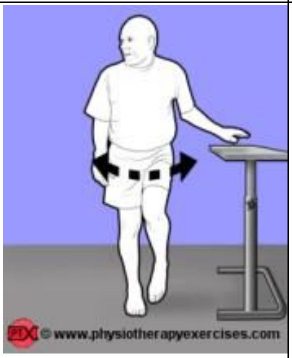	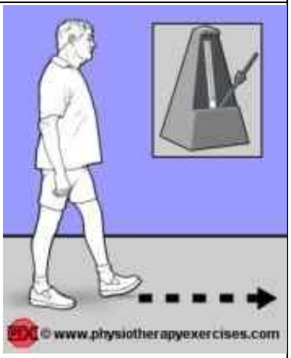

Walking a set distance within a set time	Lunge	Walking lunge	Stepping sideways and back with the back against a wall
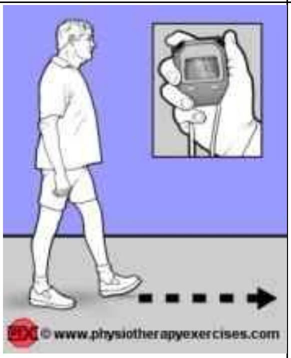	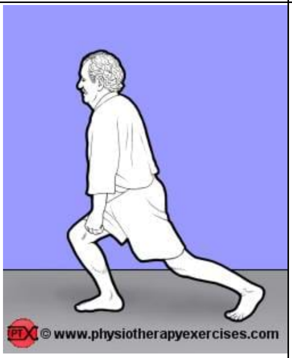	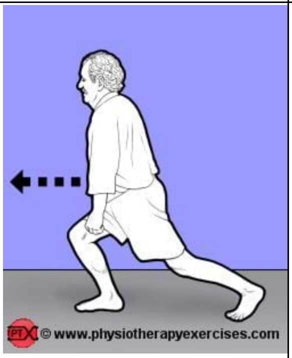	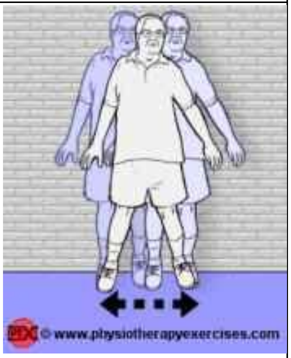

Stepping sideways	Walking sideways along a line	Turning around on the spot	Walking on a line while bouncing a ball
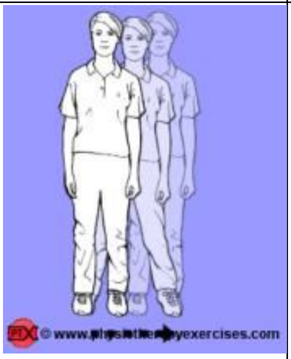	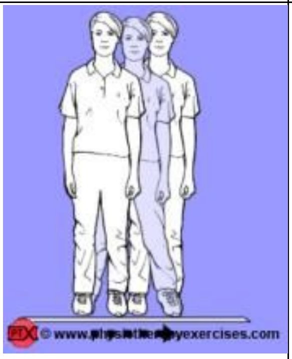	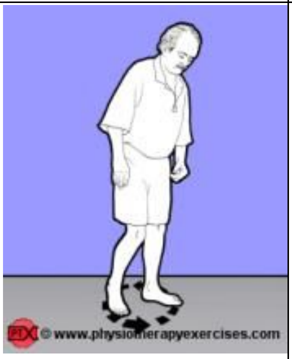	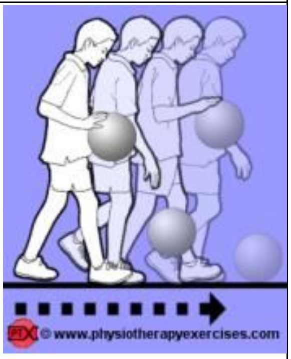

Walking over obstacles	Walking in a figure-of-eight	Walking on uneven ground	Walk heel to toe
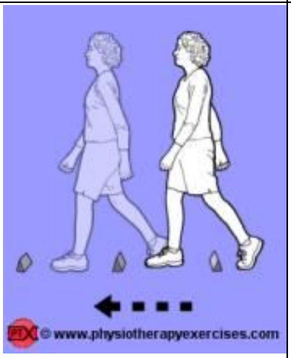	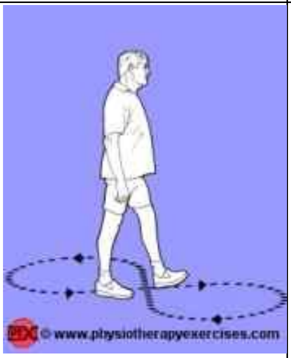	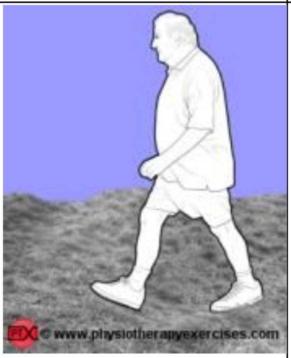	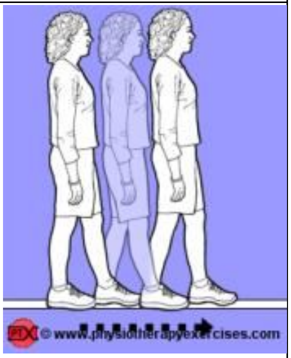

Walking while performing multiple tasks	Maintaining single-leg stance while stepping backwards with the other leg	Stepping backwards to a line with the affected leg	Walking backwards
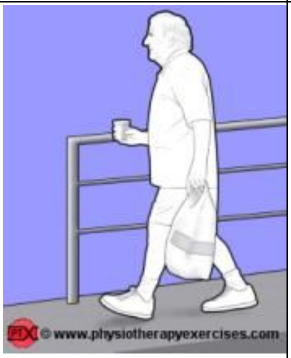	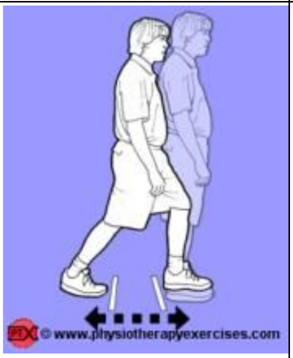	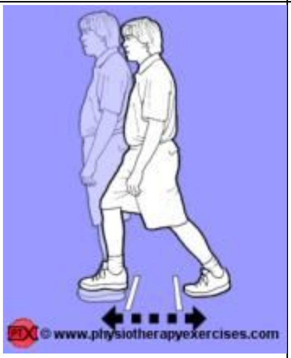	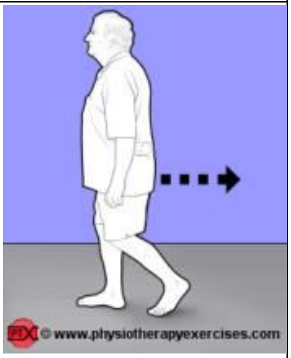

3.6 Motor training exercises to improve the ability to move from sit to stand

Moving the shoulders forwards in sitting using a moving table	Moving the shoulders forwards in sitting to a target	Flexing the hips in sitting and sliding arms forward on a table to a target	Reaching forward in sitting
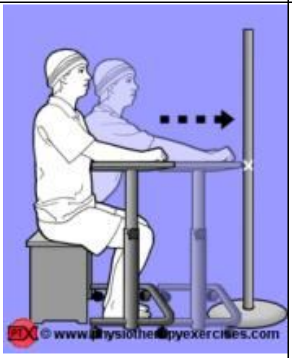	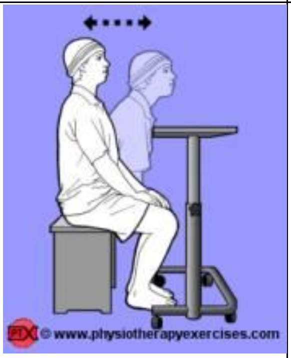	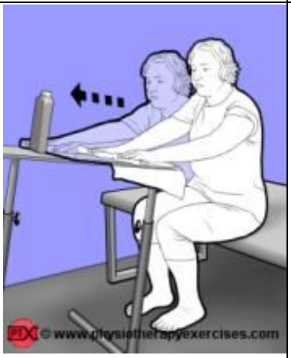	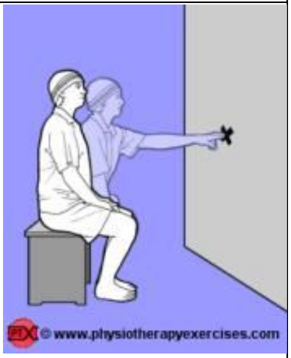

Pushing down through the leg in sitting	Pushing down through the leg when leaning forwards	Standing up and sitting down with a harness	Standing up and sitting down with arm support
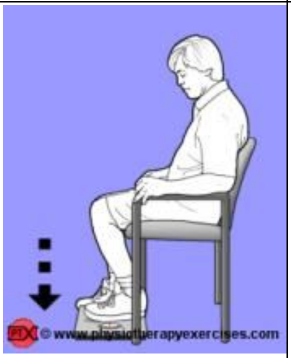	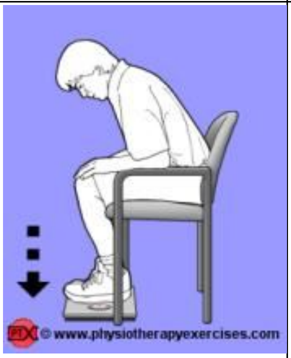	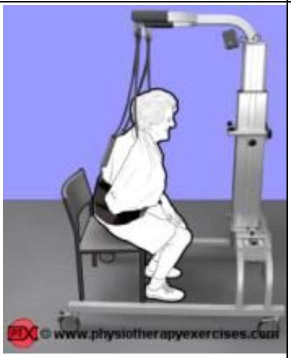	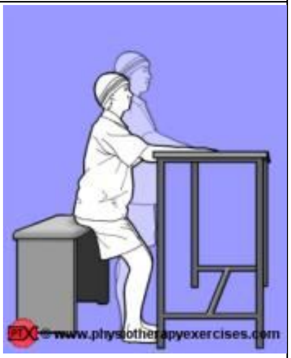

Standing up and sitting down from a high surface	Standing up and sitting down to a knee cue	Standing up and sitting down with foam between the knees	Weight-bearing through affected leg when standing up using scales for feedback
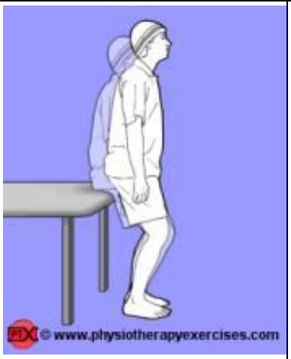	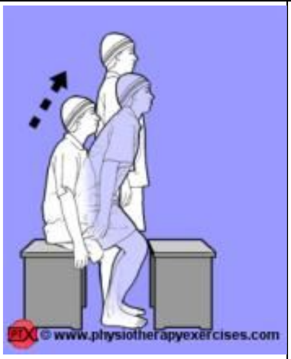	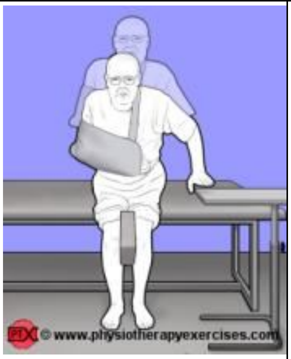	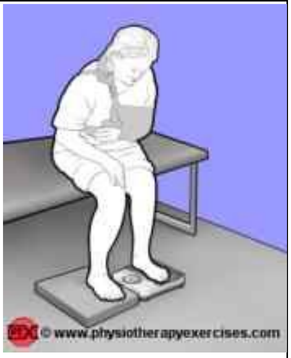

Standing up and sitting down using a limb-load monitor	Standing up and sitting down using a lateral knee cue	Standing up and sitting down with hand support nearby	Standing up and sitting down
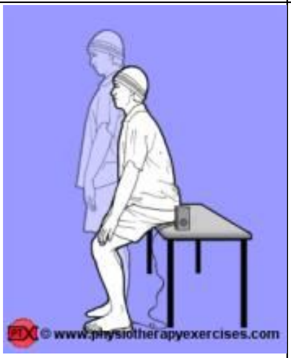	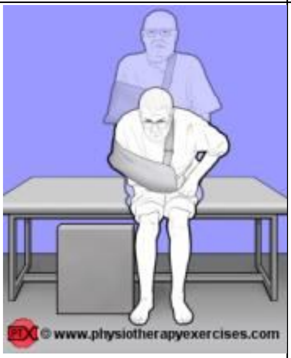	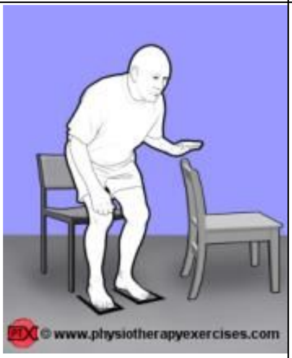	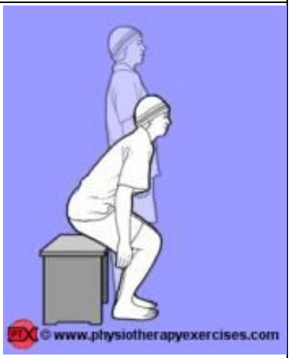

Reaching to the side when standing up	Standing up and sitting down from a dining table	Standing up and sitting down while holding onto an object	Standing up and sitting down from a low stool
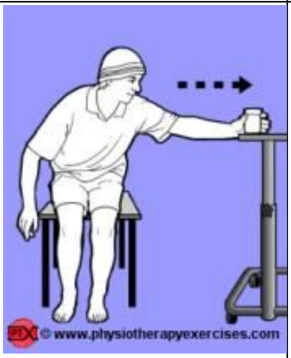	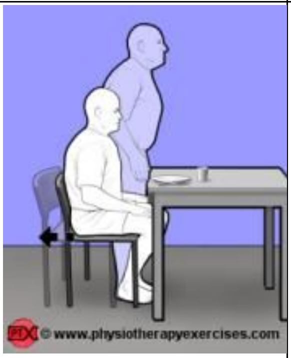	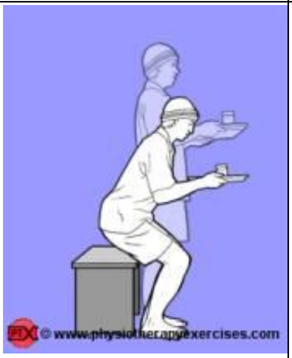	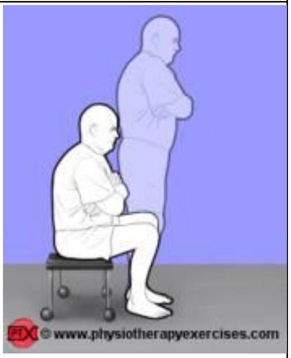

Standing up and sitting down while wearing a weight belt	Standing up with one leg on a block	Standing up with one leg forward	Standing up and walking
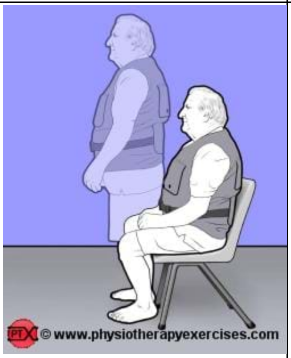	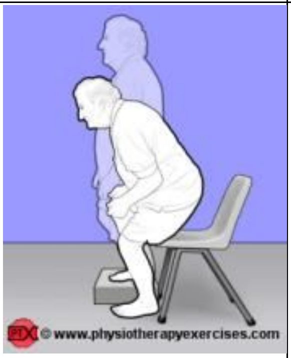	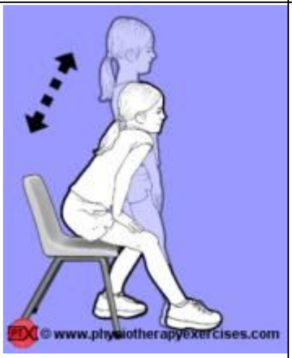	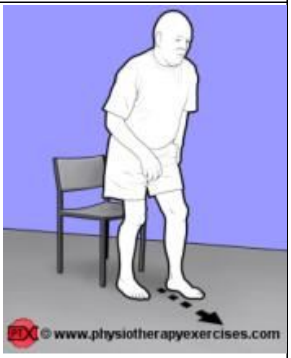

3.7 Motor training exercises to improve the ability to ascend/descend steps

Maintaining single-leg support while stepping up and down stairs	Maintaining single-leg stance while stepping	Maintaining single-leg stance while stepping from a block	Lowering and raising from a block
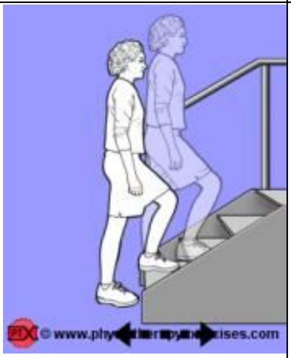	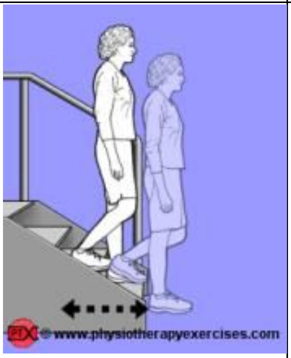	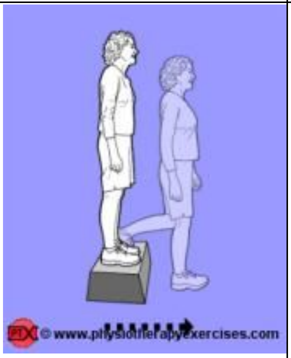	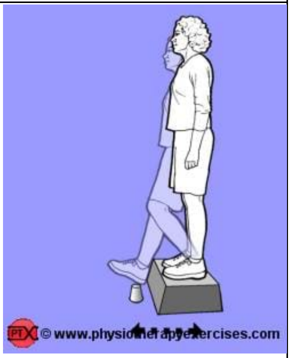

Walking sideways up and down stairs	Raising and lowering from a block with hand support nearby	Maintaining single-leg stance while touching the other foot on a block	Maintaining single-leg stance while touching the other foot on a block
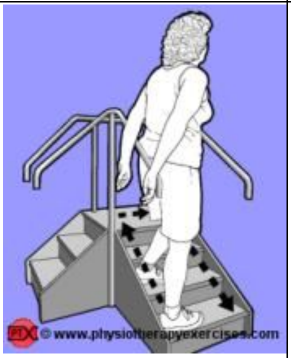	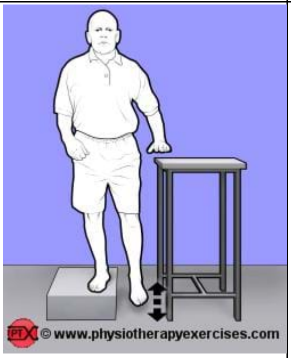	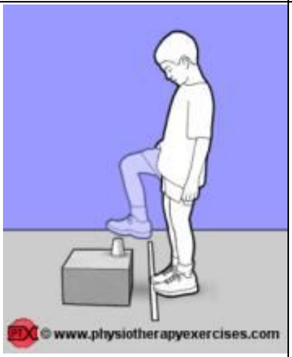	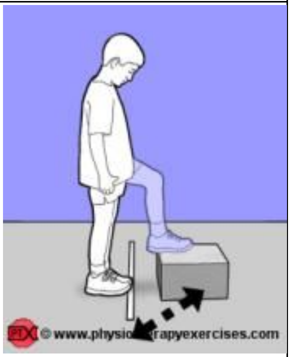

Stepping up onto a block	Stepping down from a block to a knee cue	Walking up stairs	Walking up a kerb
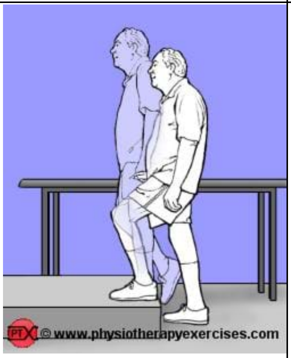	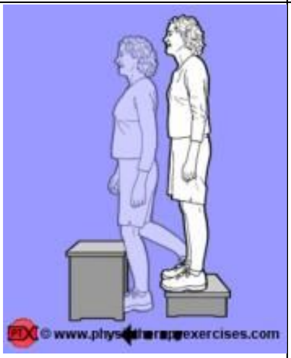	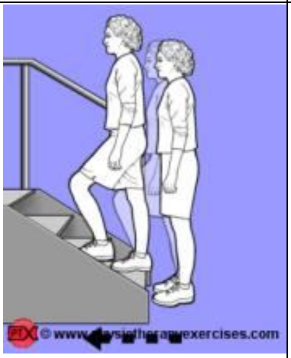	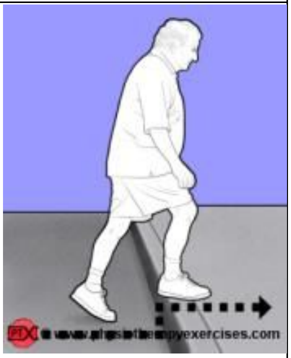

3.8 Motor training exercises to improve advanced gait related activities

(these types of exercises would rarely be appropriate because they require excellent strength and very advanced mobility skills. The occasional person may improve to this stage)

Kicking a ball	Leaping from a standing start and landing on one leg	Jumping forwards over lines	Hopping on the spot
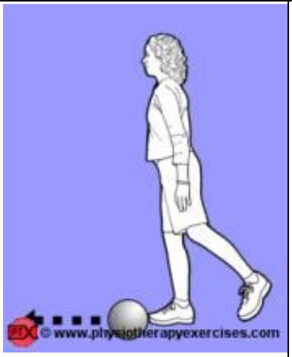	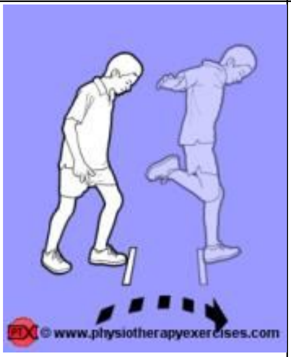	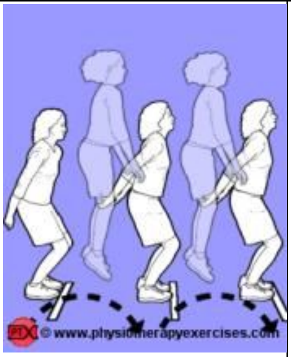	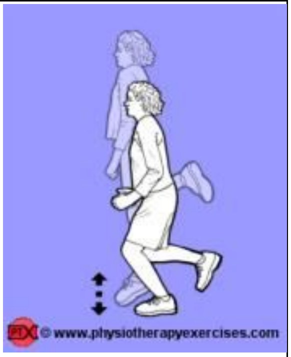

Hopping in different directions	Leaping from a standing start and landing on both legs	Jumping from side to side	Jumping in different directions
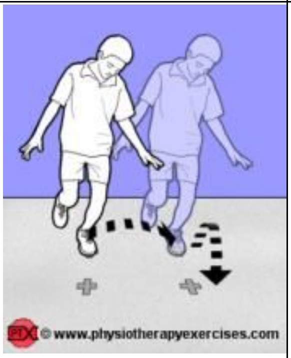	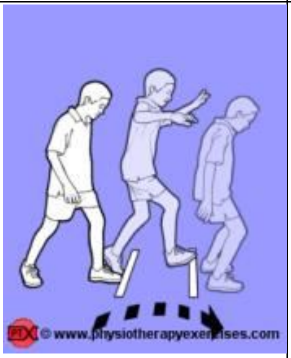	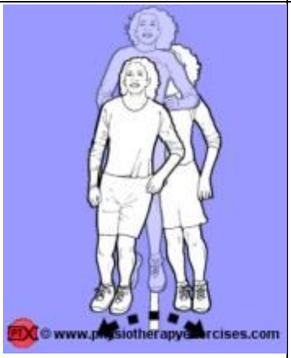	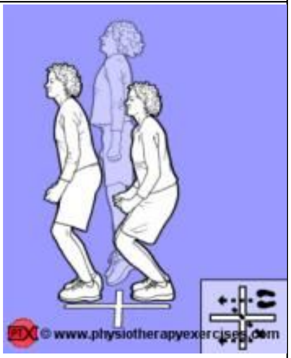

Leaping sideways	Cross over side step	Swinging the foot quickly up to a target	Skipping
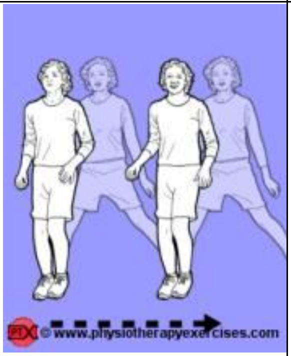	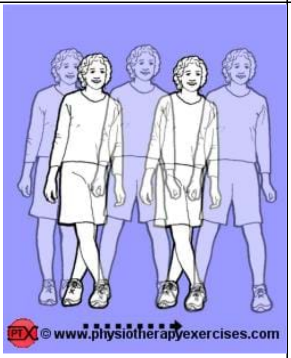	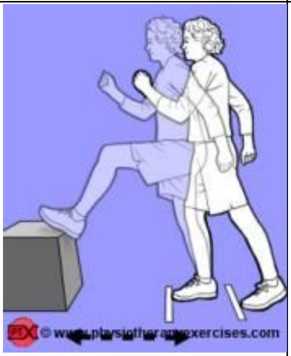	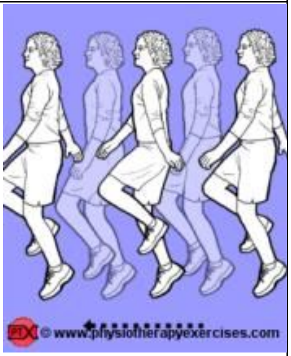

Hopping forwards over lines	Swivelling on the balls of the feet	Running on the spot	Running forwards over lines
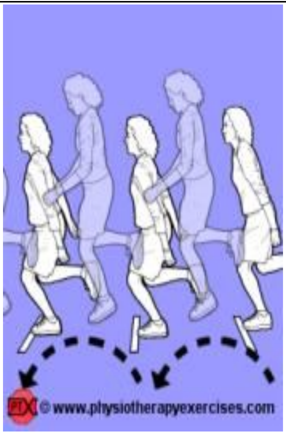	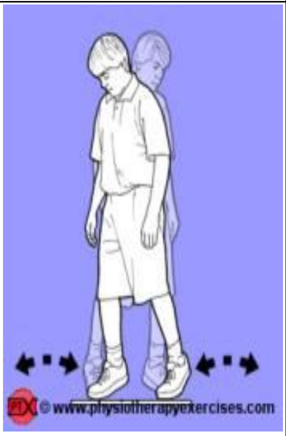	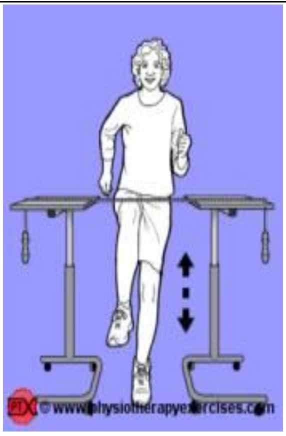	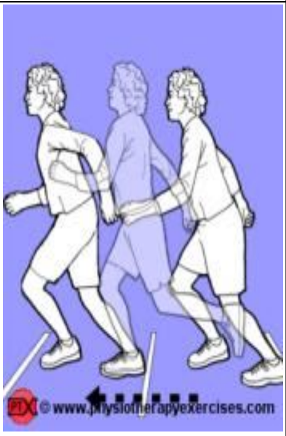

Running between lines	Jogging on a mini-trampoline	Running around obstacles	Bouncing a ball with one hand
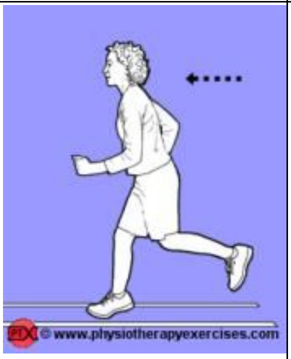	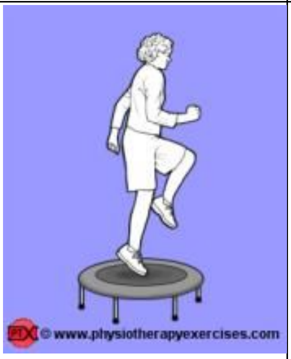	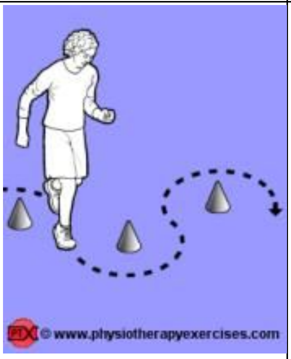	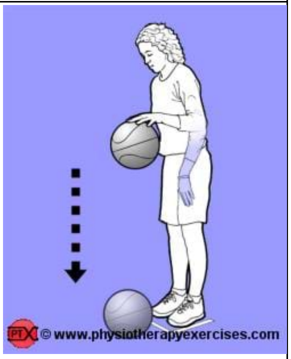

4 STRENGTH TRAINING EXERCISES

These are intended as a guide only to illustrate some of the different types of possible strength training exercises that could be used to improve function and mobility. The exercises can be varied according to a participant's strength and ability. Some of the exercises could be categorised/administered as motor training exercises provided they are administered using the principles of motor training. The exercises would only be appropriate if they targeted muscles below the level of the injury and could conceivably increase Total Motor Scores.

Note on all images used in this manual

- All images are copied with permission from www.physiotherapyexercises.com; freely available exercise-prescribing software designed for, and by, physiotherapists for people with injuries and disabilities.
- The images reflect physical presentations of people with different types of Neurological Impairments, and are not always representative of people with SCI. They are only intended to convey the principles of an exercise, and to be used as cues and prompts. They may need to be modified to the needs of each person.
- The titles of the exercises are the same as used in www.physiotherapyexercises.com but do not always fully convey the intention of the exercise.
- Further details about each exercise can be found on www.physiotherapyexercises.com including suggestions on how to make the exercise easier or harder. Use the title of each exercise as "key words" to search. Then, right click on the matching image to see the full details.

4.1 Strength training exercises for upper limb muscles (grade 1 and 2 strength)

**Shoulder muscles (grade 1 and 2)**			
Shoulder flexor/extensor strengthening in sidelying using a slideboard	Shoulder flexor/extensor strengthening in sidelying using slings	Shoulder flexor/extensor strengthening using slings and targets in sidelying	Shoulder flexor strengthening -Sliding the hand forwards on a table
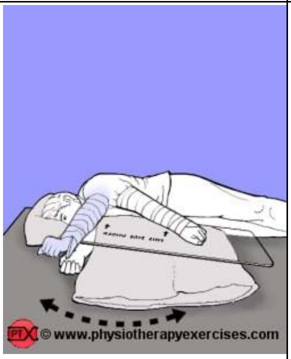	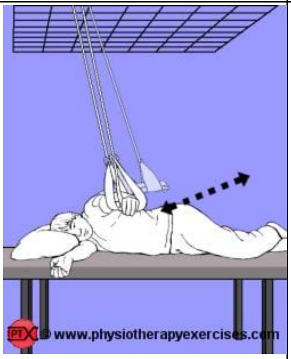	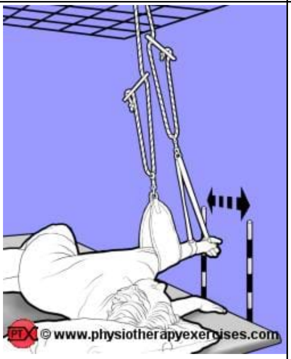	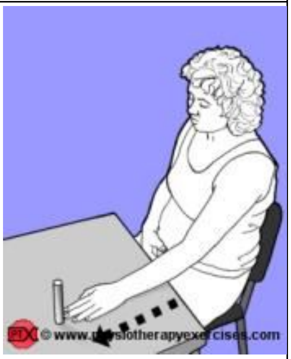

Shoulder abductor and adductor strengthening in supine using slings	Rotating the shoulder to a target	Rotating the shoulder outwards while holding a cup	Shoulder protractor/retractor strengthening in sitting using slings
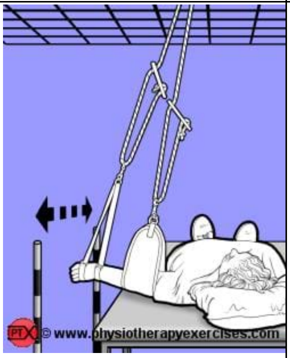	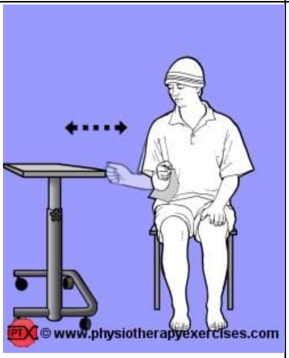	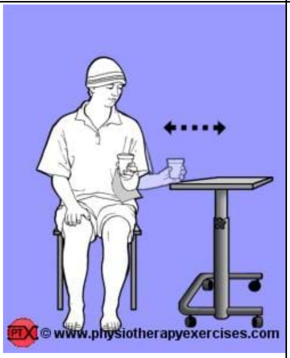	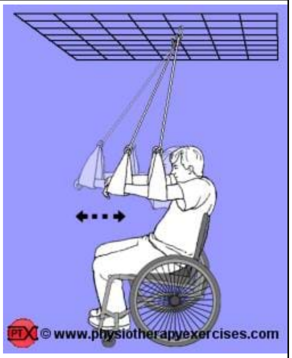

Sliding the arm forwards on a table	Shoulder retractor strengthening in sitting	Shoulder horizontal abductor/adductor strengthening using slings	Shoulder depression with arm in abduction
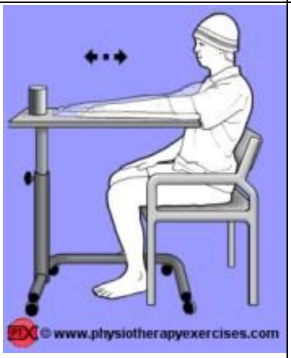	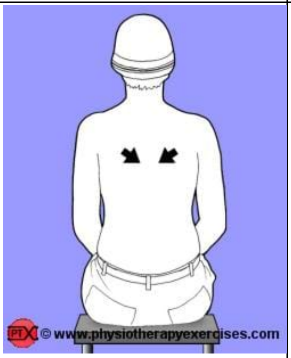	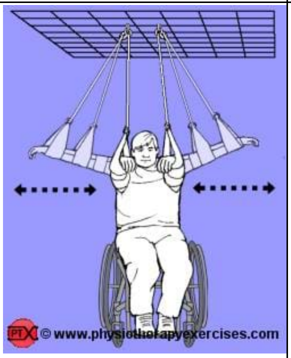	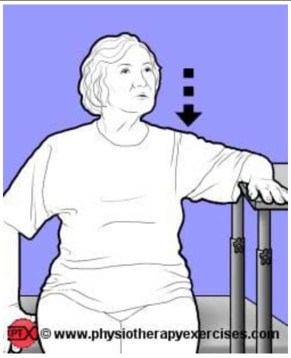

**Elbow muscles (grade 1 and 2)**			
Elbow flexor/extensor strengthening in sidelying using slings	Elbow flexor/extensor strengthening in sitting using slings	Rotating the forearm to targets	Forearm supination and pronation to a wall target
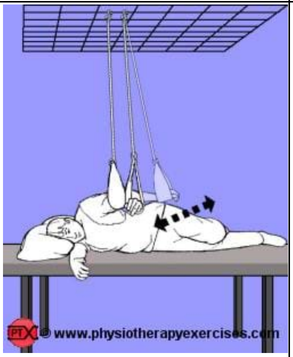	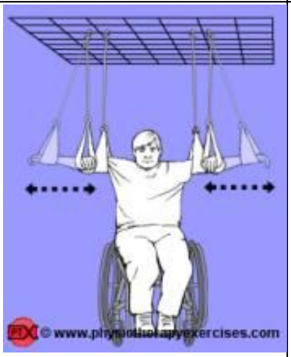	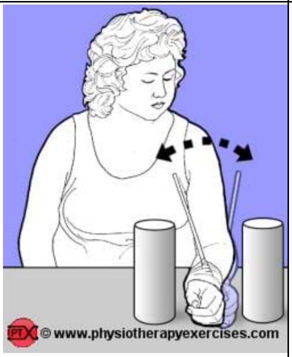	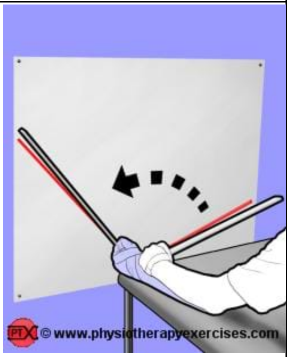

**Wrist and hand muscles (grade 1 and 2)**			
Bending and straightening the wrist to targets	Cupping the hand on a table	Touching the thumb to each fingertip	Straightening and bending the fingers using targets
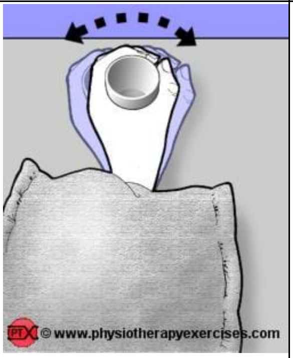	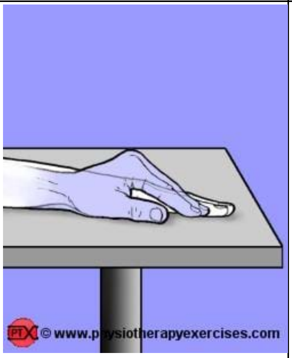	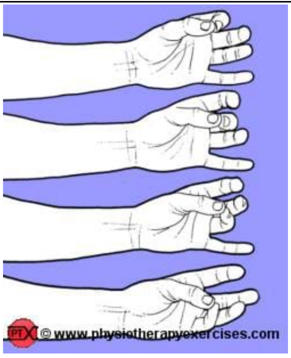	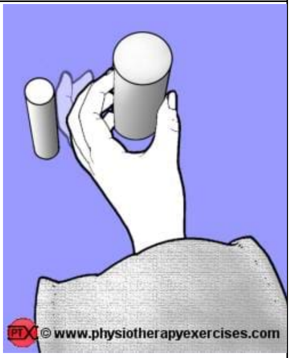

Thumb abduction: sliding the thumb on a stick with hand on top of cup	Sliding the thumb on a stick with hand around cup	Abducting the thumb to a target	Sliding the thumb on a ruler
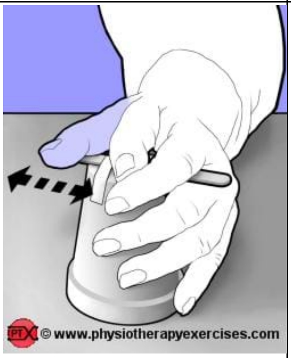	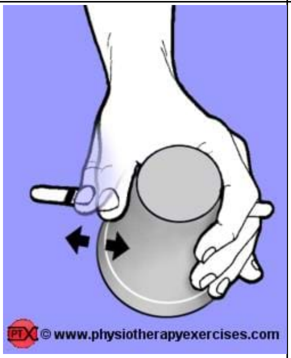	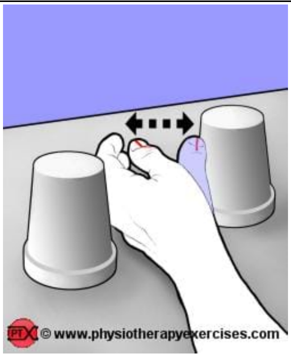	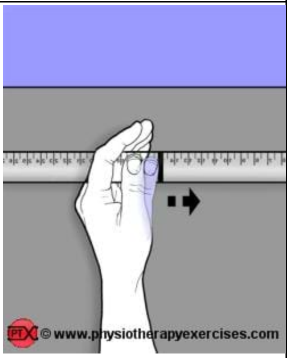

4.2 Strength training exercises for upper limb muscles (grade 3 and 4 strength)

Shoulder muscles (grade 3 and 4)

(the exercises with elbow extension splints are for those with very weak or paralysed triceps)

Moving the extended arm between two targets	Shoulder flexor strengthening in supine using free weights	Shoulder flexor strengthening in supine using theraband	Shoulder extensor/flexor strengthening in prone using free weights
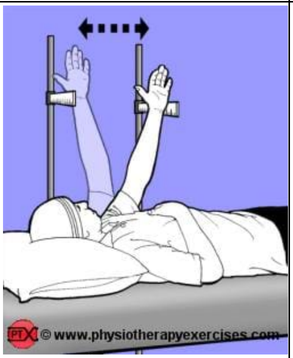	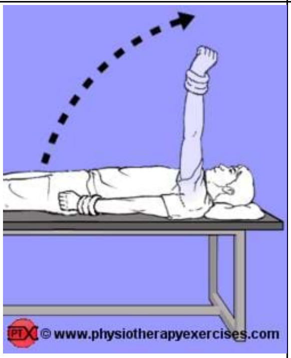	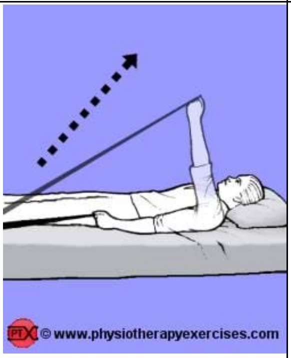	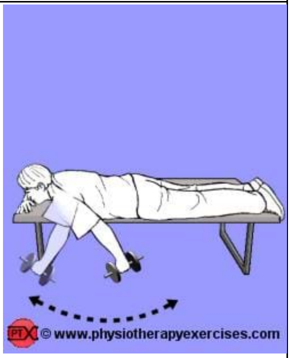

Shoulder flexor strengthening in sitting using theraband	Shoulder flexor strengthening in sitting using Uppertone	Shoulder flexor strengthening in sitting using pulleys	Lifting up an object
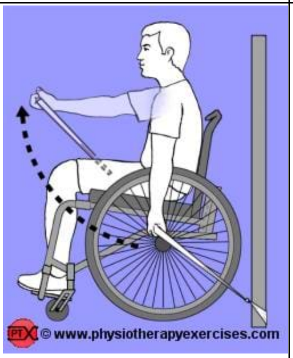	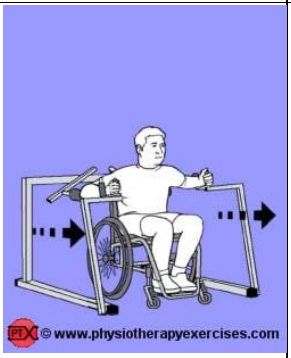	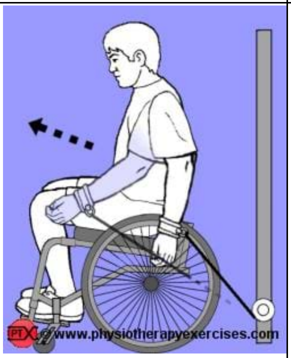	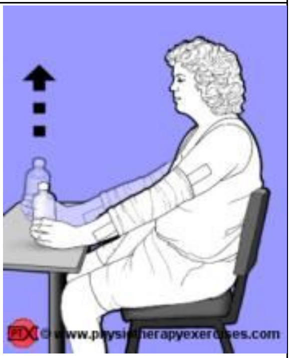

Lifting the arm in sitting	Shoulder flexor strengthening in standing using theraband	Shoulder horizontal abductor strengthening using free weights	Shoulder horizontal abductor strengthening in sitting using theraband
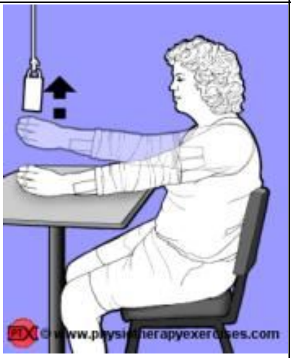	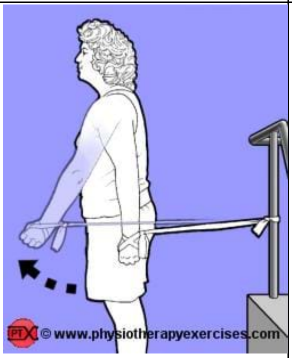	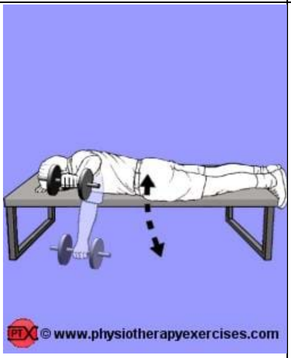	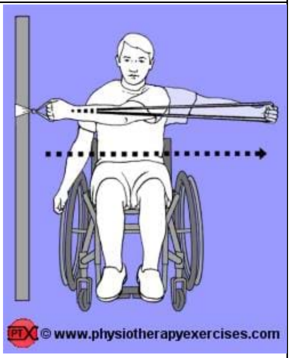

Shoulder horizontal abductor strengthening in sitting using pulleys	Shoulder horizontal adductor strengthening in supine using free weights	Shoulder horizontal adductor strengthening in sitting using theraband	Shoulder horizontal adductor strengthening in sitting using pulleys
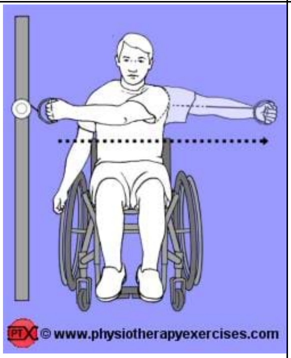	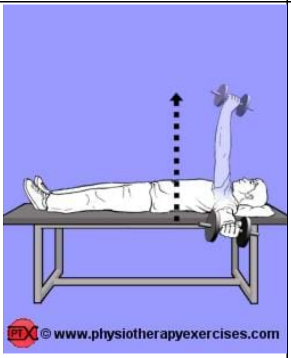	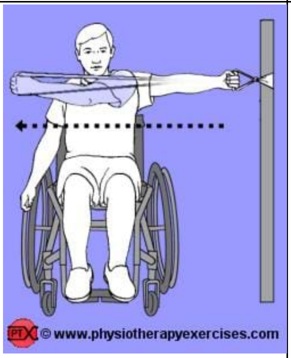	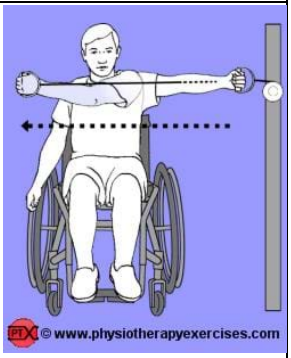

Bench press	Shoulder extensor strengthening in supine using theraband	Shoulder extensor strengthening in sitting using theraband	Shoulder extensor strengthening in sitting using pulleys
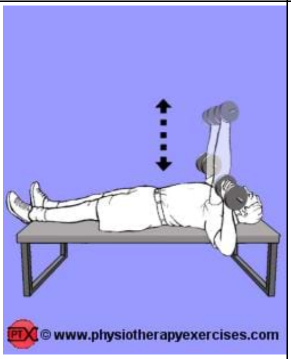	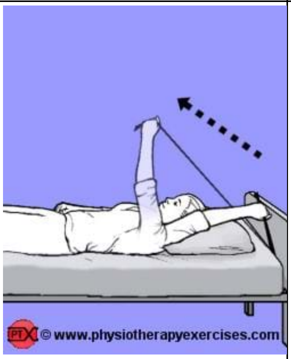	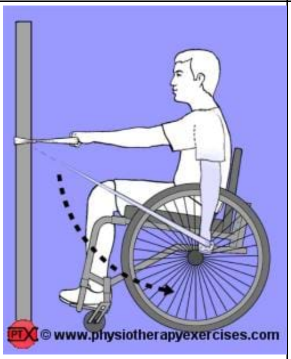	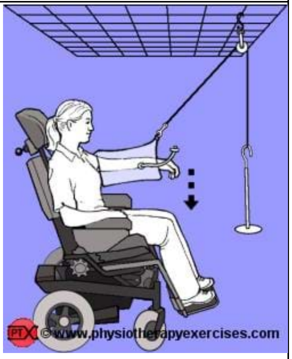

Shoulder retractor strengthening in sitting using pulleys	Shoulder extensor strengthening in sitting using pulleys	Shoulder extensor strengthening in sitting using free weights	Shoulder extensor strengthening in sitting using theraband
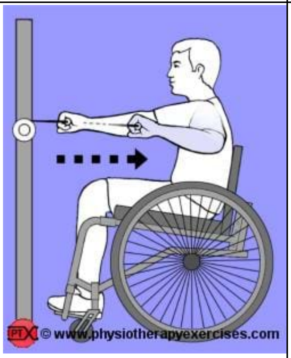	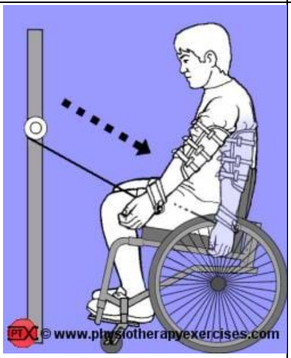	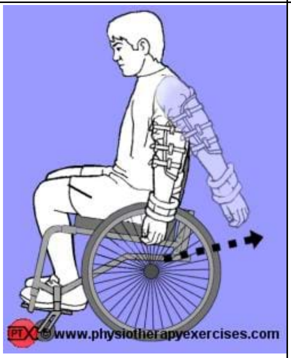	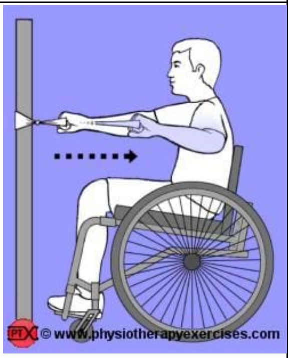

Shoulder extensor strengthening in sitting using theraband	Shoulder extensor strengthening in sitting using pulleys	Shoulder extensor strengthening in sitting using Uppertone	Shoulder extensor strengthening in standing using theraband
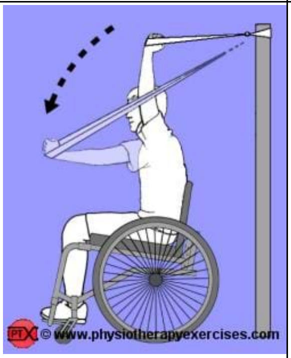	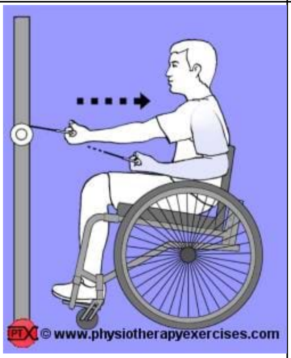	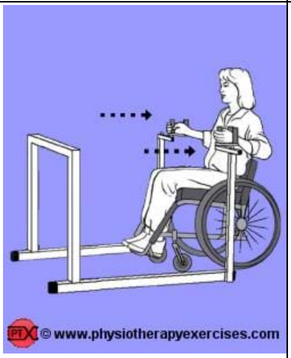	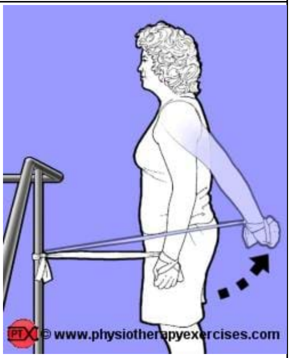

Shoulder abductor strengthening in sitting using theraband	Shoulder abductor strengthening in sitting using free weights	Shoulder abductor strengthening in sitting using pulleys	Shoulder abductor strengthening using Uppertone
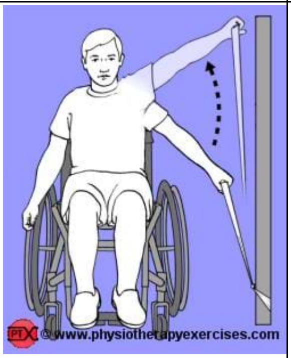	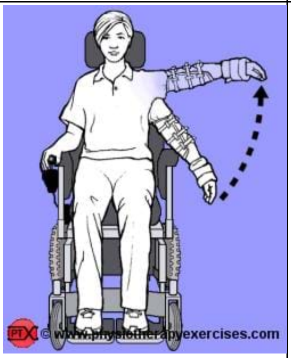	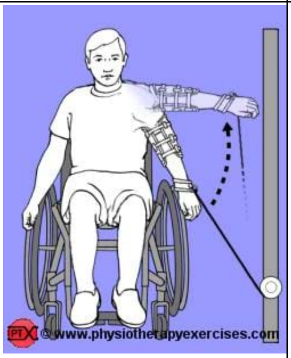	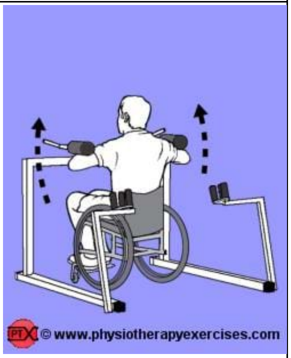

Shoulder abductor strengthening in standing using theraband	Shoulder adductor strengthening in sitting using theraband	Shoulder adductor strengthening in sitting using pulleys	Shoulder adductor strengthening in standing using theraband
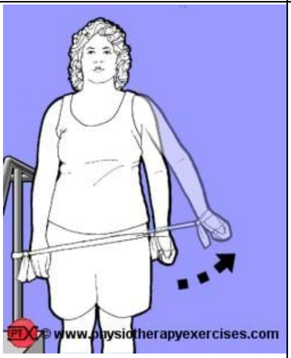	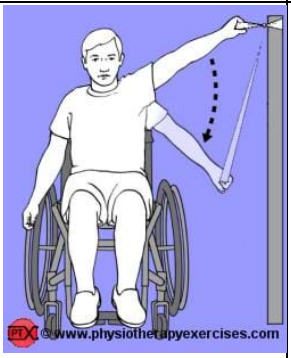	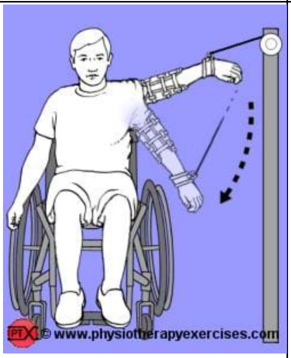	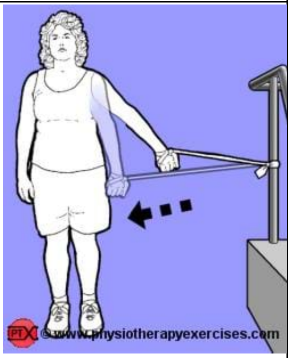

Shoulder external/internal rotator strengthening in supine using springs	Shoulder external rotator strengthening in sidelying using free weights	Shoulder external rotator strengthening in supine using free weights	Shoulder external rotator strengthening in prone using free weights
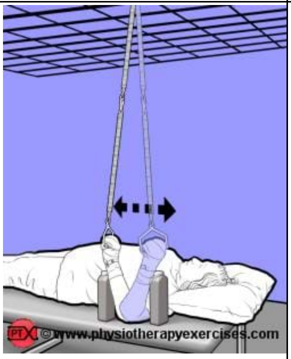	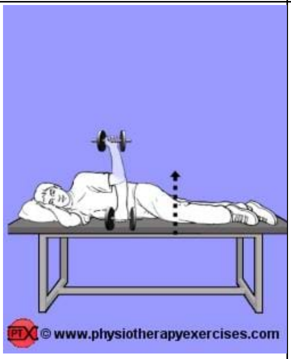	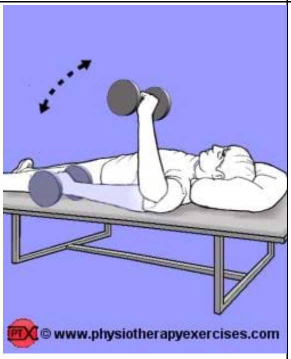	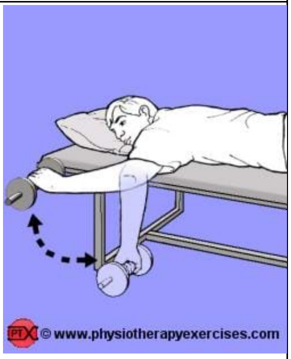

Shoulder external rotator strengthening in supine using theraband	Shoulder external rotator strengthening in sitting using theraband	Shoulder external rotator strengthening in sitting using pulleys	Shoulder external rotator strengthening in sitting using free weights with arm supported
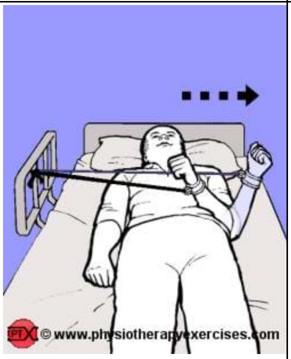	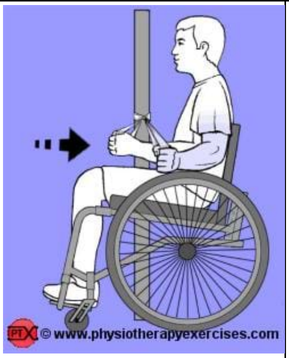	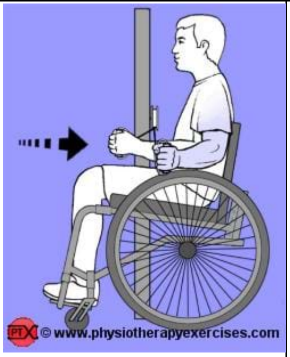	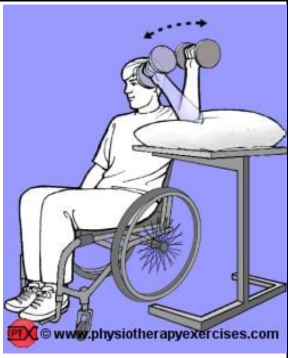

Shoulder external rotator strengthening in sitting using Uppertone	Shoulder external rotator strengthening in standing using theraband	External rotation at 90 degrees with theraband	Shoulder internal rotator strengthening in supine using theraband
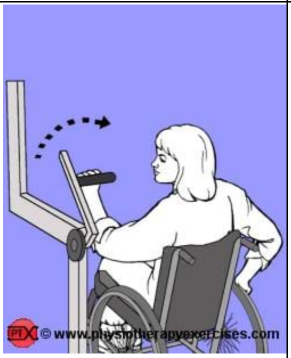	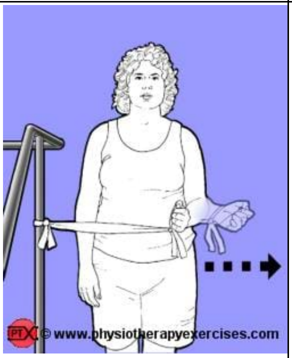	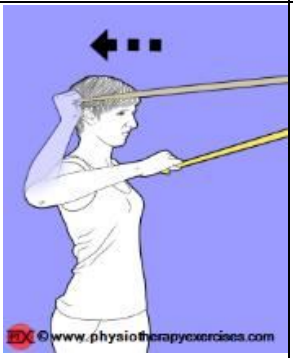	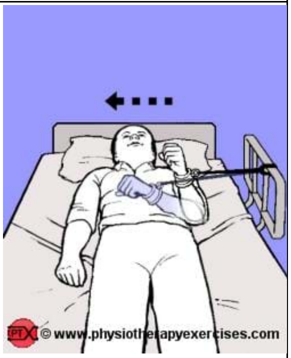

Shoulder internal rotator strengthening in sitting using pulleys	Shoulder internal rotator strengthening in sitting using theraband	Shoulder internal rotator strengthening in standing using theraband	Shoulder internal rotator strengthening in 90 degrees shoulder abduction using theraband
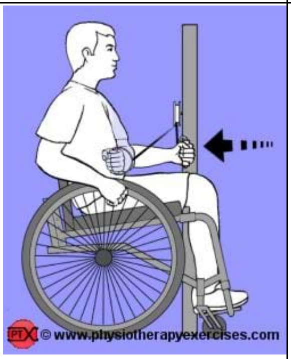	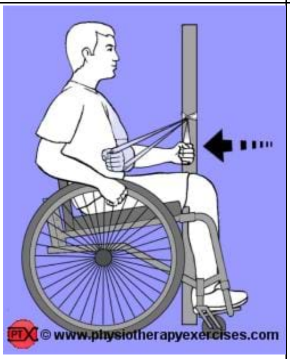	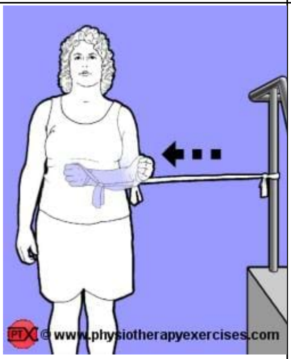	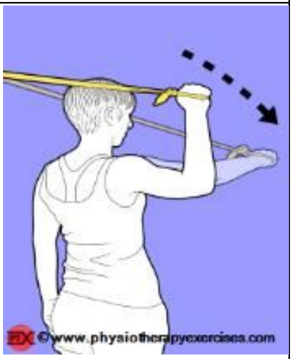

Shoulder depressor strengthening in sitting using Uppertone	Extending the arm to a target in lying	Lifting in short sitting with a second plinth in front	Push-ups between two plinths
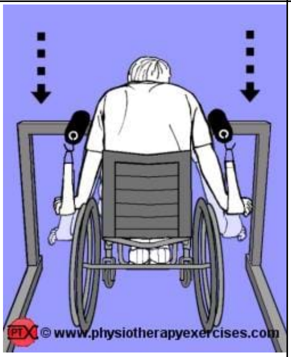	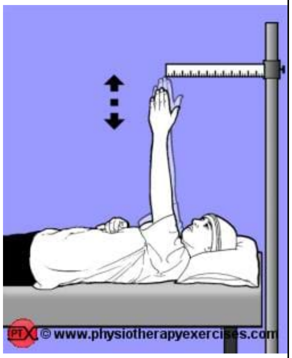	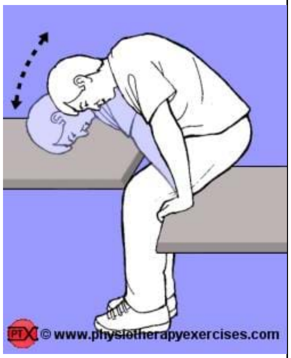	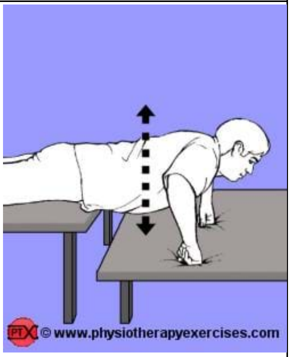

**Elbow muscles (grade 3 and 4)**			
Elbow extensor strengthening in prone using free weights	Elbow extensor strengthening in supine using theraband	Elbow extensor strengthening in supine using free weights	Elbow extensor strengthening in lying without weights
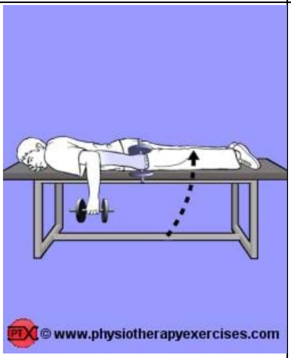	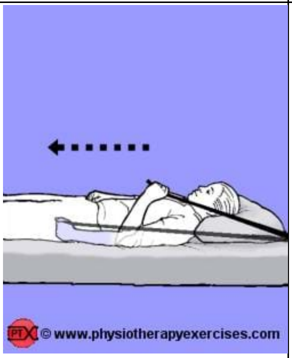	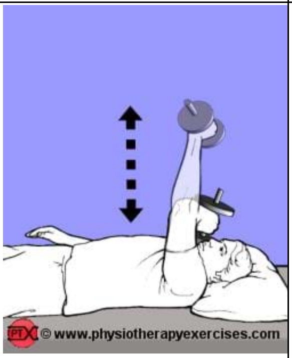	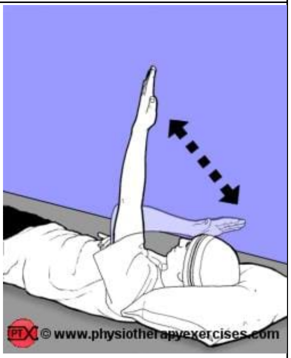

Elbow extensor strengthening in supine using theraband	Elbow extensor strengthening in sitting using pulleys	Elbow extensor strengthening in sitting using free weights	Elbow extensor strengthening in sitting using theraband
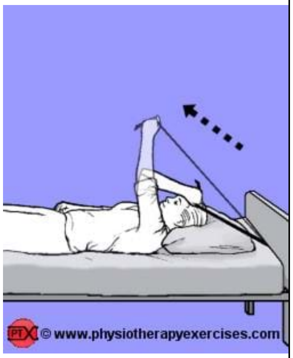	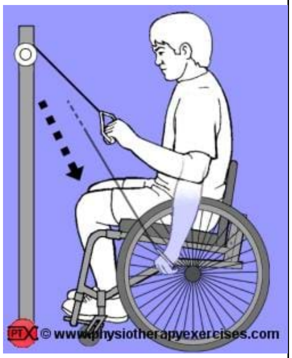	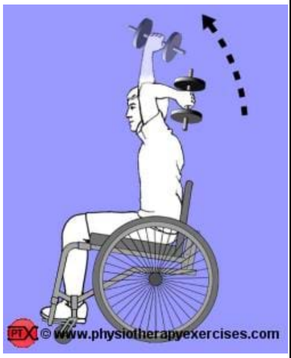	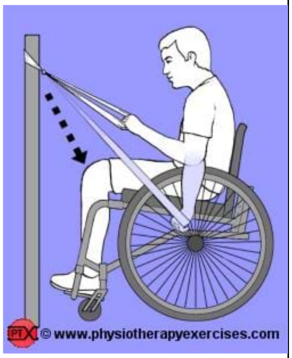

Elbow extensor strengthening in sitting using Uppertone	Elbow flexor strengthening in supine using free weights	Elbow flexor strengthening in supine using theraband	Elbow flexor strengthening in sitting using free weights
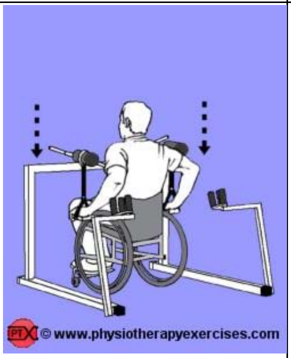	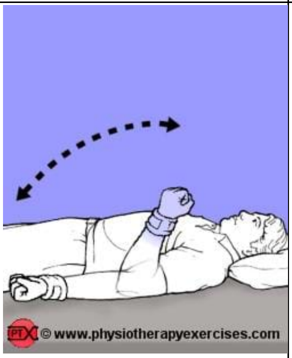	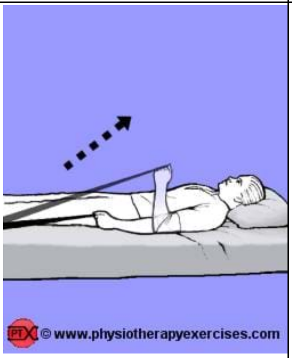	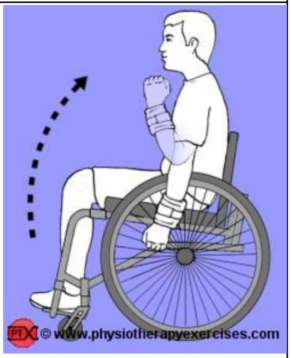

Elbow flexor strengthening in sitting using pulleys	Elbow flexor strengthening in sitting using theraband	Forearm supination and pronation to a wall target	Seated push-up
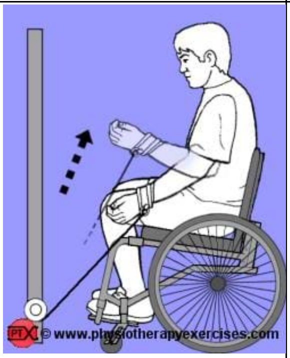	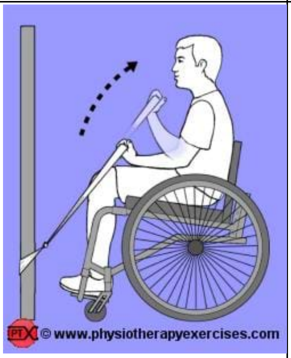	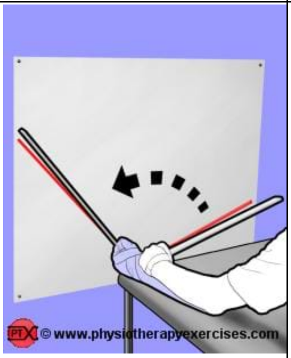	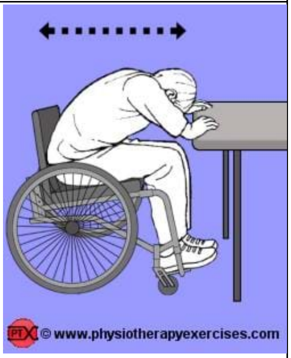

**Wrist and hand muscles (grade 3 and 4)**			
Wrist extensor strengthening in supine using free weights	Bending the wrist to a target	Wrist extensor strengthening in sitting using free weights	Wrist extensor strengthening in sitting using theraband

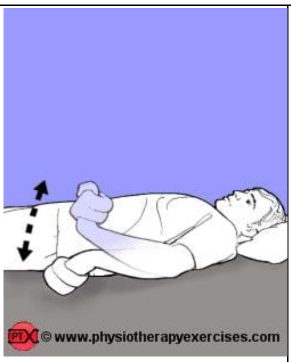	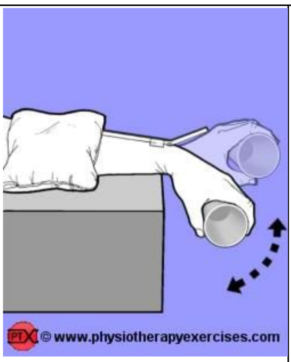	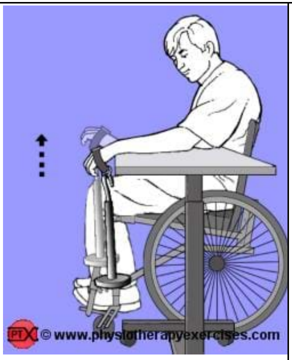	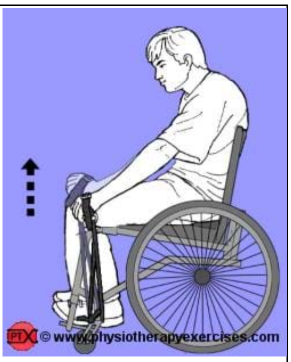
Bending the wrist while holding a cup	Maintaining wrist extension and radial deviation	Wrist flexor strengthening in supine using free weights	Wrist flexor strengthening in sitting using free weights
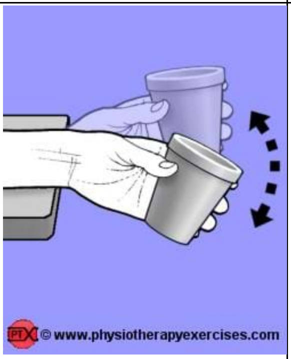	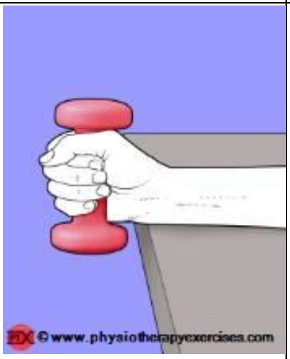	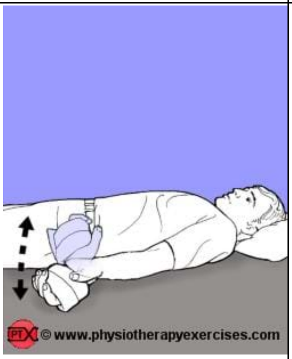	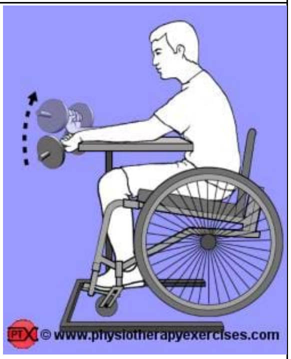

Finger and thumb extensor strengthening using an elastic band	Finger and thumb flexor strengthening using grip device	Finger flexor and intrinsic muscle strengthening	Finger extensor strengthening in sitting without weights
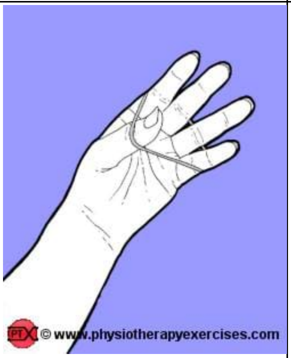	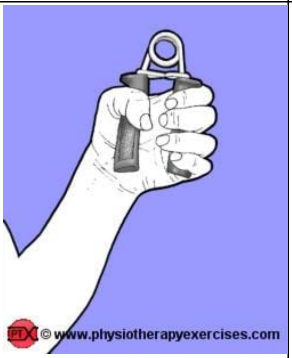	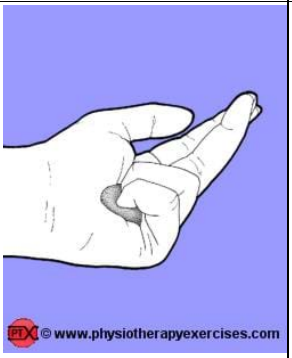	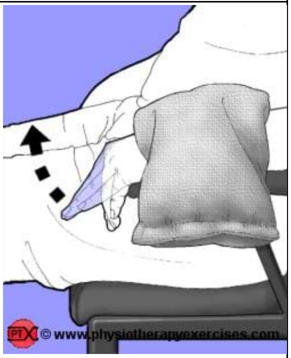

Pincer grip strengthening	Cupping the hand while picking up a plate	Controlling grip force by lifting a sealed bottle and straw	Finger and thumb flexor strengthening using foam
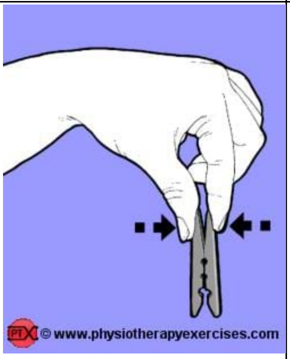	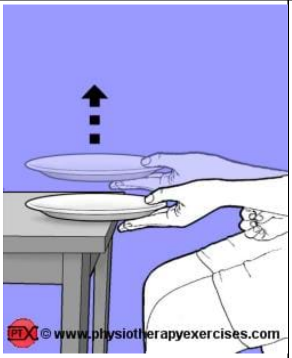	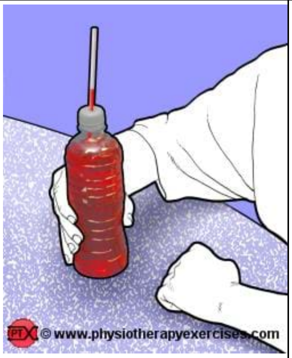	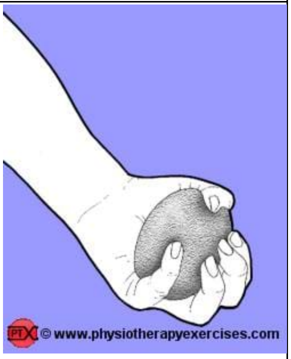

4.3 Strength training exercises for lower limb muscles (grade 1 and 2 strength)

**Hip muscles (grade 1 and 2)**			
Hip extensor/flexor strengthening in sidelying without weights	Hip extensor/flexor strengthening in sidelying using a slideboard	Hip extensor and flexor strengthening in sidelying using slings	Hip extensor and flexor strengthening in sidelying using a skateboard
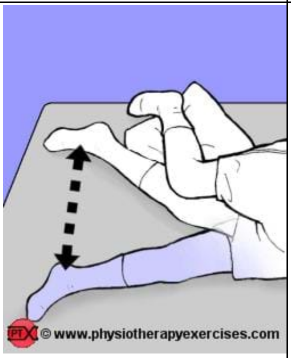	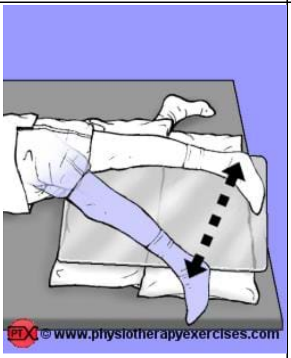	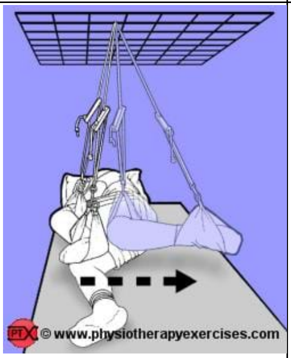	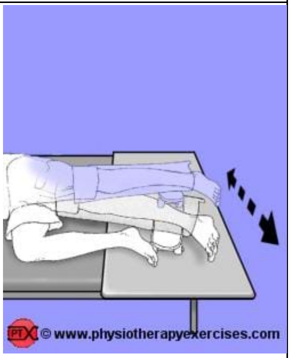

Hip extensor and flexor strengthening in sidelying using slings and targets	Hip abductor strengthening using slings	Hip abductor/adductor strengthening using a skateboard in supine	Hip abductor/adductor strengthening in supine

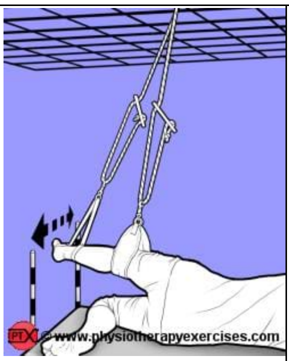	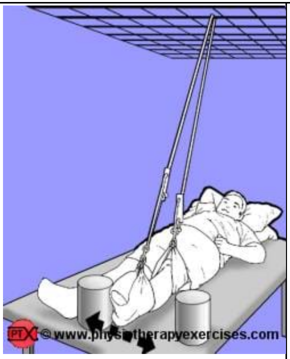	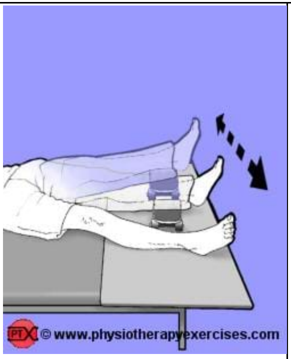	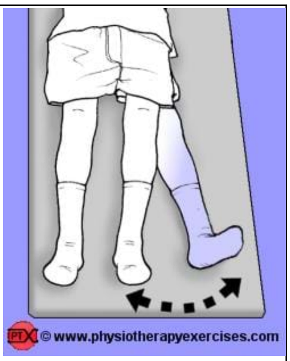
Hip external rotator strengthening in sitting without weights	Hip internal rotator strengthening in lying using slings	Hip internal rotator strengthening in supine using a stool	Hip internal rotator strengthening in sitting without weights
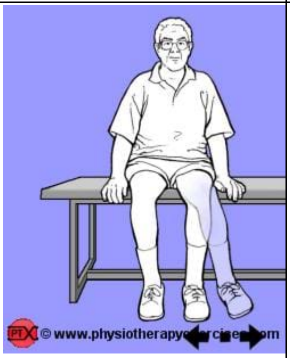	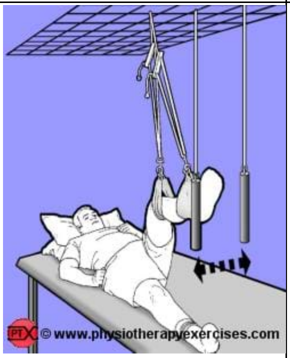	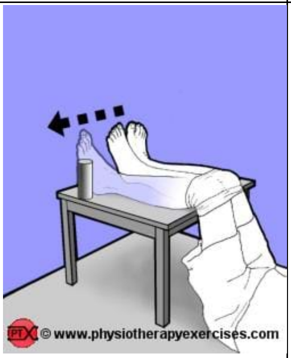	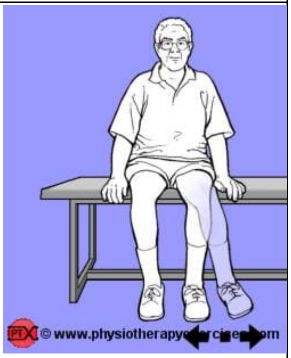

**Knee muscles (grade 1 and 2)**			
Knee extensor and flexor strengthening in sidelying using slings and targets	Knee flexor/extensor strengthening in sidelying using slideboard	Knee extensor strengthening in long sitting using biofeedback	Knee flexor strengthening in supine using skateboard
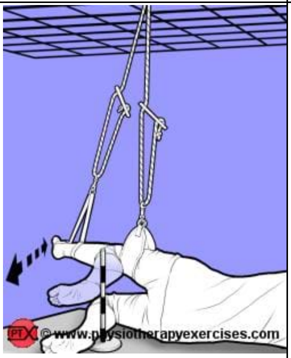	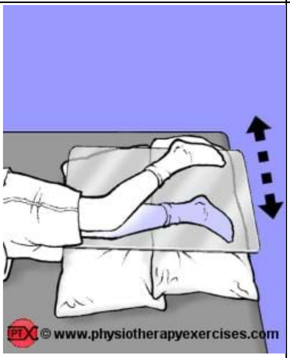	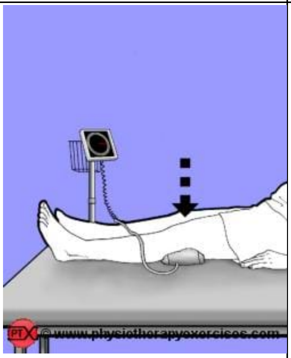	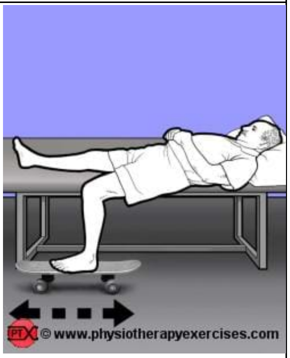

Bending the knee in sitting	Knee flexion in sitting using slidesheet	Bending the knee in sitting using a roller-skate	Knee flexor and extensor strengthening in sitting using a skateboard
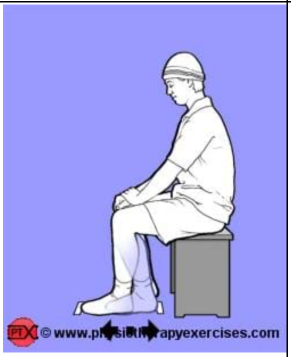	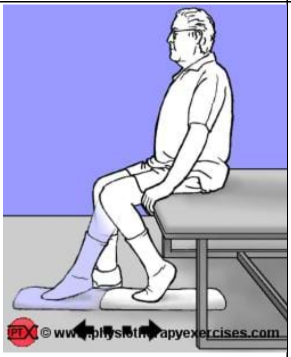	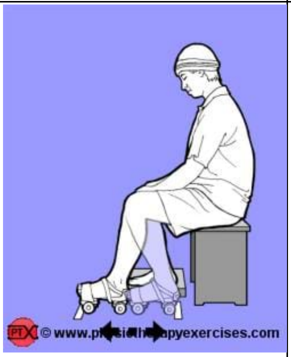	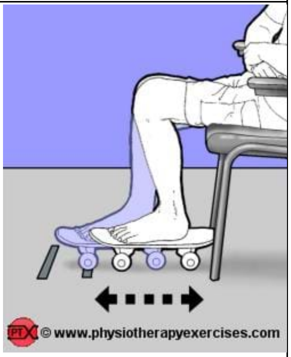

**Ankle muscles (grade 1 and 2)**			
Ankle dorsiflexor/plantarflexor strengthening in sidelying without weights	Ankle dorsiflexor/ plantarflexor strengthening in supine	Ankle evertor strengthening in sitting	Ankle invertor/evertor strengthening in sitting without weights
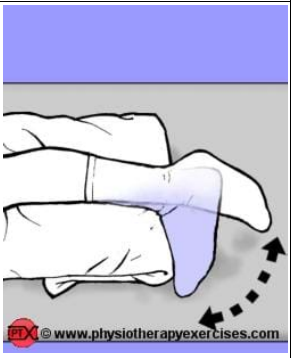	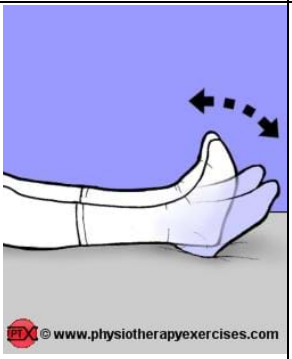	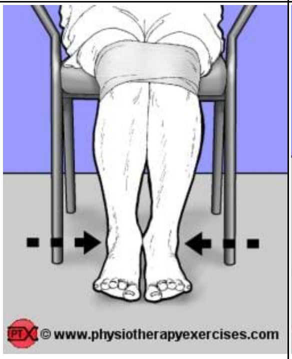	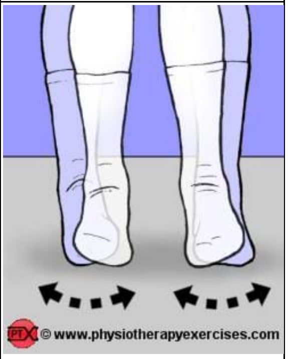

4.4 Strength training exercises for lower limb muscles (grade 3 and 4 strength)

**Hip muscles (grade 3 and 4)**			
Hip and knee flexion in supine	Hip flexor strengthening - lifting the foot while keeping the knee bent	Hip flexor strengthening in supine	Hip flexor strengthening in supine using weights
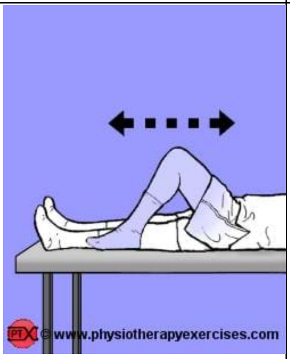	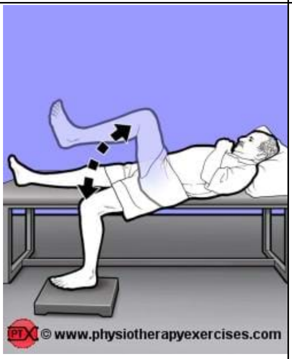	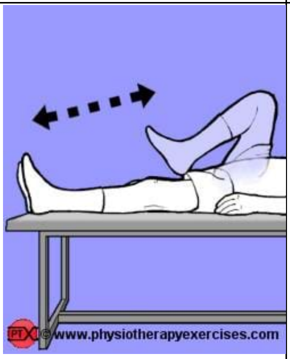	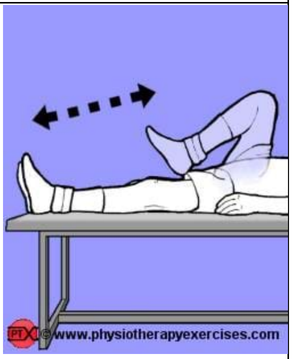

Straight leg raise with other knee bent	Straight leg raise	Hip flexor strengthening in sidelying using a slideboard and theraband	Hip flexor strengthening in supine using theraband
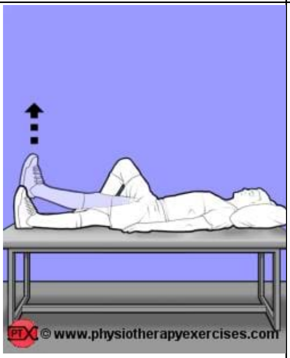	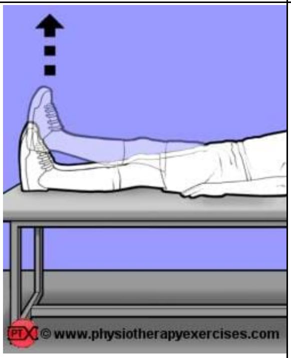	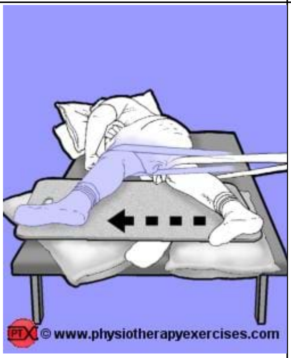	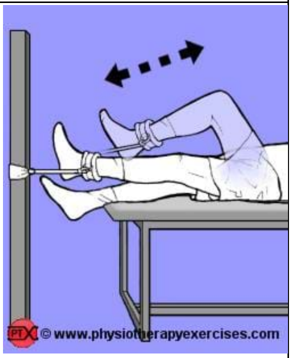

Hip flexor strengthening in supine using pulleys	Hip flexor strengthening in sitting	Hip flexor strengthening in sitting using scales	Hip flexor strengthening in sitting using a ball as a cue
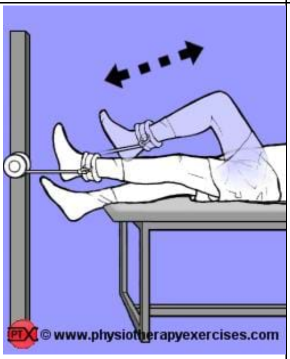	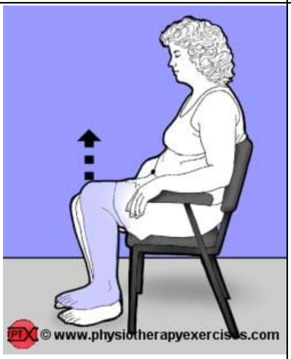	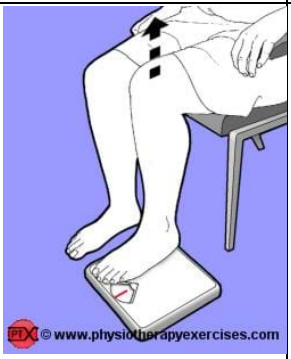	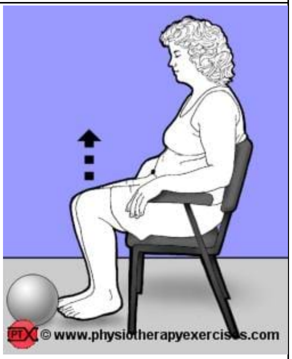

Hip flexor strengthening in sitting using weights	Hip flexor strengthening in standing	Hip flexor strengthening in standing using theraband	Hip flexor strengthening in standing using pulleys
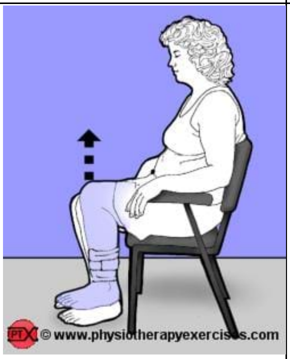	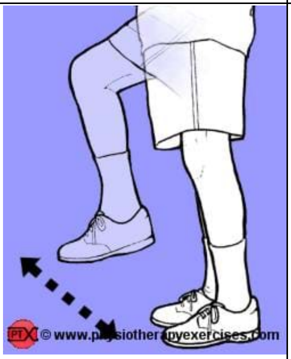	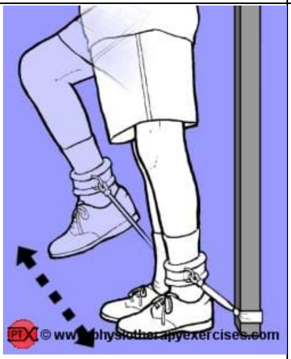	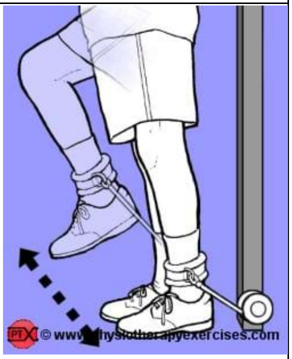

Hip flexor strengthening in standing using weights	Hip extensor strengthening in prone without weights	Hip extensor strengthening in prone using weights	Hip extensor strengthening in prone using theraband
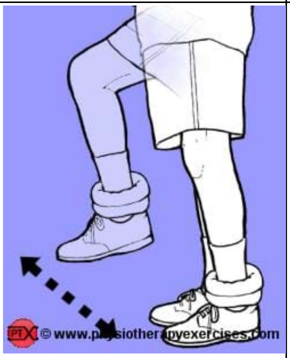	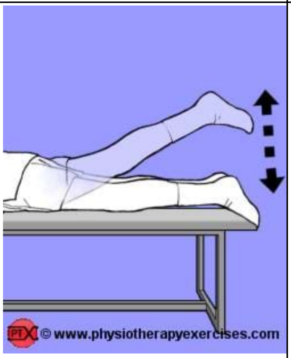	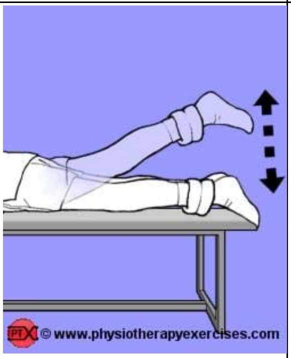	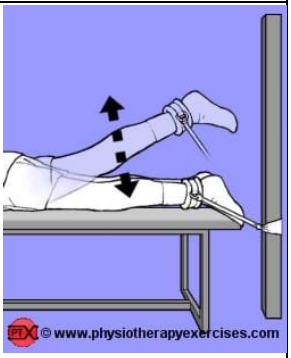

Hip extensor strengthening in prone using pulleys	Single-leg hip extensor strengthening in supine using digital scales	Single-leg hip extensor strengthening in supine	Bridging
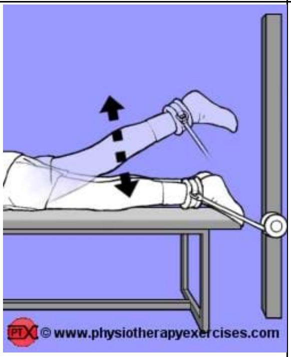	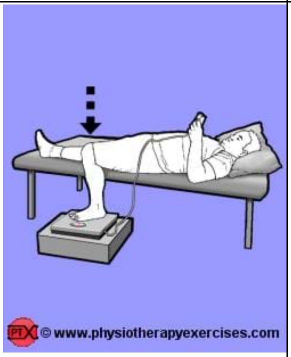	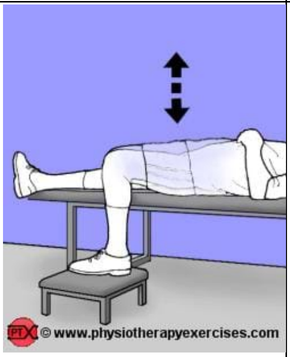	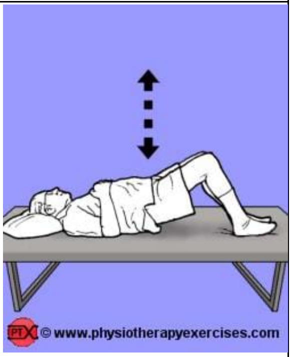

Bridging on a large ball	Hip extensor strengthening in standing	Hip extensor strengthening in standing using sandbag weights	Hip extension in standing against a wall
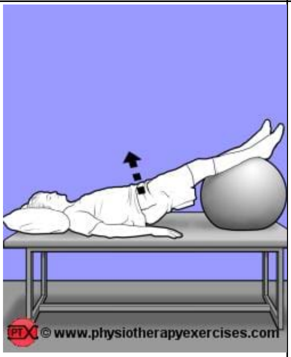	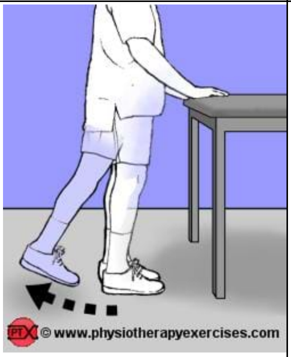	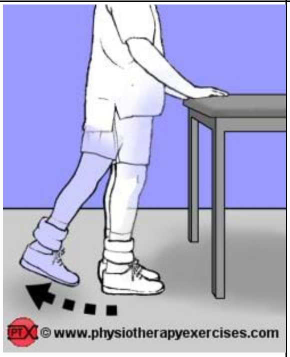	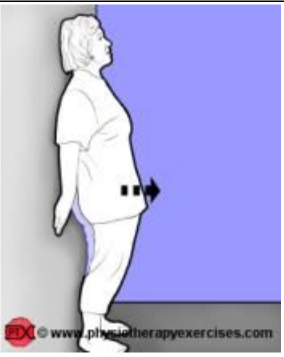

Hip abductor strengthening in sidelying against a wall	Hip abduction in sidelying	Hip abductor strengthening in sidelying using sandbag weights	Hip abductor strengthening in sidelying using theraband
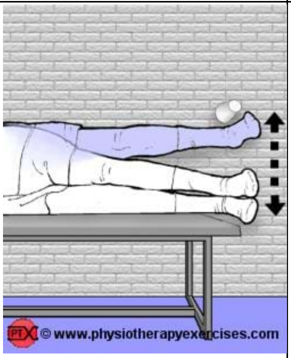	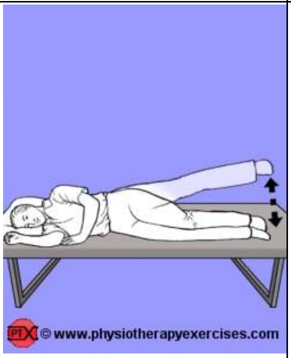	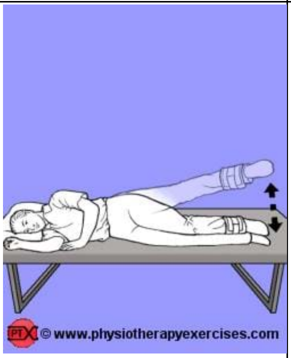	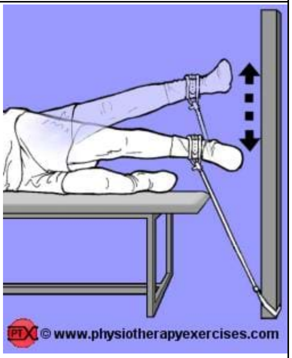

Hip abductor strengthening in sidelying using pulleys	Hip abductor strengthening in standing	Hip abductor strengthening in standing against a wall	Hip abductor strengthening in standing using sandbag weights
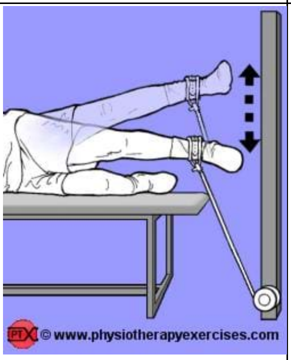	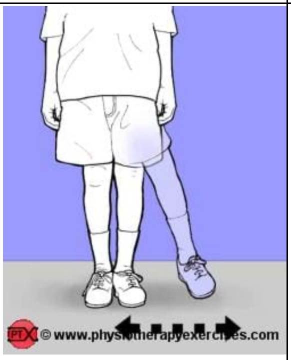	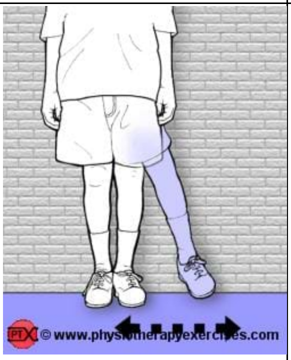	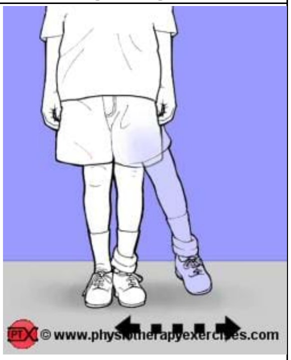

Hip abductor strengthening in standing using theraband	Hip abductor strengthening in standing using pulleys	Stepping sideways onto a block with hand support nearby	Lifting the leg sideways onto a block
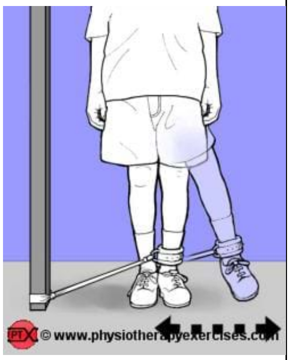	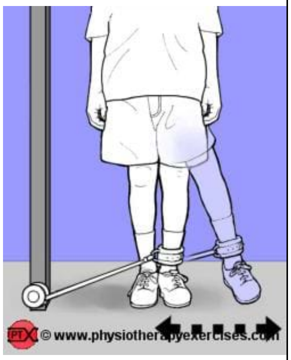	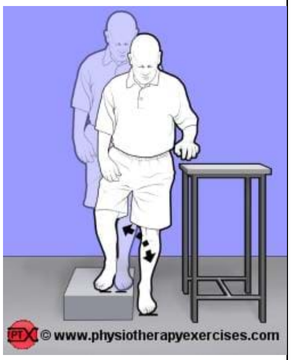	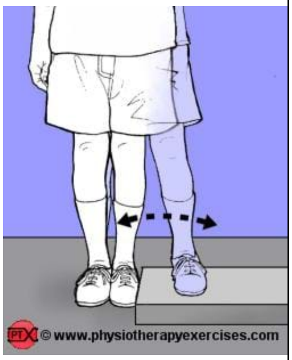

Hip adductor strengthening in sidelying using pulleys	Hip adductor strengthening in sidelying using theraband	Hip adductor strengthening in standing using theraband	Hip adductor strengthening in standing using pulleys
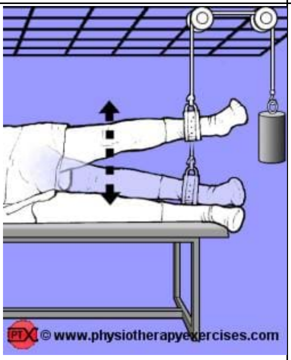	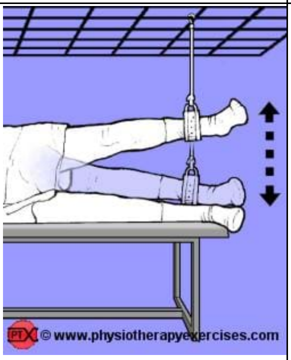	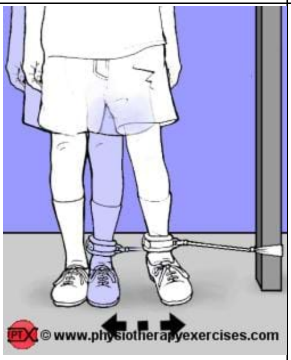	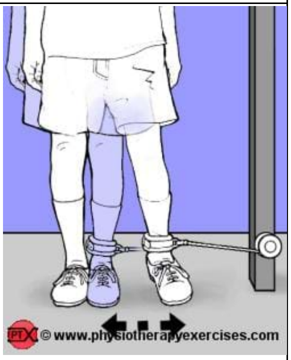

Hip external rotator strengthening in sidelying without weights	Hip external rotator strengthening in sitting using weights	Hip external rotator strengthening in sitting using theraband	Hip external rotator strengthening in sitting using pulleys
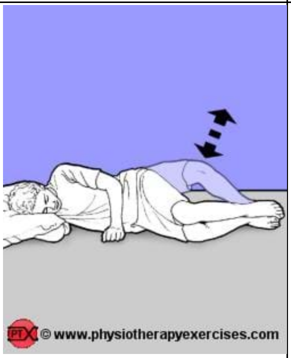	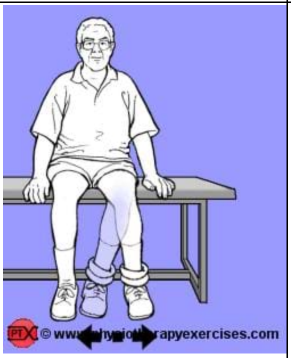	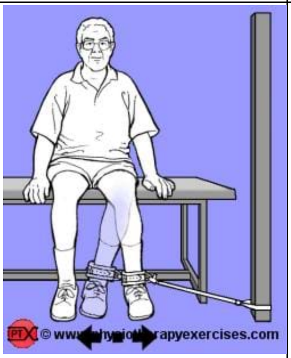	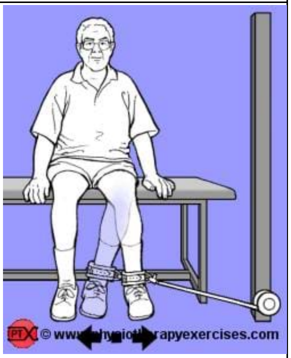

Internal hip rotator strengthening in sidelying without weights	Hip internal rotator strengthening in sitting using weights	Hip internal rotator strengthening in sitting using theraband	Hip internal rotator strengthening in sitting using pulleys
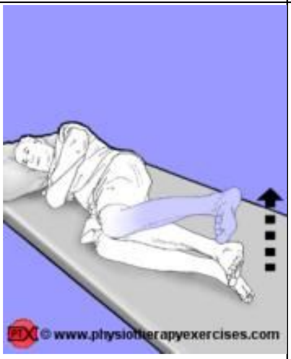	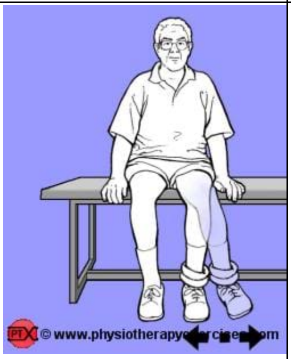	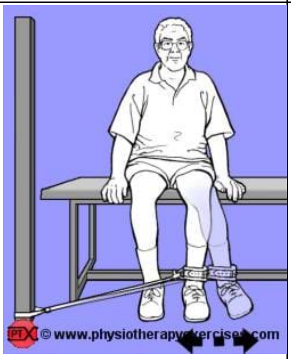	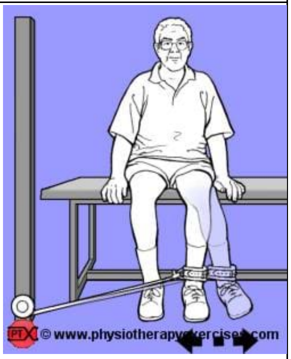

Knee muscles (grade 3 and 4)

Knee extensor strengthening in supine without weights	Knee extensor strengthening in supine using weights	Straight leg raise with other knee bent	Straight leg raise
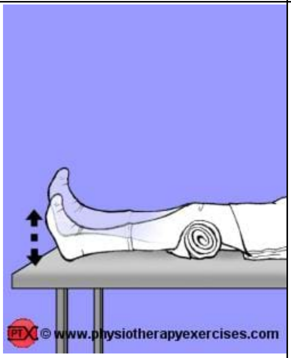	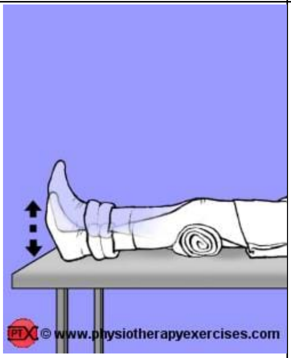	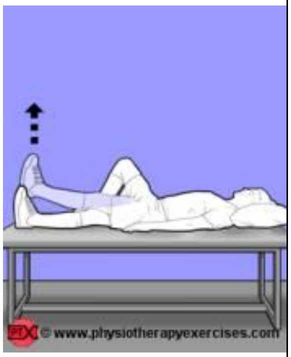	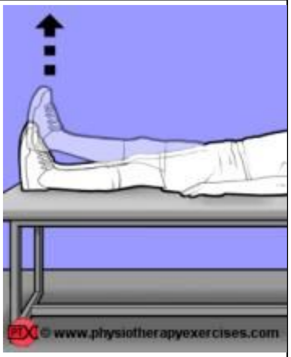

Knee extensor strengthening in sitting without weights	Knee extensor strengthening in sitting using weights	Knee extensor strengthening in sitting using theraband	Knee flexor strengthening in prone
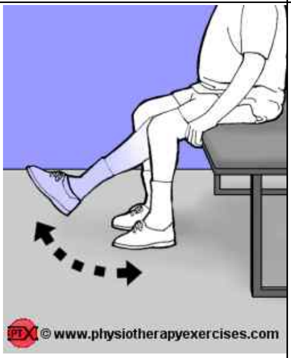	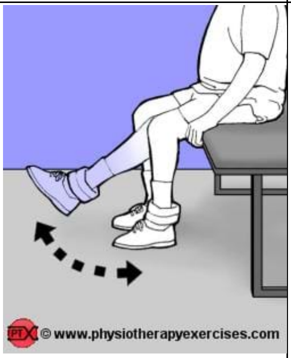	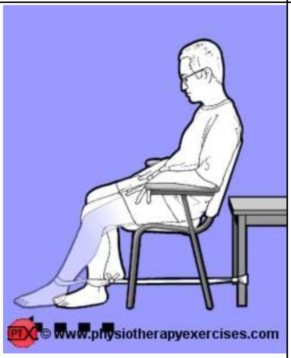	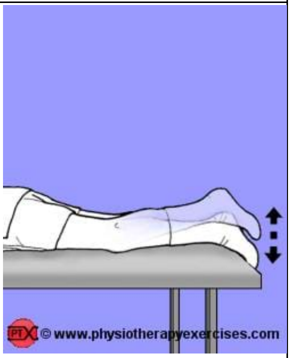

Knee flexion in prone	Knee flexor strengthening in prone using weights	Knee flexor strengthening in prone using theraband	Knee flexor strengthening in prone using pulleys
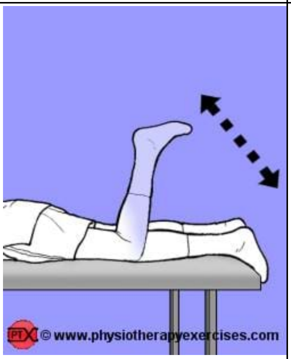	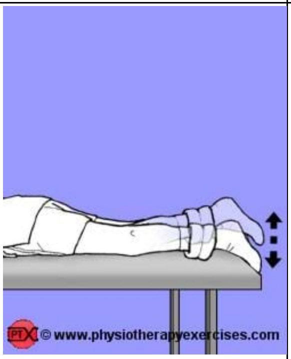	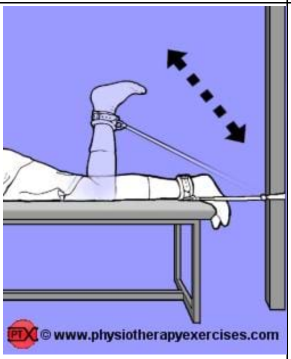	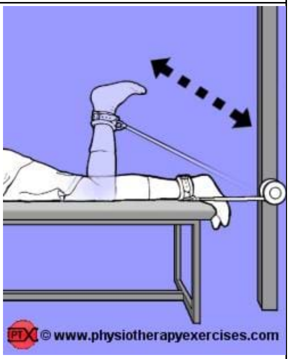

Knee flexor strengthening over the edge of a bed using theraband	Knee flexor strengthening in sitting using theraband	Knee flexor strengthening in supported standing without weights	Knee flexor strengthening in unsupported standing without weights
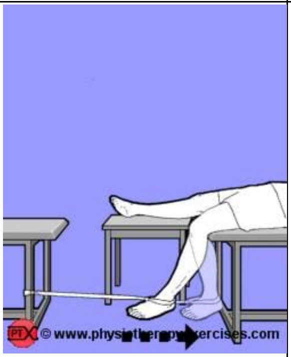	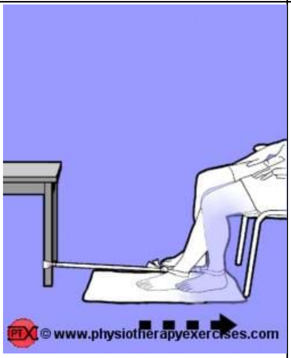	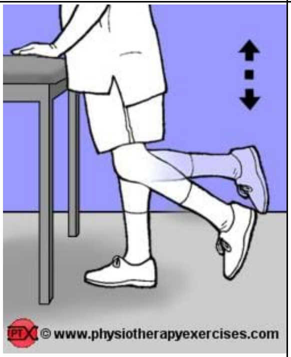	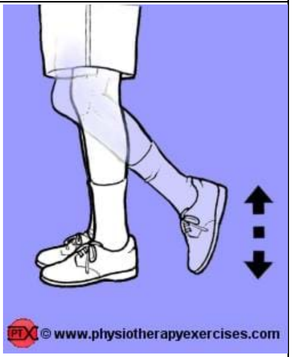

Knee flexor strengthening in standing using weights	Knee flexor strengthening in standing using pulleys	Knee flexor strengthening in standing using theraband	Seated walking
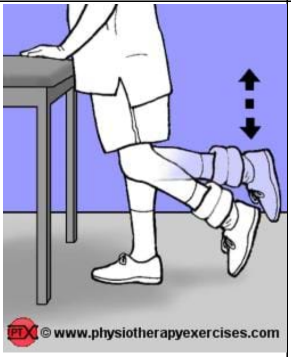	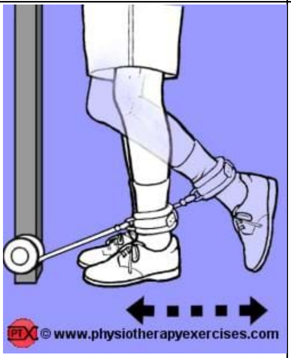	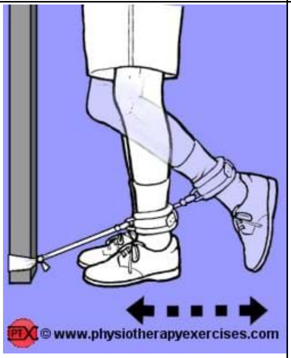	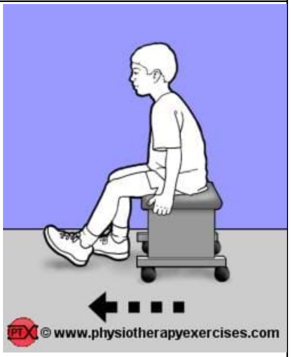

**Ankle muscles (grade 3 and 4)**			
Ankle dorsiflexor strengthening with theraband	Ankle dorsiflexor strengthening in sitting without weights	Ankle dorsiflexor strengthening in sitting using sandbag weights	Ankle plantarflexor strengthening in supine using theraband
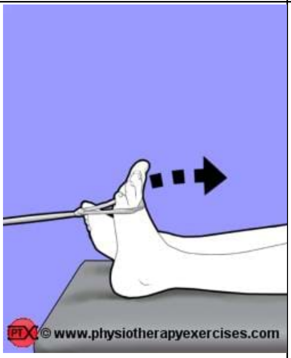	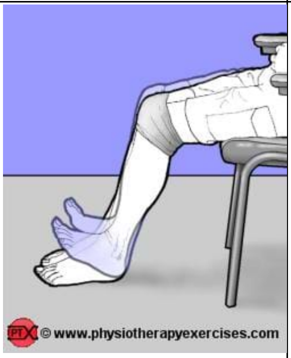	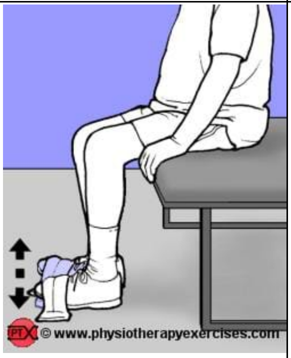	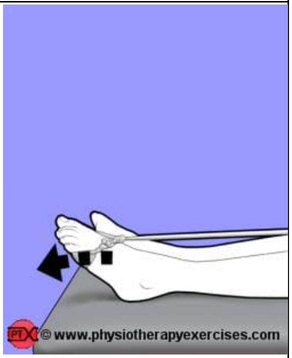

Ankle plantarflexor strengthening in sitting without weights	Ankle plantarflexor strengthening in sitting using sandbag weights	Ankle plantarflexor strengthening in sitting using theraband	Bilateral calf raises
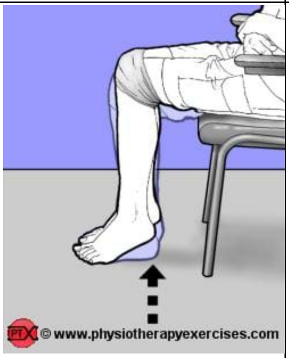	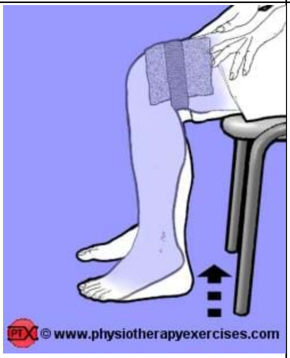	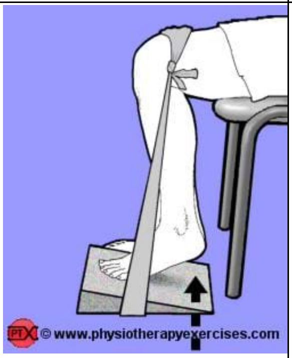	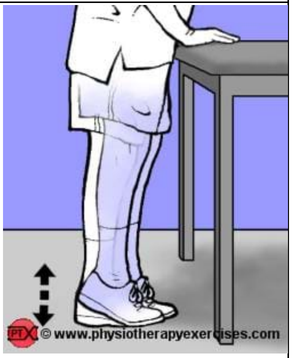

Moving from the heels to the toes in standing	Bilateral calf raises on a block	Single-leg calf raises	Single leg calf raises on a wedge
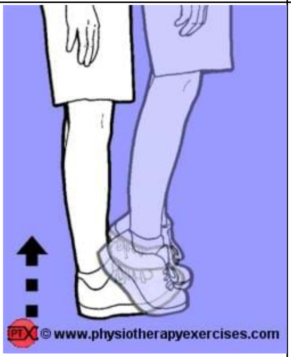	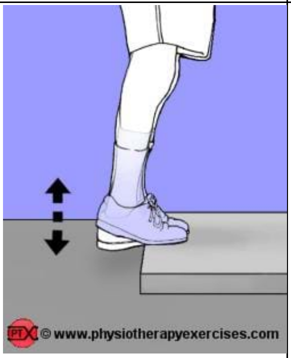	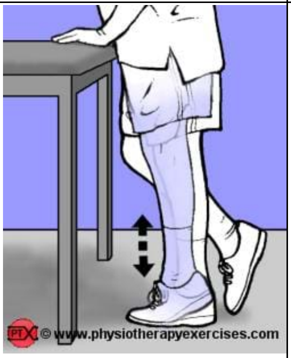	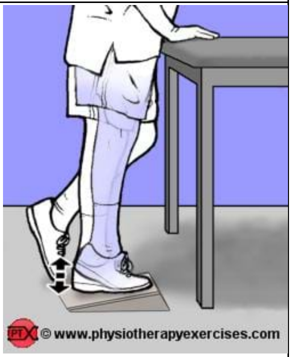

Ankle plantarflexor strengthening in standing using a wedge	Ankle evertor strengthening using theraband	Ankle invertor strengthening using theraband
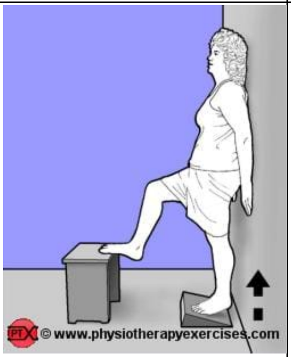	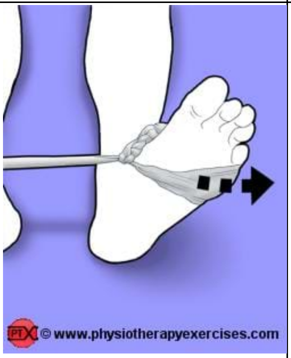	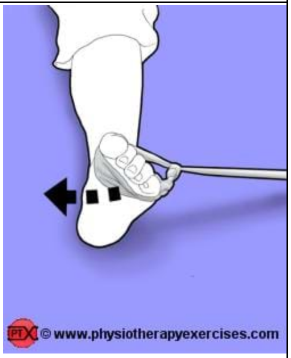

5 EXAMPLES OF 4 CASE STUDIES TO ILLUSTRATE THE ADDITIONAL MOTOR TRAINING THAT COULD BE PROVIDED IN A TYPICAL WEEK

Below are examples of four people with SCI. Each example includes:

- a manual muscle chart.
- 4 goals set as part of the baseline assessment (2 goals for 10 weeks and 6 months respectively).
- 2-4 weekly goals for the Additional Motor Training.
- examples of the types of exercises that could be provided as part of the Additional Motor Training.
- weekly Practice Sheets.

Additional Motor Training should be individualised to the needs of each participant and be primarily targeted as increasing participants' Total Motor Scores (primary outcome of the trial) as well as any/all secondary outcomes of the SCI-MT Trial (including the 4 goals set as part of the baseline assessment). It should involve active exercise (motor or strength training) of partially paralysed muscles.

In addition to the 4 goals set as part of the baseline assessment, therapists should set and record 2-4 specific goals for the Additional Motor Training each week on Practice Sheets. These goals are not study outcomes and are only intended to guide the Additional Motor Training. It is not expected that every exercise listed on a Practice Sheet is performed in every Additional Motor Training session. It is likely that a 2-hour session would include as few as 6 or as many as 20 exercises.

The Practice Sheets should capture:

- each exercise/activity (e.g. www.physiotherapyexercises.com, a note, a hand drawn stick figure, a photo or any other variation).
- intensity of practice for each exercise (amount of resistance/assistance, number of repetitions), and
- time spent on each activity.

Recording the time spent on each exercise/activity on the Practice Sheet will be important for helping you quickly and easily complete the "Case Report Form (CRF) for the Experimental Intervention". It is important that both the Practice Sheet and the CRFs are completed during or at the end of each session.

Note on all images used in this manual

- All images are copied with permission from www.physiotherapyexercises.com; freely available exercise-prescribing software designed for, and by, physiotherapists for people with injuries and disabilities.
- The images reflect physical presentations of people with different types of Neurological Impairments, and are not always representative of people with SCI. They are only intended to convey the principles of an exercise, and to be used as cues and prompts. They may need to be modified to the needs of each person.
- The titles of the exercises are the same as used in www.physiotherapyexercises.com but do not always fully convey the intention of the exercise.
- Further details about each exercise can be found on www.physiotherapyexercises.com including suggestions on how to make the exercise easier or harder. Use the title of

each exercise as “key words” to search. Then, right click on the matching image to see the full details.

5.1 Case 1: Person with a C5 neurological level and AIS C lesion

Manual Muscle Test:

UL Muscle group		Left/Right
Shoulder	FF	2
	E	2
	Abd	2
	Add	2
	IR	2
	ER	2
	Horiz add/abd	1
Elbow	F (C5)	3
	E (C7)	1
	Sup	1
	Pron	2
Wrist	F	2
	E (C6)	2
Finger	F (C8)	1
	E	2
	Abd (T1)/ Add	1
Thumb	F/E/Abd/Add/Opp	2

LL muscle group		Left/Right
Hip	F (L2)	2
	E	1
	Abd	1
	Add	2
	IR	2
	ER	1
Knee	F	2
	E (L3)	2
Ankle	PF (S1)	2
	DF (L4)	2
Toes	E (L5)/F	1

NB: Shaded table denotes key motor group for AIS

Examples of the 4 long-term goals set as part of the baseline assessment

10-week goals:

1. Bring toothbrush to the mouth without an aid with elbow supported by assistant, and brush teeth for 2 minutes.
2. Sit on the edge of a plinth with feet on the floor and lean forwards/sideways by 45 degrees, returning to centre, 20 times in 6 minutes without assistance.

6-month goals:

1. Step pivot transfer from wheelchair to bed with minimal assistance of one person and a slide-board within 4 minutes.
2. Type a set paragraph on a keyboard using a single finger without an aid within 6 minutes.

Supplementary file 1: Ben M, Glinsky JV, Spooren AI, Roberts S, Chen LW, Roberts S, Chen LW, Denis S, Lorusso M, Jorgensen V, Gollan EJ, Agostinello J, Van Laake-Geelen C, Lincoln C, Stolwijk JM, Bell C, Paddison S, Rainey D, Chu J, Tranter K, Ilha J, Oostra K, Sherrington C, Harvey LA. The Early and Intensive Motor Training Trial for People with Spinal Cord Injuries: Description of the Intervention. Spinal Cord. 2023

CASE 1: EXAMPLE OF A PRACTICE SHEET FOR ONE WEEK OF ADDITIONAL MOTOR TRAINING

Goals for the week

1. Bring right and left hand from chest level up to nose level whilst in supine position, with minimal assist at elbow.	2. Reach and tap an object placed on a table at elbow height, a reach angle of 45 deg and a reach distance of 15 cm twice in one minute.
3. Open and close fingers around a large cylinder/cup 3 times with 2 sec pauses without knocking over the cup.	4. Sit with arms by side on plinth, feet on floor, turn head to look behind to R and L 10 times with standby assistance of 1 person.

DATE:	Notes	Mon	Tues	Wed	Thurs	Fri	Sat	Sun
**Exercises aimed at improving UL function and strength**								
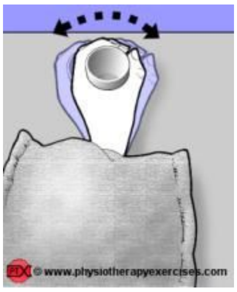	Cup secured to hand with micropore Markers placed at 2cm on either side of hand	20 touches out of 40 attempts 2 sets		22 touches out of 50 attempts 2 sets	20 touches out of 40 attempts 2 sets	20 touches out of 40 attempts 2 sets	23 touches out of 40 attempts 2 sets	
Wrist flexion/extension	**Time spent**	**5 mins**	**-**	**5 mins**	**5 mins**	**5 mins**	**5 mins**	**-**
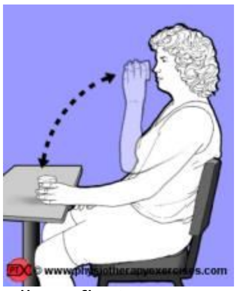	Starting position on a pillow at 45 deg flexion Assistance for concentric component, active eccentric	10 reps x 3 sets Best attempt = 3 second lowering to pillow;	10reps x 3sets as for Monday	10reps x 3sets as for Monday		100 reps on table, no gravity, full ROM, yellow th/band	100 reps on table, no gravity, full ROM, yellow th/band	Same setup as Monday 10reps x 3
Elbow flexion	**Time spent**	**5 mins**	**5 mins**	**5 mins**	**-**	**10 mins**	**10 mins**	**5 mins**
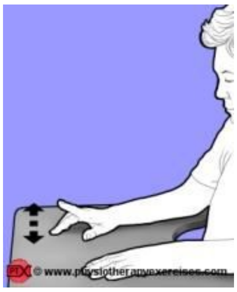	Place hand on foam and lift each finger up aiming to remove the indent under the finger and hold for 2 secs		50 reps x each finger 2 sets		50 reps x 2 sets	30 reps, 3 sets		60 reps 3 sets
Individual finger extension	**Time spent**	**-**	**10 mins**	**-**	**10 mins**	**10 mins**	**-**	**10 mins**
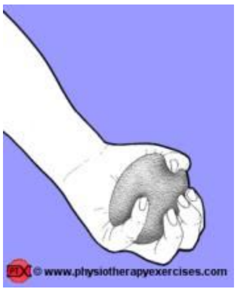	Squeeze a yellow stress ball	2-sec hold, 100 reps (done during LL session rest break)	2-sec hold, 100 reps	2-sec hold, 100 reps	2-sec hold, 80 reps	2-sec hold, 100 reps	2-sec hold, 110 reps	2-sec hold, 100 reps
Finger flexion	**Time spent**	**5 mins**	**5 mins**	**5 mins**	**5 mins**	**5 mins**	**5 mins**	**5 mins**

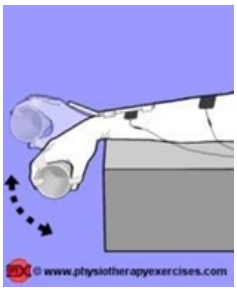	ES (50Hz, 35MA) +<br>active wrist extensors<br>to a straw target			30 reps<br>(to<br>fatigue) x<br>3 sets			30 reps<br>(to<br>fatigue) x<br>3 sets	30 reps<br>(to<br>fatigue) x<br>3 sets
Wrist extension	**Time spent**	**-**	**-**	**10 mins**			**10 mins**	**10 mins**
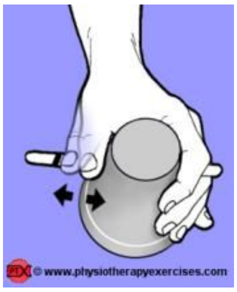	Marker on stick – 0.5<br>cm away from cup<br>Plastic cup		15 reps x<br>4 sets	15 reps x<br>4 sets		20 reps x<br>3 sets	20 reps x<br>4 sets	
Thumb abduction	**Time spent**	**-**	**5 mins**	**5 mins**	**-**	**5 mins**	**5 mins**	**-**
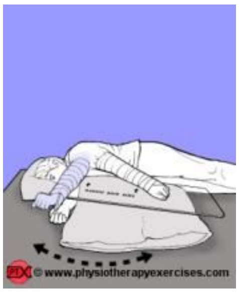	Board on 15 deg<br>angle, uphill for<br>flexion<br>Targets placed at sh<br>& hip<br>Slide sheet used	25 reps x<br>4 sets<br>No<br>assistanc<br>e<br>provided	20 reps x<br>5 sets<br>Min<br>assist for<br>sh extn<br>x10			20 reps x<br>3 sets<br><br>5 reps x<br>2 sets<br>w/out<br>slide<br>sheet		25 reps x<br>4 sets no<br>assistanc<br>e
Sh flexion/extension	**Time spent**	**10 mins**	**10 mins**	**-**	**-**	**10 mins**	**-**	**10 mins**
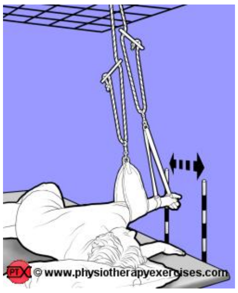	Yellow T/band<br>Targets placed 10 cm<br>above and below<br>neutral			50 reps<br>w 2-sec<br>pause on<br>each<br>end, 3x<br>1-min<br>rests	60 reps<br>w 1-sec<br>pause,<br>3xrests<br><br>15 reps<br>w 2-sec<br>pause		60 reps<br>w 1-sec<br>pause, 2<br>rests	40 reps<br>1-sec<br>pause, 3<br>rests
Sh flexion/extension to targets	**Time spent**	**-**	**-**	**10 mins**	**15 mins**	**-**	**10 mins**	**5 mins**
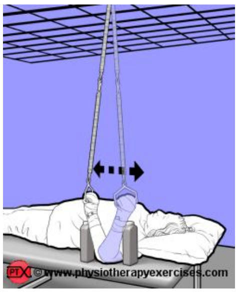	Yellow t/band<br>Targets placed to<br>20°IR/ER	100 reps<br>2sec<br>hold no<br>rests		100 reps<br>2sec<br>hold no<br>rests			Red<br>t/band<br>Assist for<br>concent<br>– active<br>Eccentric<br>.<br>Hold for<br>2 counts<br>10 reps x<br>3;<br>50with<br>yellow	
Sh ER/IR to targets	**Time spent**	**5 mins**	**-**	**5 mins**	**-**	**-**	**10 mins**	**-**

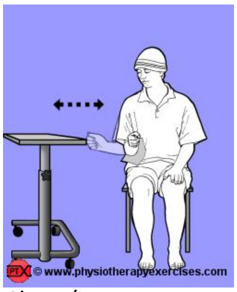 Sh ER/IR	Elbow supported on table with slide sheet under forearm Assistance provided to stop shoulder abduction/IR. Target @ 20° from neutral	30 reps x5 sets 1 min rests	30 repsx6 sets, 1 min rests		30 reps x5 sets 30 sec rests	35 reps x 4 sets 30 sec rests		35 reps x 3 sets
	**Time spent**	**10mins**	**10 mins**	**-**	**10mins**	**10 mins**	**-**	**10mins**
**Exercises aimed at improving lower limb function and strength**								
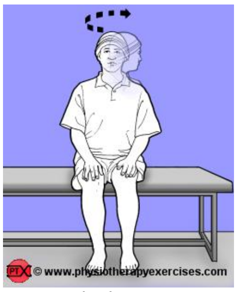 Sitting balance practice	Sit on edge of 50 cm height plinth hands on lap	2PTs for assistance Best time sitting unassisted 45 seconds		as on Monday best time 50 secs		Best attempt 50 secs	Best attempt 55 secs Could look towards right and then left x2	
	**Time spent**	**5 mins**	**-**	**5 mins**		**5 mins**	**5 mins**	
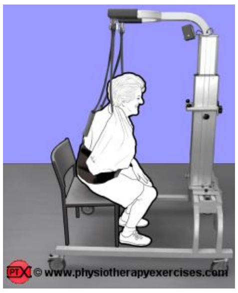 Sitting balance/STS	BWS harness applied to allow for sitting on edge of plinth and loading of LL's practice. No body weight support provided		Lean trunk forward by 10° and back to upright		Lean trunk forward by 10° and back to upright			Lean trunk forward by 15° and back to upright
	**Time spent**	**-**	**15 mins**	**-**	**15 mins**	**-**	**-**	**15 mins**
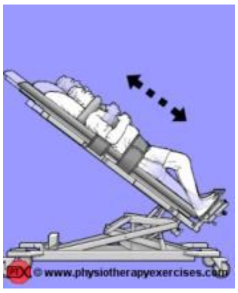 Knee/hip extension	Tilt table for knee/hip extension strengthening OR plantarflexor (PF) strengthening. Thin towel behind knees to avoid hyperextension	30° tilt. Knee control (flex/ext) 40 reps x 3 10 heel raises – assistance for concentric		30° tilt. Knee control (flex/ext) 40 reps x 3 10 heel raises – assistance for concentric		30° tilt. Knee control (flex/ext) 40 reps x 3 10 heel raises – assistance for concentric	35° tilt. Knee control (flex/ext) 35 reps x 3 8 heel raises – assistance for concentric	
	**Time spent**	**20 mins**	**-**	**20 mins**	**-**	**20 mins**	**20 mins**	**-**

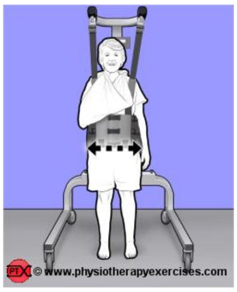 Weight bearing with BWS	BWS harness used for weight bearing/standing practice. Assistance from 2 PTs needed to facilitate knee extension		40% body weight support Isometric quads and glutes contractions		40% body weight support Isometric quads and glutes contractions			40% body weight support Isometric quads and glutes contractions Plus 10 x 10 weight shifts
	**Time spent**	**-**	**10 mins**	**-**	**10 mins**	**-**	**-**	**10 mins**
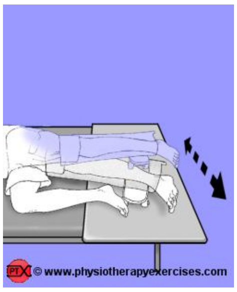 Hip flexion/extension	Pillows under board – flexion uphill, slide sheet Target placed at hip level	50 reps x 2sets Assist needed for hip extn	Use slings and yellow t/band 35 reps x 2		Use slings and yellow t/band 35 reps x 2			Use slings and yellow t/band 35 reps x 2
	**Time spent**	**10 mins**	**5 mins**	**-**	**5 mins**	**-**	**-**	**5 mins**
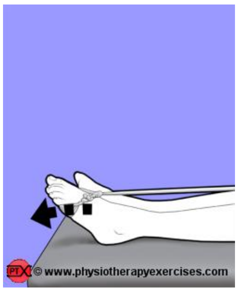 Ankle PF	Yellow TheraBand held by PT – even though Gr 2, able to perform against resistance when in supine and in gravity assisted position	15 reps x 3sets	15 reps x 3sets		17 reps x 3 sets			15 reps x 3
	**Time spent**	**5 mins**	**5 mins**	**-**	**5 mins**	**-**	**-**	**5 mins**
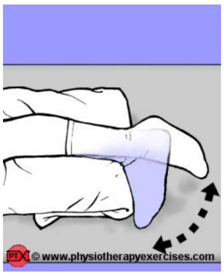 Ankle DF	Done in sitting with lower leg on opposite knee.	50 reps, 1-second pause	50 reps, 1-second pause	50 reps, 1-second pause	50 reps, 1-second pause	50 reps, 1-second pause	50 reps, 1-second pause	s50 reps, 1-second pause
	**Time spent**	**5 mins**	**5 mins**	**5 mins**	**5 mins**	**5 mins**	**5 mins**	**5 mins**
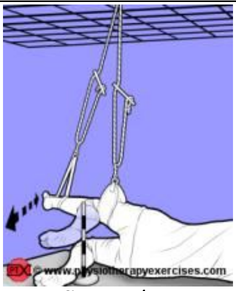 Knee flexion/extension	Target placed at 90° knee flexion and full extension		Yellow band manually applied for extension 15 reps x 3 sets	Yellow band manually applied for extension 15 reps x 3 sets	Yellow band manually applied for extension 15 reps x 3 sets		Focus on flexion – no TheraBand Target placed at 110 knee flexion 20 reps x 3	
	**Time spent**	**-**	**10 mins**	**10 mins**	**10 mins**		**10 mins**	

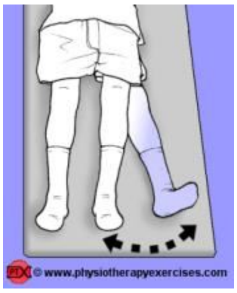	Target placed at 45° abduction.<br>Slidesheet placed under leg.<br>Assistance provided to stop hip ER	30 reps x3 sets Best result = 15 taps to target out of 30	30 reps x3 sets Best result = 13 taps to target out of 30	30 reps x3 sets Best result = 15 taps to target out of 30	Used slings instead of s/sheet 30x3, 20 taps			Used slings instead of s/sheet 30x3, 20 taps
Hip abduction	**Time spent**	**10 mins**	**10 mins**	**10 mins**	**10 mins**	**-**	**-**	**10 mins**
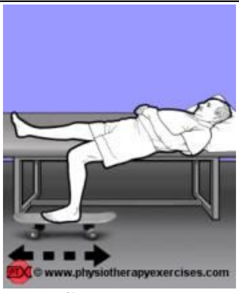	Plinth height adjusted to ensure foot in contact with floor.<br>Slide sheet used.<br>Targets placed at 70° -100° knee flexion ROM.<br>Mirror used to help patient see target	50 reps counting successful taps only				50 reps counting successful taps only		50 reps counting successful taps only
Knee flexion	**Time spent**	**5 mins**	**-**	**-**	**-**	**5 mins**	**-**	**5 mins**

5.2 Case 2: Person with a C5 neurological level and AIS D lesion

Manual Muscle Test:

UL Muscle Group		Left/Right
Shoulder	FF	4
	E	4
	Abd	4
	Add	3
	IR	3
	ER	3
	Horiz add/abd	3
Elbow	F (C5)	5
	E (C7)	3
	Sup	4
	pron	4
Wrist	F	4
	E (C6)	3
Finger	F (C8)	4
	E	3
	Abd (T1)/ Add	3
Thumb	F/E/Abd/Add/Opp	3

LL Muscle Group		Left/Right
Hip	F (L2)	4
	E	4
	Abd	3
	Add	3
	IR	2
	ER	3
Knee	F	3
	E (L3)	4
Ankle	PF (S1)	4
	DF (L4)	3
Toes	E (L5)/F	3

NB: Shaded table denotes key motor group for AIS

Examples of the 4 long-term goals set as part of the baseline assessment

10-week goals:

1. Walk 10 metres independently with/without a walking aid (+/- orthoses) in 20 seconds.
2. Be able to eat a bowl of soup/cereal with a spoon independently in under 5 minutes.

6-month goals:

1. Put on a shirt and button it up (7 buttons) independently in <2 minutes.
2. Walk up and then down a flight of stairs (12 stairs) independently with/without use of rail, with step-through gait in <3 minutes.

CASE 2: EXAMPLE OF A PRACTICE SHEET FOR ONE WEEK OF ADDITIONAL MOTOR TRAINING

Goals for the week

1. Stand up from a 65 cm high seat, return to sitting, with minimal assistance and UL use 10 times in 2 minutes.	2. Walk 20 metres over ground using a mobile BWS system with 20% of body weight supported, in 3 minutes.
3. Put on a short sleeve shirt with minimal assistance when sitting on edge of bed in < 2 minutes.	4. Pour water from a ¼ full cup to an empty cup and back, without spilling in under 1 minute.

DATE:	Notes	Mon	Tues	Wed	Thurs	Fri	Sat	Sun
**Exercises aimed at improving UL function and strength**								
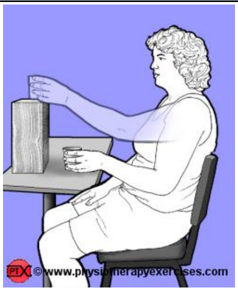 Transferring cup of water from table to a shelf	Empty plastic cup to start with. Place a "paddle pop" stick on top of cup – aim to avoid the cup tipping. 35cm box placed at 45° to the centre of patient	20 reps x 3 sets. Dropped stick 15 times in total	20 reps x 3 sets. Dropped stick 15 times in total		20 reps x 3 sets. Dropped stick 15 times in total			20 reps x 3 sets. Dropped stick 15 times in total
	**Time spent**	**5 mins**	**5 mins**	**-**	**5 mins**	**-**	**-**	**5 mins**
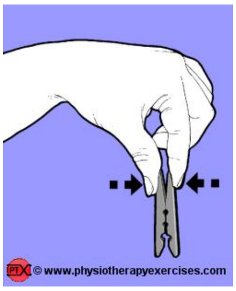 Pincer grip strengthening	Pick up pegs from tub and place them on string; return back to tub. Focus on pincer grip	Fastest time: 10 pegs in 60 secs 5 sets			8 pegs in 60 secs 5 sets		12 pegs in a row before dropping one 6 attempts	
	**Time spent**	**5 mins**	**-**	**-**	**5 mins**	**-**	**5 mins**	**-**
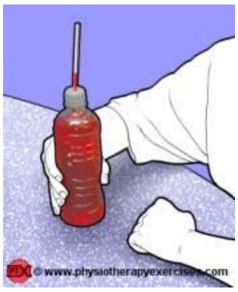 Finger/thumb flexion	Line marked at 5 cm on straw – aim to reach the line when squeezing the bottle. Use sports bottle that does not crush		Squeeze for 3 seconds without tipping sideways . 50 reps	Squeeze for 3 seconds without tipping sideways . 50 reps		Squeeze for 3 seconds without tipping sideways . 50 reps		Squeeze for 3 seconds without tipping sideways . 50 reps
	**Time spent**	**-**	**5 mins**	**5 mins**	**-**	**5 mins**	**-**	**5 mins**

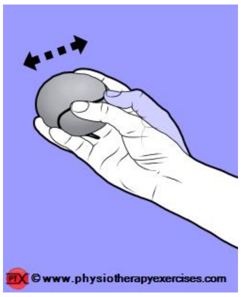 Thumb add/abduction	Tennis ball roll across fingers and back without dropping. Usually done during LL rests between sets	Continuous back and forth for 2 mins – dropped ball 15 times	Continuous back and forth for 2 mins – dropped ball 15 times	Continuous back and forth for 2 mins – dropped ball 7 times	Continuous back and forth for 2 mins – dropped ball 10 times	Continuous back and forth for 2 mins – dropped ball 15 times	Continuous back and forth for 2 mins – dropped ball 12 times	Continuous back and forth for 2 mins – dropped ball 15 times
	**Time spent**	**5 mins**	**5 mins**	**5 mins**	**5 mins**	**5 mins**	**5 mins**	**5 mins**
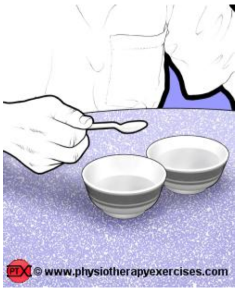 Transfer water b/w bowls	Tablespoon and large bowls used<br><br>Bowls set 10 cm apart. Aim to transfer water from one bowl to the other without spilling	Half bowl of water at the start. Spilled ~50%. Time taken 3 mins		Half bowl of water at the start. Spilled ~50%. Time taken 3 mins			Half bowl of water at the start. Spilled ~50%. Time taken 3 mins	
	**Time spent**	**5 mins**	-	**5 mins**	-	-	**5 mins**	-
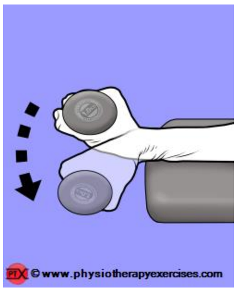 Wrist extension strength	Patient can do independent extension from full flexion to 90°. Assistance provided for remaining extension ROM. Independent eccentric component from full extension to flexion.	10 RM x 3 sets. Straw used for target of full extension	0.5kg, 10 RM x 3 sets.	0.5 kg, 10 RM x 3 sets.	0.5 kg, 10 RM x 3 sets.	0.5 kg, 10 RM x 3 sets	0.5 kg, 10 RM x 3 sets.	0.5 kg, 10 RM x 3sets
	**Time spent**	**5 mins**	**5 mins**	**5 mins**	**5 mins**	**5 mins**	**5 mins**	**5 mins**
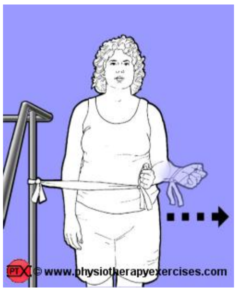 Sh ER	Yellow TheraBand used. Target placed at 20° from centre. Exercise done in sitting, no backrest		15RM x 3 sets with t/band	90° shoulder abd + ER with elbow support-ed; .5kg weight 10RM x 3sets	15RM x 3 sets with t/band		15RM x 3 sets with t/band	15RM x 3 sets with t/band
	**Time spent**	-	**5 mins**	**5 mins**	**5 mins**	-	**5 mins**	**5 mins**

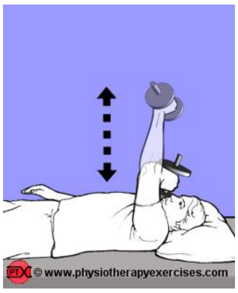	Start position 90° elbow flexion – assistance provided for concentric component if needed	0.5kg, 12 RM x 4 sets	0.5kg, 12 RM x 4 sets		0.5kg, 12 RM x 4 sets		0.5kg, 12 RM x 4 sets	0.5kg, 12 RM x 4 sets
Triceps strength	**Time spent**	**5 mins**	**5 mins**	**-**	**5 mins**	**-**	**5 mins**	**5 mins**
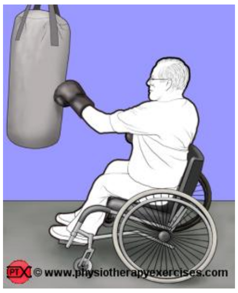	Boxing practice with therapist holding mitts; various combinations of punching exercises.			Mitts held at 90% arm length. Alternating L-R continuously for 1 min, 6 rounds		Mitts held at 90% arm length. Alternating L-R continuously for 1 min, 6 rounds		
Boxing/air boxing	**Time spent**	-	-	**10 mins**	-	**10 mins**	-	-
**Exercises aimed at improving LL function and strength**								
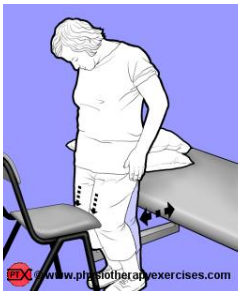	PT's hands used as targets, assistance for knee extension provided when needed; aim to avoid hyperextending knees	1min of practice: 15 episodes of h/extension. 3 sets			1min of practice: 15 episodes of h/extension. 3 sets			1min of practice: 15 episodes of h/extension. 3 sets
Knee control	**Time spent**	**5 mins**	**-**	**-**	**5 mins**	**-**	**-**	**5 mins**
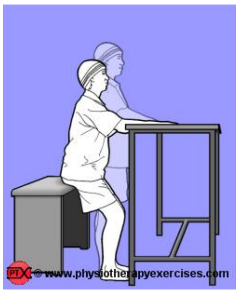	Start position on edge of 65-cm high plinth – PT's hands used as target for forward shoulder momentum. Aim to lift pelvis off seat, hold position, and return to sitting without UL use		Best attempt: 3-sec hold<br><br>20 reps x 3 sets	Best attempt: 5-sec hold<br><br>20 reps x 3 sets		Best attempt: 5-sec hold<br><br>20 reps x 3 sets	Best attempt: 5-sec hold<br><br>20 reps x 3 sets	
STS practice	**Time spent**	**-**	**5 mins**	**5 mins**	**-**	**5 mins**	**5 mins**	**-**
	Calf raises done in parallel bars or on tilt table	Tilt table @50° - single leg calf raise L and R.<br><br>12 reps x 3 sets		B/w parallel bars with minimal UL use. Bilat calf raise 15 reps x 3 sets			B/w parallel bars with minimal UL use. Bilat calf raise 15 reps x 3 sets	Tilt table @50° - single leg calf raise L and R.<br><br>12 reps x 3 sets

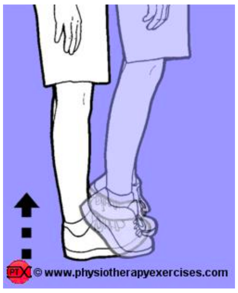 PF strengthening	**Time spent**	**5 mins**	**-**	**5 mins**	**-**	**-**	**5 mins**	**5 mins**
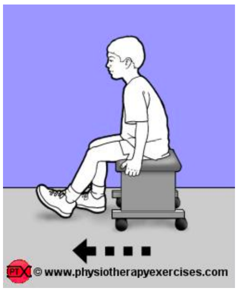 Target H/S endurance	Patient sitting in his manual wheelchair – aim to mobilise using LL's along the gym floor space; avoid swinging trunk back and forth.	10 metres, 30 "steps", 4 sets			10 metres, 30 "steps", 4 sets		10 metres, 30 "steps", 4 sets	
	**Time spent**	**5 mins**	**-**	**-**	**5 mins**	**-**	**5 mins**	**-**
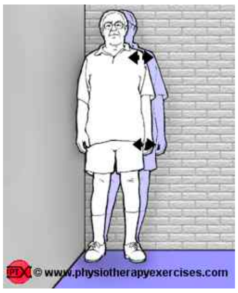 Lateral weight shifting in standing	B/w parallel bars aiming for hips to touch targets placed inside the bars during lateral shifts.	Occasional assistance from PT needed for knee control. UL used 50% of time<br>1 min x 6 sets		Practice 2 minutes in standing x 4		Practice 2 minutes in standing x 4		Practice 2 minutes in standing x 4
	**Time spent**	**10 mins**	**-**	**10 mins**	**-**	**10 mins**	**-**	**10 mins**
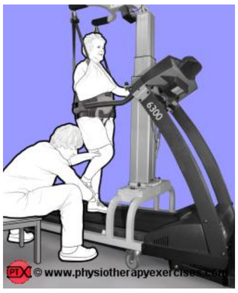 BWS treadmill training	Walking with BWS over treadmill		20% body weight support + assist for lateral weight shift. Speed 0.6 m/sec	20% body weight support + assist for lateral weight shift. Speed 0.6 m/sec		20% body weight support + assist for lateral weight shift. Speed 0.6 m/sec	20% body weight support + assist for lateral weight shift. Speed 0.6 m/sec	
	**Time spent**	**-**	**30 mins**	**30 mins**	**-**	**30 mins**	**30 mins**	**-**
	Walking with BWS over ground	20% BWS + assist x 1 PT at pelvis Walk 100 metres x 3			20% BWS + assist x 1 PT at pelvis Walk 100 metres x 3			20% BWS + assist x 1 PT at pelvis Walk 100 metres x 3

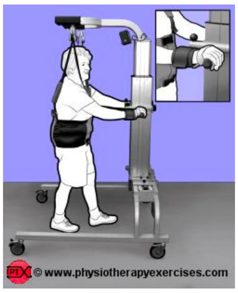 BWS over ground walking	**Time spent**	**20 mins**	**-**	**-**	**20 mins**	**-**	**-**	**20 mins**
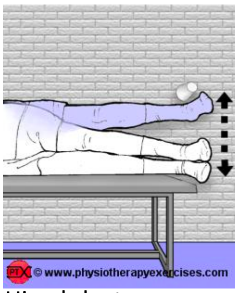 Hip abductor strengthening	TheraBand strapped around ankles. Target placed 20 cm above ankle	12 RM x 3 sets		12 RM x 3 sets		12 RM x 3 sets		12 RM x 3 sets
	**Time spent**	**5 mins**	**-**	**5 mins**	**-**	**5 mins**	**-**	**5 mins**
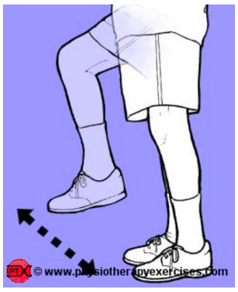 Hip flexion in standing – focus on standing LL	Aim to maintain stance leg extension and minimal pelvic drop whilst flexing knee/hip to a target. Minimal UL use encouraged	40 reps x 3 sets on L and R	40 reps x 3 sets on L and R		40 reps x 3 sets on L and R		40 reps x 3 sets on L and R	
	**Time spent**	**10 mins**	**10 mins**	**-**	**10 mins**	**-**	**10 mins**	**-**
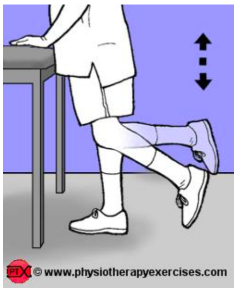 H/S strengthening in standing	Target placed at the level of knee height – aim to tap and hold as many times as possible until fatigued and cannot reach target *attempt resistance during every session, even for ¼ ROM – unsuccessful this week. That's why reps+++		90 reps x 2 sets		90 reps x 2 sets	90 reps x 2 sets		90 reps x 2 sets
	**Time spent**	**-**	**10 mins**	**-**	**10 mins**	**10 mins**	**-**	**10 mins**

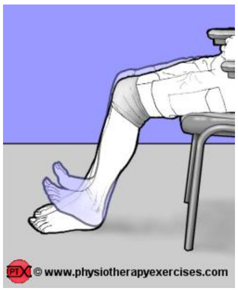<br><br>DF strengthening	Use a straw as a target	60 reps x 3 sets		60 reps x 3 sets			60 reps x 3 sets	
	**Time spent**	**10 mins**	-	**10 mins**	-	-	**10 mins**	-

5.3 Case 3: Person with a T10 neurological level and an AIS C lesion

Manual Muscle Test:

LL Muscle group		Left/Right
Hip	F (L2)	2
	E	1
	Abd	1
	Add	2
	IR	2
	ER	1
Knee	F	2
	E (L3)	2
Ankle	PF (S1)	2
	DF (L4)	2
Toes	E (L5)/F	1

NB: Shaded table denotes key motor group for AIS

Examples of the 4 long-term goals set as part of the baseline assessment

10-week goals:

1. To move/shuffle bottom across the length of a 60cm-high therapy plinth (without use of UL's) to the right and to the left with minimal assistance in under 6 minutes.
2. To walk 10 metres over ground using a mobile BWS system with 50% of body weight supported in 1 minute.

6-month goals:

1. To be able to get up from the floor with the use of the UL's and a walking aid without assistance in less than 2 minutes.
2. To be able to walk 500 metres with bilateral Canadian Crutches in 6 minutes without assistance or orthoses.

CASE 3: EXAMPLE OF A PRACTICE SHEET FOR ONE WEEK OF ADDITIONAL MOTOR TRAINING

Goals for the week

1. Sit unsupported on the edge of 55 cm high plinth and reach to the distance of 15 cm forwards, to the right and to the left 15 times with minimal assistance	2. 20 bilateral leg squats on tilt table at 45 degrees tilt without hands-on assistance within a 10-minute time frame
3. Stand with BWS harness and 30% of body weight supported, and shift weight to the right and then left without assistance	4. Stand in BWS harness, with 30% weight supported and bring right foot forward to a marker 10 cm in front and back to starting position, then left, without needing assistance.

DATE:	Notes	Mon	Tues	Wed	Thurs	Fri	Sat	Sun
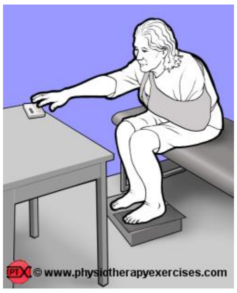	Target placed on table for patient to reach for. Scales placed under feet for feedback re lower limb loading. Seat height 50 cms.	100 reps, 2 sets 6kg on scales whilst reaching		100 reps, 2 sets 6kg on scales whilst reaching		120 reps, 2 sets 6kg on scales whilst reaching	100 reps, 2 sets 6kg on scales whilst reaching	
Reaching in sitting	**Time spent**	**10 mins**	-	**10 mins**	-	**10 mins**	**10 mins**	-
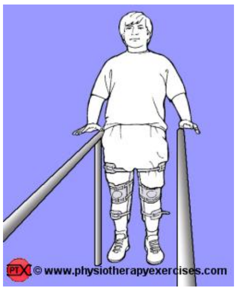	Assistance of 2 therapists (1 on each side) needed to assist with achieving knee and hip extension		UL use through out. Assist-ce removed from hips/ knees for max 30 secs	UL use through out Assist-ce removed from hips/ knees for max 30 secs		UL use through out Assist-ce removed from hips/ knees for max 30 secs		UL use through out Assist-ce removed from hips/ knees for max 30 secs
Standing b/w parallel bars with therapists' assistance	**Time spent**	-	**10 mins**	**10 mins**	-	**10 mins**	-	**10 mins**
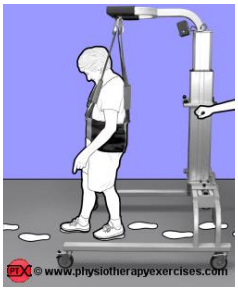	Body weight support provided; sock pulled over left foot – aim to step to a mark 10 cm in front and back. Repeat with right foot. Assistance required for weight shift	35% BWS. Assistance for knee extension on stance leg. 25 reps x 3 sets			35% BWS. Assistance for knee extension on stance leg. 25 reps x 3 sets		35% BWS. Assistance for knee extension on stance leg. 25 reps x 3 sets	35% BWS. Assistance for knee extension on stance leg. 25 reps x 3 sets
Weight bearing with BWS and stepping to a mark	**Time spent**	**10 mins**	-	-	**10 mins**	-	**10 mins**	**10 mins**

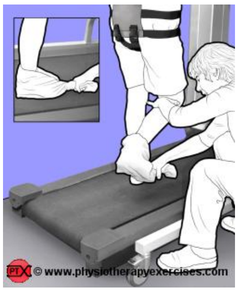 Treadmill walking with BWS and assistance	BWS treadmill training.<br>Pillowcase over for therapist to assist with foot swing on each leg.<br>3d therapist needed for lateral weight shift	40%BWS.<br>Speed 0.4m/sec<br>3 seated rests	40%BWS.<br>Speed 0.4m/sec<br>3 seated rests		40%BWS.<br>Speed 0.4m/sec<br>3 seated rests		40%BWS.<br>Speed 0.4m/sec<br>3 seated rests	40%BWS.<br>Speed 0.4m/sec<br>3 seated rests
	**Time spent**	**20 mins**	**20 mins**	-	**20 mins**	-	**20 mins**	**20 mins**
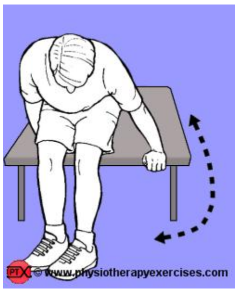 Lifting bottom up from seat – aim to load LL's	Aim to load LL's (not just UL's during lift)<br>Scales used for feedback re amount of weight borne through LL's		20 reps x 3<br><br>Lift pelvis up on the spot<br>15kg loaded onto scales		20 reps x 3<br><br>Lift pelvis up on the spot<br>15kg loaded onto scales	20 reps x 3<br><br>Lift pelvis up on the spot<br>15kg loaded onto scales		20 reps x 3<br><br>Lift pelvis up on the spot<br>15kg loaded onto scales
	**Time spent**	-	**10 mins**	-	**10 mins**	**10 mins**	-	**10 mins**
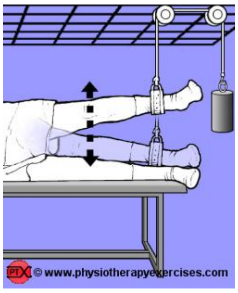 Hip abduction strengthening	2kg counter weight to allow active assisted abduction<br>Target placed 30 cm above knee ling	30 reps x 3 sets		30 reps x 3 sets		30 reps x 3 sets	30 reps x 3 sets	
	**Time spent**	**5 mins**	-	**5 mins**	-	**5 mins**	**5 mins**	-
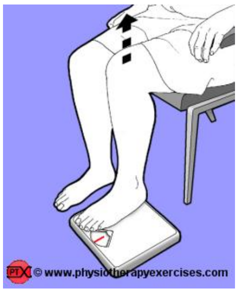 Hip flexion with scales	Reduce weight on scales by 2kgs – hold 2 secs		40 reps x 3 sets<br><br>Best attempt: reduce weight by 3kgs x 6	40 reps x 3 sets<br><br>Best attempt: reduce weight by 3kgs x 6	40 reps x 3 sets<br><br>Best attempt: reduce weight by 3kgs x 6			40 reps x 3 sets<br><br>Best attempt: reduce weight by 3kgs x 6
	**Time spent**	-	**10 mins**	**10 mins**	**10 mins**	-	-	**10 mins**

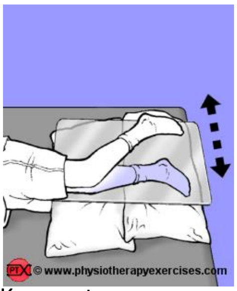	1kg ankle weight placed to add resistance. No slide sheet needed	45 reps x3 sets		45 reps x3 sets	45 reps x3 sets		45 reps x3 sets	
Knee extensors strengthening using targets	**Time spent**	**5 mins**	**-**	**5 mins**	**5 mins**	**-**	**5 mins**	**-**
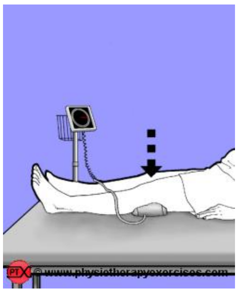	Isometric knee extension with inflated pressure cuff under knee. Aim to increase pressure under knee with each contraction		45 x 3sets Added pressure 60mm Hg	45 x 3sets Added pressure 60mm Hg		45 x 3sets Added pressure 60mm Hg		45 x 3sets Added pressure 60mm Hg
Isometric EOR quads	**Time spent**	**-**	**5 mins**	**5 mins**	**-**	**5 mins**	**-**	**5 mins**
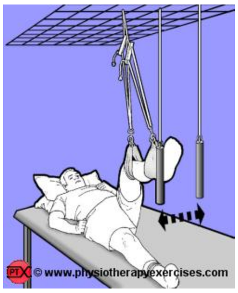	Targets placed 30 cm apart	100 reps x3 sets, 2-sec pause at each target		100 reps x3 sets, 2-sec pause at each target		100 reps x3 sets, 2-sec pause at each target		100 reps x3 sets, 2-sec pause at each target
Hip IR/ER strengthening	**Time spent**	**10 mins**	**-**	**10 mins**	**-**	**10 mins**	**-**	**10 mins**
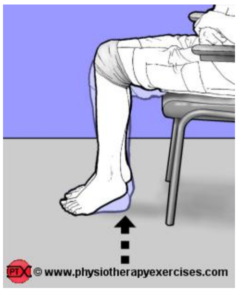	Foam placed under heel – aim to remove pressure from foam without lifting the ball of the foot up		20 reps x 3 sets		20 reps x 3 sets			20 reps x 3 sets
PF strengthening	**Time spent**	**-**	**5 mins**	**-**	**5 mins**	**-**	**-**	**5 mins**

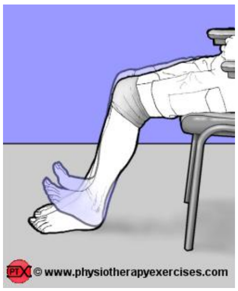	Patient holding a strap under foot for assisted concentric DF – release strap and focus on slow eccentric control without allowing foot slap back to floor	30 reps x 4 sets	30 reps x 4 sets	30 reps x 4 sets	30 reps x 4 sets	30 reps x 4 sets	30 reps x 4 sets	30 reps x 4 sets
DF strengthening	**Time spent**	**5 mins**	**5 mins**	**5 mins**	**5 mins**	**5 mins**	**5 mins**	**5 mins**
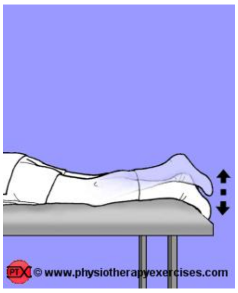	Start w pillow under lower leg Assistance provided for concentric – hold at 90°, controlled eccentric for 2 secs		25 reps x 4 sets		25 reps x 4 sets		25 reps x 4 sets	
H/S strengthening	**Time spent**	-	**10 mins**	-		-		-
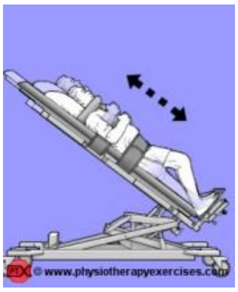	Tilt table for knee/hip extension strengthening OR plantarflexor (PF) strengthening. Thin towel behind knees to avoid hyperextension	30° tilt. Knee control (flex/ext) 40 reps x 3<br><br>10 heel raises – assist. for concentric		30° tilt. Knee control (flex/ext) 40 reps x 3<br><br>10 heel raises – assist-ce for concentri c		30° tilt. Knee control (flex/ext) 40 reps x 3<br><br>10 heel raises – assist-ce for concentri c	35° tilt. Knee control (flex/ext) 35 reps x 3<br><br>8 heel raises – assist-ce for concentri c	
Knee/hip extension	**Time spent**	**20 mins**	-	**20 mins**	-	**20 mins**	**20 mins**	-
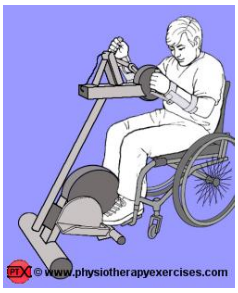	Motomed used for LL's in active servo cycle mode		Motor speed = 8rpm, active speed kept > 40 rpm; resistanc e level =1	Motor speed = 8rpm, active speed kept > 40 rpm; resistanc e level =1		Motor speed = 8rpm, active speed kept > 40 rpm; resistanc e level =1		
Active LL cycle with/without FES	**Time spent**	-	**20 mins**	**20 mins**	-	**20 mins**	-	-

5.4 Case 4: Person with a T10 neurological level and an AIS D lesion

Manual Muscle Test:

LL Muscle Group		Left/Right
Hip	F (L2)	4
	E	4
	Abd	3
	Add	3
	IR	3
	ER	3
Knee	F	3
	E (L3)	4
Ankle	PF (S1)	4
	DF (L4)	3
Toes	E (L5)/F	3

NB: Shaded table denotes key motor group for AIS

Examples of the 4 long-term goals set as part of the baseline assessment

10-week goals:

1. To stand up form a 50cm high plinth with minimal assistance from a therapist (but with no UL use) 10 times in 6 minutes.
2. To walk up and then down a 15-metre slope (gradient 1:4) with Canadian crutches, 3 times unassisted in 10 minutes.

6-month goals:

1. To jog over a 10-metre distance on grass without stopping and unassisted, in 4 seconds.
2. To walk 3 kms over flat (easy walk path) in the bushland unaided in 45 minutes with 1 sit-down rest.

CASE 4: EXAMPLE OF A TRAINING PROGRAM with a recording sheet

Goals for the week

1. Stand with feet together and look to the right, then to the left, return to centre, 5 times without using UL's for support.	2. Get up from a 55 cm seat height with minimal UL use 10 times in 2 minutes.
3. Standing b/w parallel bars, take a step to a line marked 30 cm in front and return to starting position, with right and then left foot, without needing UL support.	4. Walk 100 metres with Canadian Crutches and standby assistance in under 6 minutes.

DATE:	Notes	Mon	Tues	Wed	Thurs	Fri	Sat	Sun
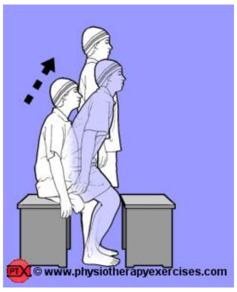 STS practice using target for knees forwards	Seat height – 60 cms. Manual guidance for shoulders forwards needed	30 reps x 4 sets	30 reps x 4 sets		40 reps x 3 sets			35 reps x 3 sets
	**Time spent**	**10 mins**	**10 mins**	**-**	**10 mins**	**-**	**-**	**10 mins**
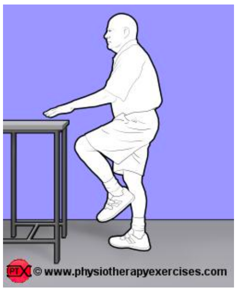 Hip flexion in standing	Close assistance of PT for maintenance of knee extension on stance leg	30 reps x 3 sets		30 reps x 3 sets		30 reps x 3 sets	30 reps x 3 sets	
	**Time spent**	**5 mins**	**-**	**5 mins**	**-**	**5 mins**	**5 mins**	**-**
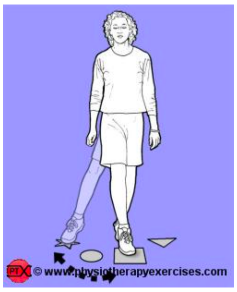 Toe taps to targets	Targets placed 20 cm in front, @45°, and @90°. 1 set = tap a target, return to centre, repeat for the 3 targets UL use required when returning to starting position		10 sets with L and then R	10 sets with L and then R		10 sets with L and then R		10 sets with L and then R
	**Time spent**	**-**	**10 mins**	**10 mins**	**-**	**10 mins**	**-**	**10 mins**
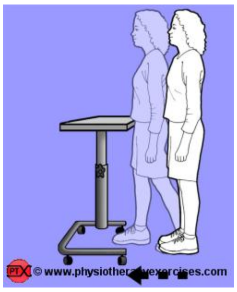 Forward stepping to target for hip extension	In parallel bars. Ruler placed at hip height as a target for forward hip moment. Target placed 25 cm from starting position	45 reps x 3	45 reps x 3		45 reps x 3		45 reps x 3	
	**Time spent**	**5 mins**	**5 mins**	**-**	**5 mins**	**-**	**5 mins**	**-**

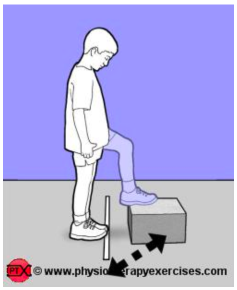	10 cm block used. Manual assistance for maintaining knee extension of stance leg required at times	20 foot taps x 4 sets; UL's used for support ~20% of time		20 foot taps x 4 sets; UL's used for support ~20% of time	20 foot taps x 4 sets; UL's used for support ~20% of time		20 foot taps x 4 sets; UL's used for support ~20% of time	20 foot taps x 4 sets; UL's used for support ~20% of time
Toe taps onto block facing forwards	**Time spent**	**5 mins**	**-**	**5 mins**	**5 mins**	**-**	**5 mins**	**5 mins**
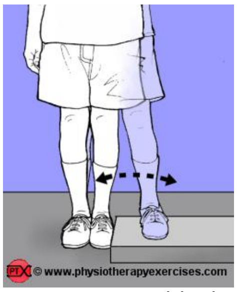	As above<br>Stance side next to wall to encourage minimal lateral trunk flexion		15 foot taps x 3 sets; UL's used for ~50% of time		15 foot taps x 3 sets; UL's used for ~50% of time	15 foot taps x 3 sets; UL's used for ~50% of time		15 foot taps x 3 sets; UL's used for ~50% of time
Foot taps onto block facing sideways	**Time spent**	**-**	**5 mins**	**-**	**5 mins**	**5 mins**	**-**	**5 mins**
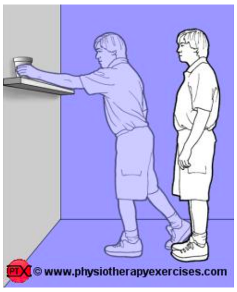	Bench within reach, no stepping forward yet. Aim to avoid using UL's during transfer of item from table (at waist height) to shelf (head height)	Use of UL's for balance with every 3-4 attempts	Use of UL's for balance with every 3-4 attempts		Use of UL's for balance with every 3-4 attempts		Use of UL's for balance with every 3-4 attempts	
Reaching in standing	**Time spent**	**15 mins**	**15 mins**	**-**	**15 mins**	**-**	**15 mins**	**-**
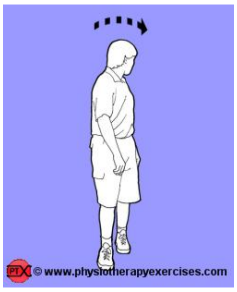	Stand with feet less than shoulder width apart. Look to the right – back to the middle – to the left.<br>Bench for UL support in front if needed – aim to avoid using UL's		Best attempt to maintain balance w/out UL use = 15 secs	Best attempt to maintain balance w/out UL use = 15 secs		Best attempt to maintain balance w/out UL use = 25 secs		Best attempt to maintain balance w/out UL use = 15 secs
Maintaining standing position	**Time spent**	**-**	**5 mins**	**5 mins**	**-**	**5 mins**	**-**	**5 mins**
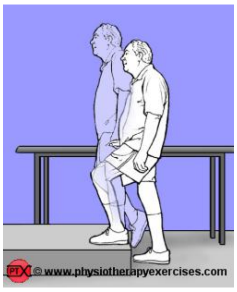	Forward **or** sideways step-ups onto 10 cm high block between parallel bars	10 reps x 3 sets forwards. Manual guidance for stepping knee control provided	5 reps x 5 sets sideways, manual guidance for knee control	11 reps x 3 sets forwards. Manual guidance for stepping knee control provided	5 reps x 5 sets sideways, manual guidance for knee control	10 reps x 3 sets forwards. Manual guidance for stepping leg knee control provided	7 reps x 5 sets sideways, manual guidance for knee control	12 reps x 3 sets forwards. Manual guidance for stepping knee control provided
Step-ups onto a step	**Time spent**	**10 mins**	**5 mins**	**10 mins**	**5 mins**	**10 mins**	**5 mins**	**10 mins**

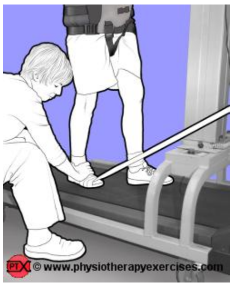 Treadmill walking	BWS harness applied, assistance of one PT for lateral weight shift required	15% body weight support Treadmill speed .8m/sec		15% body weight support Treadmill speed .8m/sec		15% body weight support Treadmill speed .8m/sec	15% body weight support Treadmill speed .8m/sec	15% body weight support Treadmill speed .8m/sec
	**Time spent**	**30 mins**	**-**	**30 mins**	**-**	**30 mins**	**30 mins**	**30 mins**
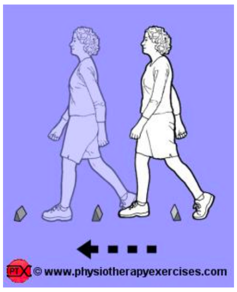 Overground walking	Walk between parallel bars, stepping over obstacles		Walk back and forth 10 times 3 obstacles (ankle weight sandbag) placed on floor		Walk back and forth 10 times 3 obstacles (ankle weight sandbag) placed on floor			
	**Time spent**	**-**	**5 mins**	**-**	**5 mins**	**-**	**-**	**-**
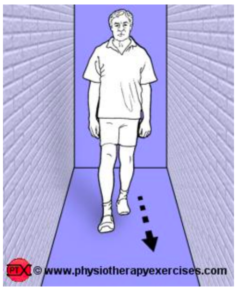 Overground walking	Walking in the gym with assistance of 2 PTs or use of Canadian Crutches and one PT		Walk total of 100 metres 3 sit down rests required (2PTs)	Walk total of 60 metres 2 sit down rests required (2 PTs)	Walk total of 120 metres with 2 CC's and 1PT (4 sit down rests required)	Walk total of 60 metres 2 sit down rests required	Walk total of 80 metres 3 sit down rests required	Walk total of 100 metres 3 sit down rests required
	**Time spent**	**-**	**10 mins**	**10 mins**	**15 mins**	**10 mins**	**10 mins**	**10 mins**
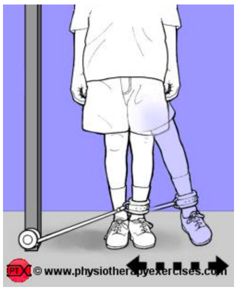 Hip abduction strength	Lowest weight on pulley system (.5kg). UL support required.	12 reps x 3 sets	12 reps x 3 sets	12 reps x 3 sets		12 reps x 3 sets	12 reps x 3 sets	
	**Time spent**	**5 mins**	**5 mins**	**5 mins**	**-**	**5 mins**	**5 mins**	**-**
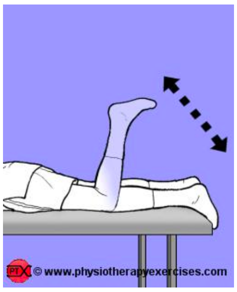 Knee flexion strength	Start position with foot on pillow to reduce ROM. Assistance for concentric contraction provided when fatigues.		20 reps x 3 sets		20 reps x 3 sets	20 reps x 3 sets		20 reps x 3 sets
	**Time spent**	**-**	**5 mins**	**-**	**5 mins**	**5 mins**	**-**	**5 mins**

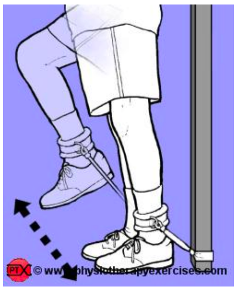	2.5kg weight on pulley system. UL's used for support in standing.		12 reps x 3 sets	12 reps x 3 sets		12 reps x 3 sets		12 reps x 3 sets
Hip flexion strength	**Time spent**	**-**	**5 mins**	**5 mins**	**-**	**5 mins**	**-**	**5 mins**
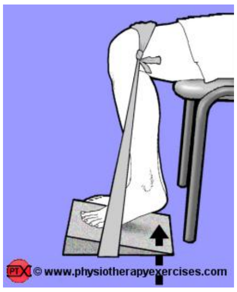	Soleus strengthening with red TheraBand.<br><br>Gastrocnemius strengthening in standing: raise up on both feet, lift L leg up and return to standing via R foot with focus on eccentric control. Repeat on other side	Soleus:<br>12 reps x 3 sets<br><br>Gastrocs:<br>15 reps x 3 sets	Soleus:<br>12 reps x 3 sets<br><br>Gastrocs:<br>15 reps x 3 sets		Soleus:<br>12 reps x 3 sets<br><br>Gastrocs:<br>15 reps x 3 sets		Soleus:<br>12 reps x 3 sets<br><br>Gastrocs:<br>15 reps x 3 sets	
PF strength	**Time spent**	**10 mins**	**10 mins**	**-**	**10 mins**	**-**	**10 mins**	**-**

6 PRACTICE SHEETS FOR INTERVENTION PARTICIPANTS ONLY

6.1 Purpose:

- To help therapists structure the Additional Motor Training provided to Intervention Participants.
- To ensure that the Additional Motor Training is administered as per the protocol.
- To ensure the Additional Motor Training is progressed.
- To ensure therapists and participants focus on the intensity of therapy.
- To enable transfer of therapy between two or more therapists.
- To ensure weekly or bi-weekly goals are set and reviewed.
- To help maintain motivation for therapists and participants.
- To provide some record of what Additional Motor Training was provided.
- To help therapists accurately complete the Intervention record.

6.2 Instructions for therapists:

- This is a suggested template only. Therapists are free to vary this provided the top part of each form is the same and provided the form captures:
    a. weekly goals. These are to be set in addition to those already set as part of the baseline assessment for attainment at 10 weeks and 6 months
    b. all exercises administered
    c. the time a participant spends on an exercise each day (only include active time, not time spent in set-up, chat or rest)
    d. the number of repetitions of an exercise ***if*** it is feasible to count
    e. some measure of the intensity or difficulty of an exercise ***if*** feasible e.g. resistance, amount of assistance, BORG scale, weight lifted etc
- Notes can be added to help explain an exercise (particularly important if more than one therapist is treating a participant).
- The image/description of the exercises can be in any format including:
    a. An image from www.physiotherapyexercises.com
    b. A hand-drawn stick figure
    c. Text to explain the exercises

**PARTICIPANT INITIALS:**		**THERAPIST NAME:**	
**PARTICIPANT ID:**	- (site number) (participant ID)	**WEEK STARTING:**	- - (dd) (mm) (yyyy)

PRACTICE SHEET (for Intervention Participants only)

Goals for the week

**1.**	**2.**
**3.**	**4.**

Image/ description of exercise	Notes	Mon	Tues	Wed	Thurs	Fri	Sat	Sun
1.								
		Time (min)	Time (min)	Time (min)	Time (min)	Time (min)	Time (min)	Time (min)
2.								
		Time (min)	Time (min)	Time (min)	Time (min)	Time (min)	Time (min)	Time (min)
3.								
		Time (min)	Time (min)	Time (min)	Time (min)	Time (min)	Time (min)	Time (min)

PARTICIPANT INITIALS: | | |

THERAPIST NAME:

PARTICIPANT ID: | | | - | | |
(site number) (participant ID)

WEEK STARTING: | | - | | - | | | |
(dd) (mm) (yyyy)

Image/ description of exercise	Notes	Mon	Tues	Wed	Thurs	Fri	Sat	Sun
4.								
		Time (min)	Time (min)	Time (min)	Time (min)	Time (min)	Time (min)	Time (min)
5.								
		Time (min)	Time (min)	Time (min)	Time (min)	Time (min)	Time (min)	Time (min)
6.								
		Time (min)	Time (min)	Time (min)	Time (min)	Time (min)	Time (min)	Time (min)
7.								
		Time (min)	Time (min)	Time (min)	Time (min)	Time (min)	Time (min)	Time (min)

PARTICIPANT INITIALS:				THERAPIST NAME:	
PARTICIPANT ID:	-			WEEK STARTING:	- -
	(site number)	(participant ID)			(dd) (mm) (yyyy)

Image/ description of exercise	Notes	Mon	Tues	Wed	Thurs	Fri	Sat	Sun
8.								
		Time (min)	Time (min)	Time (min)	Time (min)	Time (min)	Time (min)	Time (min)
9.								
		Time (min)	Time (min)	Time (min)	Time (min)	Time (min)	Time (min)	Time (min)
10.								
		Time (min)	Time (min)	Time (min)	Time (min)	Time (min)	Time (min)	Time (min)
11.								
		Time (min)	Time (min)	Time (min)	Time (min)	Time (min)	Time (min)	Time (min)

PARTICIPANT INITIALS: | | |

PARTICIPANT ID: | | | - | | |
(site number) (participant ID)

THERAPIST NAME:

WEEK STARTING: | | - | | - | | | |
(dd) (mm) (yyyy)

Image/ description of exercise	Notes	Mon	Tues	Wed	Thurs	Fri	Sat	Sun
12.								
		Time (min)	Time (min)	Time (min)	Time (min)	Time (min)	Time (min)	Time (min)
13.								
		Time (min)	Time (min)	Time (min)	Time (min)	Time (min)	Time (min)	Time (min)
14.								
		Time (min)	Time (min)	Time (min)	Time (min)	Time (min)	Time (min)	Time (min)
15.								
		Time (min)	Time (min)	Time (min)	Time (min)	Time (min)	Time (min)	Time (min)

PARTICIPANT INITIALS: | | |

PARTICIPANT ID: | | | - | | | (site number) (participant ID)

THERAPIST NAME:

WEEK STARTING: | | - | | - | | | | (dd) (mm) (yyyy)

Image/ description of exercise	Notes	Mon	Tues	Wed	Thurs	Fri	Sat	Sun
16.								
		Time (min)	Time (min)	Time (min)	Time (min)	Time (min)	Time (min)	Time (min)
17.								
		Time (min)	Time (min)	Time (min)	Time (min)	Time (min)	Time (min)	Time (min)
18.								
		Time (min)	Time (min)	Time (min)	Time (min)	Time (min)	Time (min)	Time (min)
19.								
		Time (min)	Time (min)	Time (min)	Time (min)	Time (min)	Time (min)	Time (min)

PARTICIPANT INITIALS: | | |

PARTICIPANT ID: | | | - | | | (site number) (participant ID)

THERAPIST NAME:

WEEK STARTING: | | - | | - | | | | (dd) (mm) (yyyy)

Image/ description of exercise	Notes	Mon	Tues	Wed	Thurs	Fri	Sat	Sun
20.								
		Time (min)	Time (min)	Time (min)	Time (min)	Time (min)	Time (min)	Time (min)
21.								
		Time (min)	Time (min)	Time (min)	Time (min)	Time (min)	Time (min)	Time (min)
22.								
		Time (min)	Time (min)	Time (min)	Time (min)	Time (min)	Time (min)	Time (min)
23.								
		Time (min)	Time (min)	Time (min)	Time (min)	Time (min)	Time (min)	Time (min)

7 APPENDICES

7.1 Lists of treatments and therapies that are considered appropriate and inappropriate

Below is a list of treatment and therapies that are considered appropriate and inappropriate. This list is intended as a guide and there may be circumstances where a treatment or therapy that is listed as inappropriate could be used sparingly. For example, a short stretch of the PFs may be required prior to standing/walking to reap the benefits of a transient increase in DF. Overall, therapies must involve the active contraction (or attempts at active contraction) of neurologically weak muscles at and below the level of the injury. Some therapies that may reasonably supplement a treatment and are considered appropriate (e.g., somatosensory stimulation provided during attempts at movement).

Appropriate	Inappropriate
Strength training	Massage
Skill training	Electrotherapy
Treadmill training	Passive movements
ES upper and lower limb cycling (provided the participant is actively contributing)	Equipment prescription
ES during strength training	Wheelchair skill training
ES during task training	Upper limb strength training for a person with incomplete paraplegia
Game based therapies	Stretch
Somatosensory stimulation e.g., median nerve stimulation with UL task training	Hydrotherapy
BWS standing and gait training +/- treadmill	Pain management e.g., TENS
Robotics (with active contribution from participant)	Respiratory management
Mental imagery (with active contribution from participant)	Group therapy sessions
Active UL ergometry and LL cycling	Fitness training
OT sessions such as cooking or ADL training involving the UL for a person with tetraplegia	Education

8 REFERENCES

1. The National Health and Medical Research Council, the Australian Research Council and, the Australian Vice-Chancellors' Committee. National Statement on Ethical Conduct in Human Research. 2007 (Updated May 2018). Commonwealth of Australia.
2. International Council for Harmonisation of technical requirements for pharmaceuticals for human use (ICH). ICH Harmonised Guideline: Integrated addendum to ICH E6(r1): Guideline For Good Clinical Practice. 2016.
